# Association between vitamin D deficiency and major depression in patients with chronic kidney disease: a cohort study

**DOI:** 10.3389/fnut.2025.1540633

**Published:** 2025-01-27

**Authors:** I-Wen Chen, Wei-Ting Wang, Yi-Chen Lai, Ying-Jen Chang, Yao-Tsung Lin, Kuo-Chuan Hung

**Affiliations:** ^1^Department of Anesthesiology, Chi Mei Medical Center, Liouying, Tainan City, Taiwan; ^2^School of Medicine, College of Medicine, National Sun Yat-sen University, Kaohsiung City, Taiwan; ^3^Department of Anesthesiology, E-Da Hospital, I-Shou University, Kaohsiung City, Taiwan; ^4^Department of Anesthesiology, Chi Mei Medical Center, Tainan City, Taiwan

**Keywords:** vitamin D deficiency, chronic kidney disease, depression, risk factor, nutrition

## Abstract

**Background:**

Patients with chronic kidney disease (CKD) have an elevated risk of both vitamin D deficiency (VDD) and depression. However, the relationship between VDD and the risk of depression in this population remains unclear.

**Methods:**

Using the TriNetX network database (2010–2019), we conducted a propensity score-matched cohort study of CKD patients aged ≥50 years. Patients were categorized into VDD (≤20 ng/mL) and control (≥30 ng/mL) groups based on measurements within 3 months of CKD diagnosis. The primary outcome was the incidence of major depression within 1 year of follow-up.

**Results:**

Among 17,955 matched pairs, VDD was associated with increased depression risk at 1 year (hazard ratio [HR]: 1.929; 95% confidence interval [CI]: 1.52–2.448; *p* < 0.0001). This association persisted through 3 years of follow-up. The relationship remained consistent across CKD stages, with similar risks in early (HR:1.977; 95% CI: 1.382–2.829) and CKD stage 3–5 (HR:1.981; 95% CI: 1.533–2.559). Males with VDD showed higher depression risk (HR: 2.264; 95% CI: 1.498–3.421) compared to females (HR:1.761; 95% CI: 1.307–2.374). Even vitamin D insufficiency (20–30 ng/mL) increased depression risk compared to normal levels (HR:1.667; 95% CI: 1.318–2.11). In patients with VDD, cerebrovascular disease, malnutrition, and ischemic heart disease are risk factors for depression.

**Conclusion:**

VDD is independently associated with increased depression risk in patients with CKD, particularly in males. These findings suggest that maintaining adequate vitamin D levels might be important for mental health in patients with CKD, although randomized trials are needed to confirm whether supplementation can prevent depression in this population.

## 1 Introduction

Chronic kidney disease (CKD), characterized by a gradual loss of kidney function over time, affects approximately 10% of the global population (1–3). This high prevalence of CKD can be attributed to an increasingly aging population, along with a growing prevalence of type 2 diabetes mellitus, obesity, hypertension, and cardiovascular diseases, all of which significantly contribute to CKD development and progression (4). The progression of CKD is linked to several severe complications, such as a higher incidence of anemia, metabolic bone disease, cardiovascular events, increased mortality, and greater utilization of healthcare resources (5, 6). In addition, major depression is also a common complication in patients with CKD, with an overall prevalence of 34.0% (7). This high prevalence of depression not only impairs quality of life but also contributes to adverse health outcomes, including increased hospitalization rates, reduced treatment adherence, and higher mortality risk (8–11). Notably, depression has been identified as a risk factor for accelerated decline in kidney function (11–13), creating a detrimental cycle in which depression and CKD progression may exacerbate each other. Recent studies have suggested that depression-associated inflammation, medication non-adherence, and unhealthy behaviors may contribute to the faster deterioration of kidney function (12, 14, 15), highlighting the critical importance of early depression prevention in patients with CKD.

The kidneys play a crucial role in vitamin D metabolism by converting 25-hydroxyvitamin D to its active form, 1,25-dihydroxyvitamin D (16). As kidney function declines, this conversion is impaired, leading to decreased vitamin D levels.

The prevalence of vitamin D deficiency (VDD) was high among patients with CKD, progressively increasing from 40.7% in stage 3 to 61.5% in stage 4, and reaching 85.7% in stage 5 as renal function declined (17). Beyond its well-known role in bone and mineral metabolism, recent evidence suggests that vitamin D may play a role in mood regulation, with low vitamin D levels being associated with depression (18–22).

The extremely high prevalence of VDD among patients with CKD, along with their inherently increased risk of depression, presents a potentially modifiable risk factor that deserves thorough investigation. Therefore, we conducted a large-scale cohort study using real-world data to investigate whether VDD is associated with an increased risk of depression in patients with CKD who have elevated baseline depression risk. These findings could have important implications for depression prevention strategies in patients with CKD and might suggest new therapeutic approaches for this vulnerable population.

## 2 Methods

### 2.1 Data source and ethics statement

This study used data from the TriNetX network, a comprehensive federated health research platform that connects electronic health records from 133 healthcare institutions worldwide. The TriNetX database comprises de-identified patient information, encompassing a wide range of clinical data, such as patient demographics, diagnoses, procedures, prescribed medications, and laboratory test results. The study period spanned from 2010 to 2019, encompassing a large-scale real-world patient population. The study protocol was reviewed and approved by the Chi Mei medical center Institutional Review Board (IRB number: 11310-E04). As the study used only de-identified data, the requirement for individual patient consent was waived in accordance with the standard research practices for retrospective database studies. This cohort study was conducted in accordance with the Strengthening the Reporting of Observational Studies in Epidemiology (STROBE) guidelines for observational research.

### 2.2 Study population and eligibility criteria

The study included patients aged ≥50 years who had at least two healthcare organization visits documented in the system and were diagnosed with CKD. Patients were required to have vitamin D level measurements within the first 3 months following their CKD diagnosis. This age threshold was chosen because older adults have an increased risk of VDD due to reduced skin synthesis, decreased outdoor activities, and impaired renal function, while also being more susceptible to depression due to multiple comorbidities and social factors. Vitamin D measurements were required within the first 3 months following CKD diagnosis to establish a baseline vitamin D status that would accurately reflect the patient’s condition at CKD diagnosis, minimizing the potential confounding effects of disease progression and therapeutic interventions. Based on these measurements, patients were categorized into two groups: the VDD group with serum vitamin D levels below 20 ng/mL and the control group with levels above 30 ng/mL. To avoid bias, patients assigned to the VDD group were excluded if they had any occurrence of vitamin D levels >20 ng/mL within 3 months of their CKD diagnosis. Similarly, patients assigned to the control group were excluded if they had any occurrence of vitamin D levels below 30 ng/mL within 3 months of diagnosis.

To minimize potential confounding factors, we excluded patients with a history of cognitive impairment, dementia, Alzheimer’s disease, Parkinson’s disease, vascular dementia, schizophrenia, bipolar disorder, major depression, or substance use disorders prior to CKD diagnosis. We also excluded patients with previous stroke, intracranial hemorrhage, or head injury, as well as those who died within 3 years of CKD diagnosis. The information that includes the cohort definitions, query criteria, codes, and analysis setup was available in Supplementary file 1.

### 2.3 Propensity score matching

To reduce selection bias and ensure balanced baseline characteristics between the VDD and control groups, we conducted propensity score matching in a 1:1 ratio. This matching process incorporated demographic information, comorbidities, and clinical parameters to achieve comparable groups and improve the reliability of the study outcomes. The matching variables included age at index date, sex (male/female), race, body mass index, and factors influencing health status and contact with health services. Comorbidities incorporated in the matching included essential (primary) hypertension, diabetes mellitus, overweight and obesity, ischemic heart disease, neoplasm, liver disease, cerebrovascular disease, malnutrition, and hyperparathyroidism. We also included CKD stage and laboratory parameters (hemoglobin, albumin, and glomerular filtration rate) in the propensity score calculation.

### 2.4 Outcomes

The primary outcome was the development of major depression within 1 year after the index date of CKD diagnosis. Depression was identified using diagnostic codes in electronic health records. As secondary outcomes, we assessed the cumulative incidence of depression at two and 3 years of follow-up to evaluate the long-term association between VDD and depression risk.

### 2.5 Additional analysis

We conducted a series of supplementary analyses to evaluate the robustness and consistency of the findings. First, we performed subgroup analyses stratified by sex and CKD severity (stages 1–2 vs. stages 3–5) to assess potential effect modification. Second, we investigated a possible dose-dependent relationship by comparing depression risk between patients with vitamin D insufficiency (20–30 ng/mL) and those with normal vitamin D levels (≥30 ng/mL). Finally, we conducted a logistic regression analysis to identify independent risk factors for depression in our population with VDD.

### 2.6 Statistical analysis

All analyses were performed using the TriNetX Analytics Platform, which provides a web-based interface for the real-time analysis of electronic health record data. Baseline characteristics between the VDD and control groups were compared using standardized differences before and after propensity score matching, with values <0.1 considered to indicate good balance between groups. Continuous variables are expressed as mean ± standard deviation, and categorical variables are presented as numbers and percentages. The association between VDD and depression was assessed using Cox proportional hazards models within the TriNetX platform, with the results presented as hazard ratios (HRs) with 95% confidence intervals (CIs). Cumulative incidence curves were generated using the Kaplan–Meier method, and differences between groups were evaluated using the log-rank test. For subgroup analyses, we performed stratified Cox proportional hazards analyses according to sex and CKD severity. To identify independent risk factors for depression in patients with VDD, we performed both univariate and multivariate logistic regression analyses, with results presented as odds ratios (ORs) with 95% CIs. A two-sided *p*-value <0.05 was considered statistically significant for all analyses.

## 3 Results

### 3.1 Patient selection

**Figure 1:**
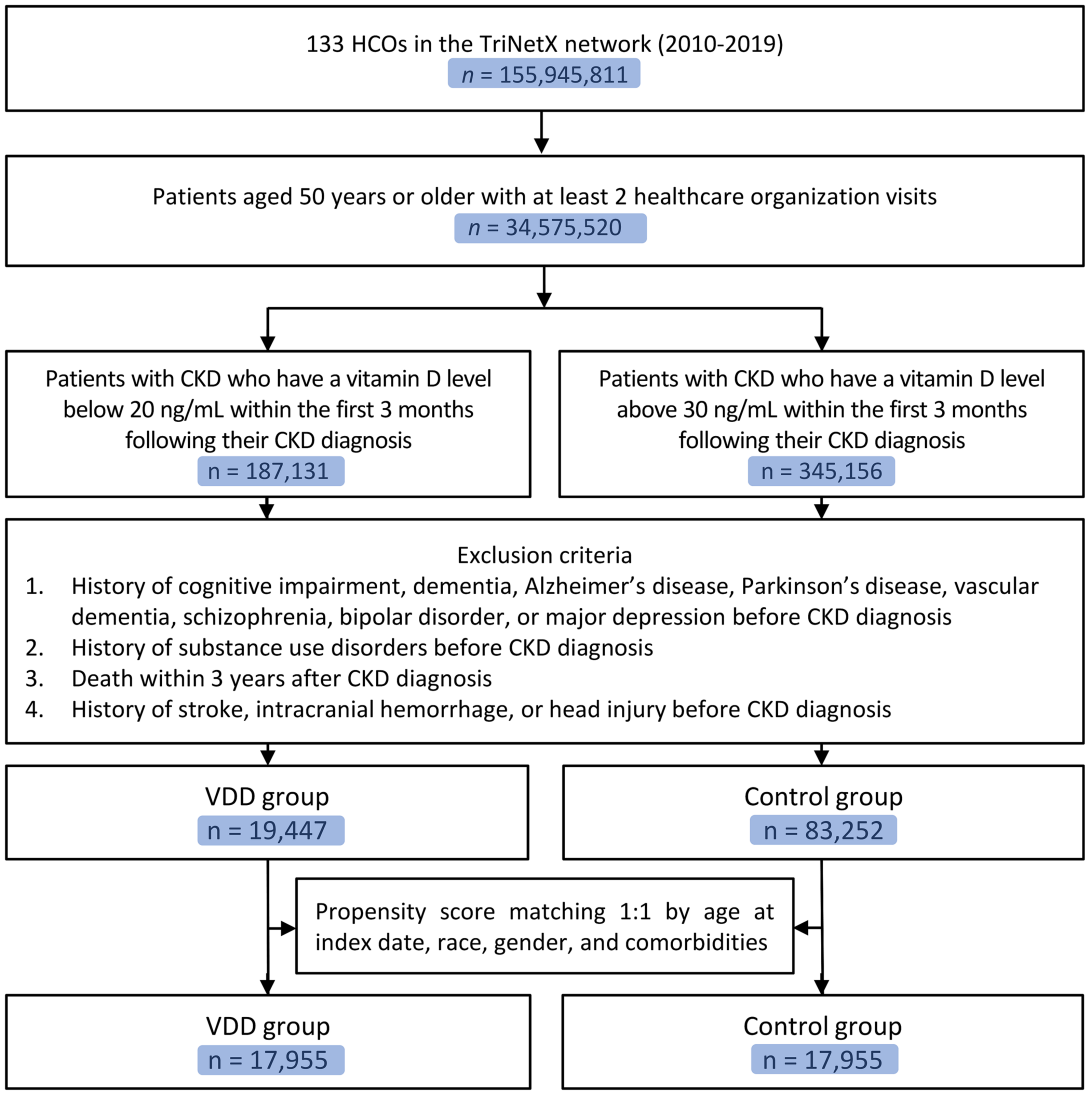
Flow diagram of patient selection. CKD, chronic kidney disease; VDD, vitamin D deficiency; HCOS, healthcare organizations.

From a total of 155,945,811 patients in 133 healthcare organizations within the TriNetX network, we identified 34,575,520 patients aged ≥50 years with at least two healthcare organization visits (Figure 1). Among these, we identified patients with CKD who had vitamin D measurements within the first 3 months following their CKD diagnosis. There were 187,131 patients with vitamin D levels below 20 ng/mL (VDD group) and 345,156 patients with vitamin D levels above 30 ng/mL (control group). After applying the exclusion criteria, the VDD group included 19,447 patients, whereas the control group comprised 89,252 patients. Following propensity score matching with a 1:1 ratio, the final analysis included 17,955 matched pairs of patients in the VDD and control groups.

### 3.2 Characteristics of patients before and after matching

Prior to propensity score matching, significant differences were observed between the VDD (*n* = 19, 447) and control (*n* = 89, 252) groups (Table 1). The VDD group was younger (65.8 ± 10.5 vs. 71.3 ± 19.0 years), had a higher BMI (31.5 ± 7.9 vs. 29.8 ± 6.7), and a higher proportion of males (52.1 vs. 43.1%). The VDD group also had a lower proportion of White patients (44.6 vs. 68.7%) and a higher proportion of Black or African American patients (25.3 vs. 12.6%). The distribution of CKD stages before matching showed significant differences. Stage 3 CKD was most prevalent in both groups, but with different proportions (42.3 vs. 58.6%), followed by end-stage renal disease (16.1 vs. 4.8%) and stage 4 CKD (14.3 vs. 8.7%) in the VDD and control groups, respectively. Stage 1 and 2 CKD were less common in both groups.

**Table 1:** Characteristics of patients with chronic kidney disease before and after matching.

Variables	Before matching			After matching		
	VDD group *N* = 19,447	Control group *N* = 83,252	Std. diff	VDD group *N* = 17,955	Control group *N* = 17,955	Std. diff
Patient characteristics						
Age at Index	65.8 ± 10.5	71.3 ± 9.0	0.568	66.5 ± 10.3	66.7 ± 9.3	0.016
Body mass index (kg/m^2^)	31.5 ± 7.9	29.8 ± 6.7	0.232	31.5 ± 7.9	30.8 ± 7.3	0.008
Male	10,140 (52.1%)	35,919 (43.1%)	0.181	9,137 (50.9%)	9,159 (51.0%)	0.002
White	8,678 (44.6%)	57,229 (68.7%)	0.502	8,511 (47.4%)	8,428 (46.9%)	0.009
Black or African American	4,927 (25.3%)	10,479 (12.6%)	0.330	4,282 (23.8%)	4,405 (24.5%)	0.016
unknown race	3,712 (19.1%)	8,438 (10.1%)	0.256	3,227 (18.0%)	3,228 (18.0%)	<0.001
Factors influencing health status and contact with health services	14,415 (74.1%)	63,538 (76.3%)	0.051	13,274 (73.9%)	13,225 (73.7%)	0.006
Comorbidities						
Essential (primary) hypertension	12,668 (65.1%)	60,294 (72.4%)	0.158	11,832 (65.9%)	11,876 (66.1%)	0.005
Diabetes mellitus	10,061 (51.7%)	31,485 (37.8%)	0.283	9,009 (50.2%)	8,967 (49.9%)	0.005
Overweight and obesity	5,206 (26.8%)	19,405 (23.3%)	0.080	4,750 (26.5%)	4,772 (26.6%)	0.003
Ischemic heart diseases	5,282 (27.2%)	19,116 (23.0%)	0.097	4,737 (26.4%)	4,724 (26.3%)	0.002
Neoplasms	4,655 (23.9%)	28,670 (34.4%)	0.233	4,494 (25.0%)	4,474 (24.9%)	0.003
Liver diseases	1,380 (7.1%)	5,416 (6.5%)	0.023	1,264 (7.0%)	1,191 (6.6%)	0.016
Cerebrovascular diseases	1,295 (6.7%)	5,047 (6.1%)	0.024	1,174 (6.5%)	1,100 (6.1%)	0.017
Malnutrition	1,064 (5.5%)	1858 (2.2%)	0.169	841 (4.7%)	884 (4.9%)	0.011
Hyperparathyroidism	771 (4.0%)	4,053 (4.9%)	0.044	703 (3.9%)	641 (3.6%)	0.018
Chronic kidney disease, stage 1	376 (1.9%)	1841 (2.2%)	0.020	366 (2.0%)	356 (2.0%)	0.004
Chronic kidney disease, stage 2	1708 (8.8%)	9,939 (11.9%)	0.104	1,681 (9.4%)	1,643 (9.2%)	0.007
Chronic kidney disease, stage 3	8,222 (42.3%)	48,775 (58.6%)	0.331	7,964 (44.4%)	7,947 (44.3%)	0.002
Chronic kidney disease, stage 4	2,781 (14.3%)	7,278 (8.7%)	0.175	2,412 (13.4%)	2,466 (13.7%)	0.009
Chronic kidney disease, stage 5	1,273 (6.5%)	1,664 (2.0%)	0.226	974 (5.4%)	994 (5.5%)	0.005
End stage renal disease	3,137 (16.1%)	4,032 (4.8%)	0.375	2,397 (13.4%)	2,446 (13.6%)	0.008
Laboratory data						
Hemoglobin (g/dL)	11.2 ± 2.5	12.7 ± 2.0	0.626	11.3 ± 2.5	12.2 ± 2.2	0.001
Albumin (g/dL)	3.6 ± 0.7	4.0 ± 0.5	0.692	3.6 ± 0.7	3.9 ± 0.6	0.008
Glomerular filtration rate (mL/min/1.73m^2^)	37.6 ± 23.7	46.5 ± 17.6	0.430	38.7 ± 23.4	42.9 ± 21.1	0.006

VDD, vitamin D deficiency; std diff, standardized difference.

After performing 1:1 propensity score matching, resulting in 17,955 participants in each group, the baseline characteristics of the two groups were successfully balanced (Table 1). The matched cohorts had similar demographics and CKD stage distributions. Stage 3 CKD remained the most common (44.4 vs. 44.3%), followed by stage 4 (13.4 vs. 13.7%). The proportions of other CKD stages, including end-stage renal disease (13.4 vs. 13.6%), were comparable between the groups. Laboratory parameters showed similar levels of hemoglobin, albumin, and glomerular filtration rate between the matched VDD and control groups.

### 3.3 Outcomes

**Figure 2:**
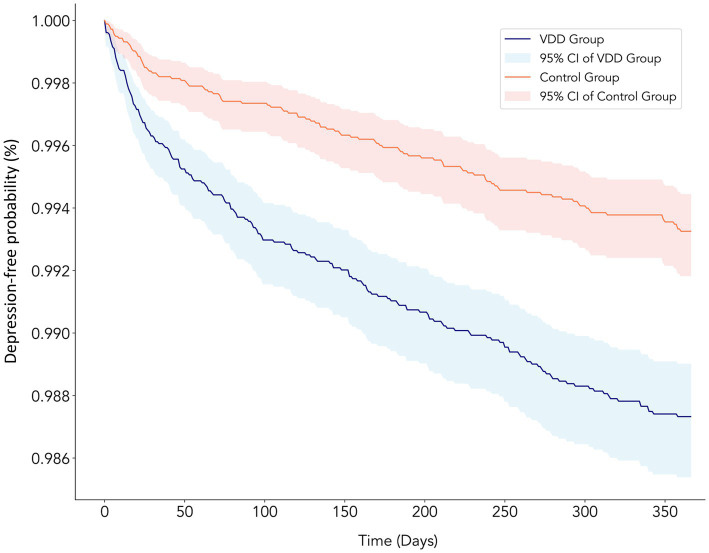
Kaplan–Meier curves showing the depression-free probability over 1 year of follow-up in chronic kidney disease patients with vitamin D deficiency (VDD) vs. control groups. The blue line represents the VDD group (≤20 ng/mL) and the red line represents the control group (≥30 ng/mL), with corresponding shaded areas indicating 95% confidence intervals. The VDD group showed a significantly higher risk of developing depression than the control group (HR: 1.929; 95% CI: 1.52–2.448; *p* < 0.0001), with the curves diverging early and the difference persisting throughout the follow-up period. The y-axis shows the probability of remaining depression-free, while the x-axis shows the time in days from the baseline.

During the 3-year follow-up period, we observed a consistently higher incidence of major depression in the VDD group than in the control group (Table 2). For the primary outcome at the 1-year follow-up, 191 patients (1.06%) in the VDD group developed major depression compared to 105 patients (0.59%) in the control group (HR: 1.929; 95% CI: 1.52–2.448; *p* < 0.0001) (Figure 2). The cumulative incidence of depression continued to increase in both groups over time. At the 2-year follow-up, depression occurred in 1.38 vs. 0.86% of patients (HR: 1.715; 95% CI: 1.403–2.097; *p* < 0.0001), and at the 3-year follow-up in 1.60 vs. 1.08% of patients (HR: 1.603; 95% CI: 1.337–1.924; p < 0.0001) in the VDD and control groups, respectively (Table 2). While the hazard ratios showed a gradual decline over time, the association between VDD and an increased risk of depression remained statistically significant throughout the follow-up period.

**Table 2:** Incidence and risk of major depression in patients with chronic kidney disease at a 3-year follow-up: comparison between patients with vitamin D deficiency and those without.

Follow-up time	VDD group (*n* = 17,955) Event (%)	Control group (*n* = 17,955) Event (%)	HR (95%)	Log-rank test
1-year	191 (1.06%)	105 (0.59%)	1.929 (1.52, 2.448)	< 0.0001
2-year	247 (1.38%)	155 (0.86%)	1.715 (1.403, 2.097)	< 0.0001
3-year	288 (1.60%)	194 (1.08%)	1.603 (1.337, 1.924)	< 0.0001
3 month–1 year	103 (0.57%)	68 (0.38%)	1.646 (1.212,2.236)	0.0013
3 month–2 year	162 (0.90%)	120 (0.67%)	1.484 (1.172,1.879)	0.001
3 month–3 year	205 (1.15%)	174 (0.98%)	1.291 (1.055,1.58)	0.013

VDD, vitamin D deficiency; HR, hazard ratio.

### 3.4 Subgroup analyses based on sex and severity of CKD

In sex-stratified analyses, VDD was associated with an increased risk of depression in both males and females, with males showing a notably higher risk (Table 3). Among male patients (*n* = 9,119), the VDD group had a 2-fold higher risk of depression than the control group (HR: 2.264; 95% CI: 1.498–3.421; *p* < 0.0001). Female patients (*n* = 8,197) also showed an elevated risk in the VDD group (HR: 1.761; 95% CI: 1.307–2.374; *p* = 0.0002). When stratified by CKD severity, VDD was consistently associated with an increased risk of depression across all stages. Patients with early stage CKD (stages 1–2, *n* = 3,257) showed a higher baseline event rate but similar relative risk (HR: 1.977; 95% CI: 1.382–2.829; *p* = 0.0001), with depression occurring in 2.6% vs. 1.4% of the patients. In CKD stage 3–5 (*n* = 14,460), the risk remained significantly elevated (HR: 1.981; 95% CI: 1.533–2.559; *p* < 0.0001). These findings suggest that the association between VDD and depression risk is consistent across both sex and CKD severity subgroups, with males potentially showing greater susceptibility.

**Table 3:** Subgroup analyses based on sex and severity of CKD.

Subgroup	VDD group Event (%)	Control group Event (%)	HR (95%)	Log-rank test
Female (*n* = 8,197)	115 (1.4%)	69 (0.8%)	1.761 (1.307, 2.374)	0.0002
Male (*n* = 9,119)	71 (0.7%)	33 (0.3%)	2.264 (1.498, 3.421)	< 0.0001
CKD stage 1–2 (*n* = 3,257)	86 (2.6%)	46 (1.4%)	1.977 (1.382, 2.829)	0.0001
CKD stage 3–5 (*n* = 14,460)	167 (1.1%)	90 (0.6%)	1.981 (1.533, 2.559)	< 0.0001

CKD, chronic kidney disease.

### 3.5 Dose-dependent effect of vitamin D on depression

To investigate the dose-dependent relationship between vitamin D levels and depression risk, we conducted an additional analysis comparing patients with vitamin D insufficiency (20–30 ng/mL, *n* = 18,939) to those with normal vitamin D levels (≥30 ng/mL, *n* = 18,939). The analysis revealed that even patients with vitamin D insufficiency had a significantly higher risk of developing depression than the control group (HR: 1.667; 95% CI: 1.318–2.11; *p* < 0.0001). The event rates were 0.95% in the vitamin D-insufficient group and 0.6% in the control group.

### 3.6 Risk factors for depression in patients with vitamin D deficiency

Logistic regression analysis identified several significant risk factors for depression in patients with concurrent CKD and VDD (Table 4). In the multivariable analysis, cerebrovascular disease (adjusted OR: 2.701; 95% CI: 1.822–4.002; *p* < 0.00001) and malnutrition (adjusted OR: 2.004; 95% CI: 1.277–3.146; *p* = 0.002) emerged as the strongest independent risk factors, with more than twice the odds of developing depression. Ischemic heart disease also showed a significant association with increased depression risk (adjusted OR: 1.429; 95% CI: 1.025–1.991; *p* = 0.035). Other comorbidities were not significantly associated with depression risk in the adjusted analysis (all *p* > 0.05).

**Table 4:** Risk factors for major depression in patients with chronic kidney disease.

Covariate	Crude OR (95% CI)	*P*-value	Adjusted OR (95% CI)	*P-*value
Essential (primary) hypertension	0.879 (0.66, 1.171)	0.379	0.977 (0.714, 1.336)	0.884
Neoplasms	0.997 (0.72, 1.381)	0.986	0.937 (0.668, 1.315)	0.708
Overweight and obesity	1.166 (0.861, 1.58)	0.321	1.068 (0.765, 1.491)	0.698
Diabetes mellitus	0.801 (0.606, 1.059)	0.119	0.739 (0.545, 1.003)	0.052
Ischemic heart diseases	1.171 (0.865, 1.584)	0.306	1.429 (1.025, 1.991)	0.035
Cerebrovascular diseases	2.494 (1.685, 3.692)	<0.00001	2.701 (1.822, 4.002)	<0.00001
COPD	1.475 (0.926, 2.348)	0.101	1.527 (0.962, 2.423)	0.072
Malnutrition	2.374 (1.543, 3.653)	0	2.004 (1.277, 3.146)	0.002
Factors influencing health status and contact with health services	1.116 (0.806, 1.547)	0.508	0.994 (0.704, 1.402)	0.971
Hyperparathyroidism	0.742 (0.328, 1.678)	0.473	0.851 (0.364, 1.988)	0.71
Liver diseases	1.374 (0.854, 2.21)	0.19	1.045 (0.637, 1.715)	0.862

COPD, Chronic obstructive pulmonary disease; OR, Odds ratio; CI: confidence interval.

## 4 Discussion

Among 17,955 matched pairs of patients with CKD, those with VDD showed a significantly higher risk of developing major depression than those with normal vitamin D levels. At one-year follow-up, depression occurred in 1.06% of VDD patients vs. 0.59% of controls (HR: 1.929; 95% CI: 1.52–2.448; *p* < 0.0001). This association remained significant through 3 years of follow-up. Subgroup analyses revealed that males with VDD had a notably higher depression risk (HR: 2.264) than females (HR: 1.761). This association was consistent across all CKD stages. Even patients with vitamin D insufficiency (20–30 ng/mL) showed an elevated depression risk compared to those with normal levels (HR: 1.667). In patients with both CKD and VDD, cerebrovascular disease (adjusted OR 2.701) and malnutrition (adjusted OR 2.004) emerged as the strongest independent risk factors for depression.

Our findings demonstrate a robust association between VDD and an increased risk of major depression in CKD patients, consistent with a previous meta-analysis in the general population that linked low vitamin D levels to depression (19). However, the strength and nature of this association may differ among patient populations. A recent meta-analysis studying stroke patients found that VDD in the acute phase was associated with an increased risk of post-stroke depression (OR: 3.59) (23), suggesting an even stronger association than observed in our CKD cohort (HR: 1.929). Interestingly, while the study (23) found no significant association between vitamin D insufficiency and depression, our study demonstrated that even insufficient vitamin D levels increased depression risk in patients with CKD. This difference suggests that the relationship between vitamin D and depression may be disease-specific and context-dependent. These findings have several important clinical implications. First, they suggested that vitamin D screening and monitoring should be considered as part of comprehensive depression prevention strategies in CKD patients, although optimal target levels may differ from other populations. Second, the identification of risk factors (i.e., cerebrovascular disease and malnutrition) may help clinicians target preventive interventions more effectively.

The definition of VDD and appropriate threshold values warrant careful consideration. In our study, we used the widely accepted cutoff of <20 ng/mL (i.e., 50 nmol/L) for VDD, aligning with guidelines established by the Endocrine Society Task Force on Vitamin D (24). However, the optimal vitamin D levels may vary across different populations and ethnicities (25). For instance, individuals with darker skin typically require greater sun exposure to produce the same amount of vitamin D as those with lighter skin, and genetic variations in vitamin D binding protein can affect bioavailable vitamin D levels (26). Our study population included diverse ethnic groups, but we applied uniform thresholds across all ethnicities. This approach, while consistent with current clinical practice guidelines, may not fully account for ethnic-specific vitamin D requirements. Therefore, our findings should be interpreted with consideration of these ethnic variations in vitamin D metabolism and the ongoing debate about population-specific threshold values for defining VDD.

Our sex-stratified analysis revealed a notably stronger association between VDD and depression risk in male CKD patients (HR: 2.264) than in females (HR: 1.761). This finding aligns partly with a recent study of hemodialysis patients that found that VDD was independently associated with depression only in males (OR: 8.207), while this association was not significant in females (27). Similarly, a cross-sectional study of 612 patients with CKD found that male sex itself was an independent risk factor for depression (OR 1.3), regardless of vitamin D status (28). The stronger association between VDD and depression in males suggests that vitamin D might be a particularly important modifiable risk factor in this population. Several reasons might explain this stronger association in males. First, males with CKD might be less likely to seek mental health care or report depressive symptoms, leading to delayed diagnosis and potentially more severe cases when finally detected. Second, hormonal interactions between vitamin D and sex hormones could differ in the context of CKD (29), possibly affecting mood regulation differently in males and females.

Our study utilized data from 133 healthcare organizations within the TriNetX network, representing diverse patient populations across different geographical locations and healthcare settings. This broad sampling enhances the external validity of our findings compared to single-center studies. However, our study population consisted of patients aged 50 years or older who had documented vitamin D measurements within 3 months after CKD diagnosis, suggesting that they may seek medical attention more aggressively and have more regular healthcare monitoring. The relatively low incidence of depression observed in our study (1.06% in the VDD group vs. 0.59% in the control group at 1 year) further indicates that our patient population might not represent CKD patients with limited healthcare access or those who seek medical attention only for acute problems. This selection bias might have led to an underestimation of the true association between VDD and depression in the broader CKD population. Additionally, the availability and implementation of mental health services can differ significantly across healthcare settings and geographical regions. While our matched cohorts included patients across all CKD stages and diverse racial/ethnic backgrounds, the effectiveness of our findings may vary in different healthcare systems with varying screening practices, diagnostic criteria for depression, and treatment protocols.

The association between VDD and depression risk in our study suggests potential therapeutic implications for vitamin D supplementation. While supplementation could theoretically help prevent depression in CKD patients, several key questions remain unanswered. The optimal type of supplementation (nutritional vitamin D vs. activated analogs), dosing regimens, and target levels specific to CKD populations are unknown. Additionally, the timing of intervention may be crucial - early supplementation might prevent depression, but its effectiveness in treating established depression is uncertain. Randomized controlled trials are needed to determine whether vitamin D supplementation could serve as a preventive strategy for depression in CKD patients and to establish evidence-based supplementation protocols.

An important methodological consideration in interpreting our findings is the potential variability in vitamin D measurement techniques across the participating healthcare organizations. While liquid chromatography-mass spectrometry (LC–MS/MS) is considered the gold standard for measuring vitamin D metabolites due to its superior sensitivity and specificity, various other assay methods are commonly used in clinical practice, including radioimmunoassay, enzyme immunoassay, and chemiluminescence immunoassay (30–33). These different methodologies can yield varying results for the same sample, with inter-method variability potentially affecting vitamin D level classifications. In our study using the TriNetX network database, which aggregates data from multiple institutions, we could not standardize or verify the specific assay methods used at each site. This analytical variability could have influenced our categorical classification of vitamin D status and potentially impacted the observed associations. However, the robust and consistent relationship between VDD and depression risk across different subgroups and the observed dose-dependent effect suggest that our main findings are unlikely to be solely attributable to assay variability.

Several significant limitations of this study should be considered when interpreting the results. First, as this was an observational study using electronic health records, we could not establish causality between VDD and depression. While we found a significant association, the relationship could be bidirectional. Depression may lead to VDD through behavioral changes including reduced outdoor activities, poor dietary habits, and decreased adherence to vitamin D supplementation. Moreover, depressive symptoms such as fatigue and social withdrawal might result in less sun exposure, further contributing to VDD. These unmeasured confounders could have influenced our findings, despite our attempts to control for multiple clinical variables through propensity score matching. Second, depression diagnosis in our study relied on clinician documentation and diagnostic codes, which may not capture the full spectrum of depressive symptoms. This approach could miss cases where patients experienced depressive symptoms but did not seek mental health care, potentially leading to an underestimation of the true association. Furthermore, cultural and socioeconomic factors affecting healthcare-seeking behaviors for mental health issues could have influenced depression diagnosis rates. Third, although we required vitamin D measurements within 3 months of CKD diagnosis, we could not account for potential changes in vitamin D levels during the follow-up period. The dynamic nature of both vitamin D status and kidney function over time may have influenced our findings. Fourth, information on vitamin D supplementation, adherence to treatment, and other medications that might affect both vitamin D metabolism and risk of depression was not consistently available in the database. Fifth, seasonal variations in vitamin D levels (34) and their potential impact on mood disorders could not be adequately assessed, owing to limitations in the temporal resolution of our data. The timing of vitamin D measurements relative to seasonal changes might have affected our results. Finally, our study did not analyze several important clinical factors that could influence depression risk, including symptom burden (e.g., dyspnea from fluid overload), frequency of follow-up visits, and complications requiring frequent monitoring such as hyperkalemia. These factors could significantly impact patients’ quality of life and mental health outcomes.

## 5 Conclusion

In this large-scale cohort study, VDD was significantly associated with an increased risk of major depression in CKD patients, with the effect persisting throughout the 3 years of follow-up. The association remained robust across different subgroups, with male patients showing particular vulnerability. The observed dose-dependent relationship, where even vitamin D insufficiency carried an elevated depression risk, suggests that maintaining adequate vitamin D levels may be crucial for mental health in patients with CKD. The identification of cerebrovascular disease and malnutrition as strong independent risk factors for depression in patients with vitamin D-deficient CKD highlights the complex interplay between physical and mental health in this population. These findings suggest that routine vitamin D screening and appropriate supplementation might be valuable strategies for depression prevention in CKD patients. Further randomized controlled trials are needed to determine whether vitamin D supplementation can effectively prevent or reduce risk of depression in this vulnerable population. Clinicians should remain vigilant for depression symptoms, particularly in CKD patients with VDD and additional risk factors.

## Data Availability

The datasets presented in this article are not readily available because the data that support the findings of this study are available from TriNetX Research Network, but restrictions apply to the availability of these data, which were used under a collaboration agreement for the current study and so are not publicly available. Requests to access the datasets should be directed to https://live.trinetx.com.
