# Supplementary material for: Association between vitamin D deficiency and major depression in patients with chronic kidney disease: a cohort study
Source: Front Nutr. 2025 Jan 27;12:1540633. doi: 10.3389/fnut.2025.1540633 (PMC11807813; doi:10.3389/fnut.2025.1540633)
Supplement: Supplementary file 1 [file Table_1.docx]

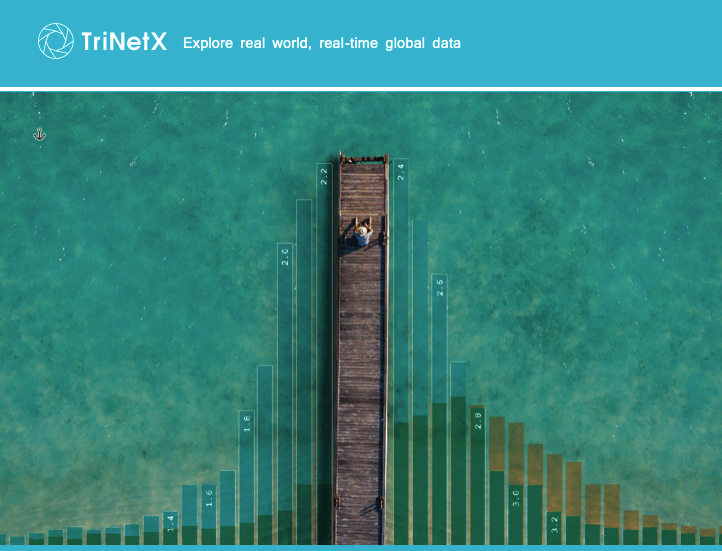


(20241110) vitamin D可否預測CKD病人 depression

Compare Outcomes Analysis

Created by TriNetX on Nov 10, 2024, 09:20:51 UTC

# Introduction

TriNetX is the global federated health research network providing access to electronic medical records (diagnoses, procedures, medications, laboratory values, genomic information) across large healthcare organizations (HCOs). This report was run on the set of HCOs grouped into a network called Global Collaborative Network. This network included 133 HCO(s).

This report describes a Compare Outcomes Analysis, named #v2 depression (VDD vs. control) (>50 years) (new) (1), generated by the TriNetX platform on Nov 10, 2024, 09:20:51 UTC. This analysis compared the outcomes of two cohorts: Cohort A (19,797 patients) named ###VDD v2 (>50, depression and Cohort B (83,731 patients) named ###control V2 (>50, depression).

This analysis was run by Kuo-Chuan Hung (ed102605@gmail.com) and downloaded by Kuo-Chuan Hung (ed102605@gmail.com).

# Methods

The analysis process includes two main steps: 1) Defining the cohorts through query criteria; 2) Setting up and running the analysis. Setting up the analysis requires definitions for the index event, outcomes criteria, and the time frame. Compare outcomes supports four analyses: Measures of Association, Survival, Number of Instances and Lab result distribution. These analyses have additional options that are listed in the Outcomes Definitions and Analyses Specifications section below. Furthermore, characteristics of the cohorts that are balanced using propensity score matching are also included in the Propensity Score Matching section.

## Cohorts definition

This section lists all terms used in the definitions of the two cohorts.

### Query Criteria for Cohort 1 (query name: ###VDD v2 (>50, depression)

This query was run on the network Global Collaborative Network with 133 HCO(s) queried and 133 HCO(s) responded. A total of 76 provider(s) responded with patients. The final cohort included 19,797 patients who matched the query criteria listed in the table below. For the text representation of the query criteria please see Appendix A.

|  | | | | | |
| --- | --- | --- | --- | --- | --- |
| Ungrouped terms | | | | | |
|  | must have |  | demographics | Age | Age (at least 50 years (most recent occurrence)) |
| Group 1 | | | | | |
|  | **Visit>2** | | | | |
|  | must have |  | visit | TNX:Visit | Visit |
|  | number of instances | | Greater than or equal to 2 instances | | |
|  | date constraint | | The terms in this group occurred between Jan 1, 2010 and Dec 31, 2019 | | |
| Group 2 | | | | | |
|  | **Group 2A CKD history** | | | | |
|  | must have | any of | diagnosis | UMLS:ICD10CM:N18 | Chronic kidney disease (CKD) (at least 18 years old at event) |
|  |  |  | diagnosis | UMLS:ICD10CM:N18.9 | Chronic kidney disease, unspecified (at least 18 years old at event) |
|  |  |  | diagnosis | UMLS:ICD10CM:N18.3 | Chronic kidney disease, stage 3 (moderate) (at least 18 years old at event) |
|  |  |  | diagnosis | UMLS:ICD10CM:N18.30 | Chronic kidney disease, stage 3 unspecified (at least 18 years old at event) |
|  |  |  | diagnosis | UMLS:ICD10CM:N18.4 | Chronic kidney disease, stage 4 (severe) (at least 18 years old at event) |
|  |  |  | diagnosis | UMLS:ICD10CM:N18.2 | Chronic kidney disease, stage 2 (mild) (at least 18 years old at event) |
|  |  |  | diagnosis | UMLS:ICD10CM:N18.31 | Chronic kidney disease, stage 3a (at least 18 years old at event) |
|  |  |  | diagnosis | UMLS:ICD10CM:N18.32 | Chronic kidney disease, stage 3b (at least 18 years old at event) |
|  |  |  | diagnosis | UMLS:ICD10CM:N18.5 | Chronic kidney disease, stage 5 (at least 18 years old at event) |
|  |  |  | diagnosis | UMLS:ICD10CM:N18.1 | Chronic kidney disease, stage 1 (at least 18 years old at event) |
|  |  |  | diagnosis | UMLS:ICD10CM:N18.6 | End stage renal disease (at least 18 years old at event) |
|  | date constraint | | The terms in this group occurred at any time | | |
|  | event relationship | | Any instance of CKD三個月內vitamin D<20 occurred within 3 months on or after any instance of CKD history | | |
|  | **Group 2B CKD三個月內vitamin D<20** | | | | |
|  | must have | any of | laboratory | UMLS:LNC:35365-6 | Vitamin D+Metabolites [Mass/volume] in Serum or Plasma (at most 20.00 ng/mL) |
|  |  |  | laboratory | TNX:LG25965-1 | Calcidiol+ercalcidiol [Mass/volume] in Serum, Plasma or Blood (at most 20.00 ng/mL) |
|  |  |  | laboratory | TNX:9034 | Calcidiol [Mass/volume] in Serum or Plasma (at most 20.00 ng/mL) |
| Group 3 | | | | | |
|  | **Group 3A CKD history** | | | | |
|  | must have | any of | diagnosis | UMLS:ICD10CM:N18 | Chronic kidney disease (CKD) (at least 50 years old at event) |
|  |  |  | diagnosis | UMLS:ICD10CM:N18.9 | Chronic kidney disease, unspecified (at least 50 years old at event) |
|  |  |  | diagnosis | UMLS:ICD10CM:N18.3 | Chronic kidney disease, stage 3 (moderate) (at least 50 years old at event) |
|  |  |  | diagnosis | UMLS:ICD10CM:N18.30 | Chronic kidney disease, stage 3 unspecified (at least 50 years old at event) |
|  |  |  | diagnosis | UMLS:ICD10CM:N18.4 | Chronic kidney disease, stage 4 (severe) (at least 50 years old at event) |
|  |  |  | diagnosis | UMLS:ICD10CM:N18.2 | Chronic kidney disease, stage 2 (mild) (at least 50 years old at event) |
|  |  |  | diagnosis | UMLS:ICD10CM:N18.31 | Chronic kidney disease, stage 3a (at least 50 years old at event) |
|  |  |  | diagnosis | UMLS:ICD10CM:N18.32 | Chronic kidney disease, stage 3b (at least 50 years old at event) |
|  |  |  | diagnosis | UMLS:ICD10CM:N18.5 | Chronic kidney disease, stage 5 (at least 50 years old at event) |
|  |  |  | diagnosis | UMLS:ICD10CM:N18.1 | Chronic kidney disease, stage 1 (at least 50 years old at event) |
|  |  |  | diagnosis | UMLS:ICD10CM:N18.6 | End stage renal disease (at least 50 years old at event) |
|  | date constraint | | The terms in this group occurred at any time | | |
|  | event relationship | | Any instance of 在CKD三個月內排除vitamin D>21 occurred within 3 months on or after any instance of CKD history | | |
|  | **Group 3B 在CKD三個月內排除vitamin D>21** | | | | |
|  | cannot have |  | laboratory | TNX:LG25965-1 | Calcidiol+ercalcidiol [Mass/volume] in Serum, Plasma or Blood (at least 21.00 ng/mL) |
|  |  | or | laboratory | TNX:9034 | Calcidiol [Mass/volume] in Serum or Plasma (at least 21.00 ng/mL) |
|  |  | or | laboratory | UMLS:LNC:35365-6 | Vitamin D+Metabolites [Mass/volume] in Serum or Plasma (at least 21.00 ng/mL) |
| Group 4 | | | | | |
|  | **Group 4A CKD history** | | | | |
|  | must have | any of | diagnosis | UMLS:ICD10CM:N18 | Chronic kidney disease (CKD) (at least 18 years old at event) |
|  |  |  | diagnosis | UMLS:ICD10CM:N18.9 | Chronic kidney disease, unspecified (at least 18 years old at event) |
|  |  |  | diagnosis | UMLS:ICD10CM:N18.3 | Chronic kidney disease, stage 3 (moderate) (at least 18 years old at event) |
|  |  |  | diagnosis | UMLS:ICD10CM:N18.30 | Chronic kidney disease, stage 3 unspecified (at least 18 years old at event) |
|  |  |  | diagnosis | UMLS:ICD10CM:N18.4 | Chronic kidney disease, stage 4 (severe) (at least 18 years old at event) |
|  |  |  | diagnosis | UMLS:ICD10CM:N18.2 | Chronic kidney disease, stage 2 (mild) (at least 18 years old at event) |
|  |  |  | diagnosis | UMLS:ICD10CM:N18.31 | Chronic kidney disease, stage 3a (at least 18 years old at event) |
|  |  |  | diagnosis | UMLS:ICD10CM:N18.32 | Chronic kidney disease, stage 3b (at least 18 years old at event) |
|  |  |  | diagnosis | UMLS:ICD10CM:N18.5 | Chronic kidney disease, stage 5 (at least 18 years old at event) |
|  |  |  | diagnosis | UMLS:ICD10CM:N18.1 | Chronic kidney disease, stage 1 (at least 18 years old at event) |
|  |  |  | diagnosis | UMLS:ICD10CM:N18.6 | End stage renal disease (at least 18 years old at event) |
|  | date constraint | | The terms in this group occurred at any time | | |
|  | event relationship | | Any instance of 排除cognitive impairment occurred at least 1 day before any instance of CKD history | | |
|  | **Group 4B 排除cognitive impairment** | | | | |
|  | cannot have |  | diagnosis | UMLS:ICD10CM:B20 | Human immunodeficiency virus [HIV] disease |
|  |  | or | diagnosis | UMLS:ICD10CM:G31.84 | Mild cognitive impairment of uncertain or unknown etiology |
|  |  | or | diagnosis | UMLS:ICD10CM:F06 | Other mental disorders due to known physiological condition |
|  |  | or | diagnosis | UMLS:ICD10CM:F06.8 | Other specified mental disorders due to known physiological condition |
|  |  | or | diagnosis | UMLS:ICD10CM:F88 | Other disorders of psychological development |
|  |  | or | procedure | UMLS:ICD10PCS:GZ52ZZZ | Individual Psychotherapy, Cognitive |
|  |  | or | diagnosis | UMLS:ICD10CM:F06.7 | Mild neurocognitive disorder due to known physiological condition |
|  |  | or | diagnosis | UMLS:ICD10CM:F09 | Unspecified mental disorder due to known physiological condition |
| Group 5 | | | | | |
|  | **Group 5A CKD history** | | | | |
|  | must have | any of | diagnosis | UMLS:ICD10CM:N18 | Chronic kidney disease (CKD) (at least 18 years old at event) |
|  |  |  | diagnosis | UMLS:ICD10CM:N18.9 | Chronic kidney disease, unspecified (at least 18 years old at event) |
|  |  |  | diagnosis | UMLS:ICD10CM:N18.3 | Chronic kidney disease, stage 3 (moderate) (at least 18 years old at event) |
|  |  |  | diagnosis | UMLS:ICD10CM:N18.30 | Chronic kidney disease, stage 3 unspecified (at least 18 years old at event) |
|  |  |  | diagnosis | UMLS:ICD10CM:N18.4 | Chronic kidney disease, stage 4 (severe) (at least 18 years old at event) |
|  |  |  | diagnosis | UMLS:ICD10CM:N18.2 | Chronic kidney disease, stage 2 (mild) (at least 18 years old at event) |
|  |  |  | diagnosis | UMLS:ICD10CM:N18.31 | Chronic kidney disease, stage 3a (at least 18 years old at event) |
|  |  |  | diagnosis | UMLS:ICD10CM:N18.32 | Chronic kidney disease, stage 3b (at least 18 years old at event) |
|  |  |  | diagnosis | UMLS:ICD10CM:N18.5 | Chronic kidney disease, stage 5 (at least 18 years old at event) |
|  |  |  | diagnosis | UMLS:ICD10CM:N18.1 | Chronic kidney disease, stage 1 (at least 18 years old at event) |
|  |  |  | diagnosis | UMLS:ICD10CM:N18.6 | End stage renal disease (at least 18 years old at event) |
|  | date constraint | | The terms in this group occurred at any time | | |
|  | event relationship | | Any instance of 排除dementia，Alzheimer's disease, Parkinsom disease occurred at least 1 day before any instance of CKD history | | |
|  | **Group 5B 排除dementia，Alzheimer's disease, Parkinsom disease** | | | | |
|  | cannot have |  | diagnosis | UMLS:ICD10CM:F03 | Unspecified dementia |
|  |  | or | diagnosis | UMLS:ICD10CM:F03.90 | Unspecified dementia, unspecified severity, without behavioral disturbance, psychotic disturbance, mood disturbance, and anxiety |
|  |  | or | diagnosis | UMLS:ICD10CM:F02.80 | Dementia in other diseases classified elsewhere, unspecified severity, without behavioral disturbance, psychotic disturbance, mood disturbance, and anxiety |
|  |  | or | diagnosis | UMLS:ICD10CM:F06.0 | Psychotic disorder with hallucinations due to known physiological condition |
|  |  | or | diagnosis | UMLS:ICD10CM:G30 | Alzheimer's disease |
|  |  | or | diagnosis | UMLS:ICD10CM:G20 | Parkinson's disease |
|  |  | or | diagnosis | UMLS:ICD10CM:F05 | Delirium due to known physiological condition |
|  |  | or | diagnosis | UMLS:ICD10CM:G30 | Alzheimer's disease |
|  |  | or | diagnosis | UMLS:ICD10CM:F02.80 | Dementia in other diseases classified elsewhere, unspecified severity, without behavioral disturbance, psychotic disturbance, mood disturbance, and anxiety |
|  |  | or | diagnosis | UMLS:ICD10CM:G30.9 | Alzheimer's disease, unspecified |
|  |  | or | diagnosis | UMLS:ICD10CM:G30.8 | Other Alzheimer's disease |
|  |  | or | diagnosis | UMLS:ICD10CM:F05 | Delirium due to known physiological condition |
|  |  | or | diagnosis | UMLS:ICD10CM:F06.2 | Psychotic disorder with delusions due to known physiological condition |
|  |  | or | diagnosis | UMLS:ICD10CM:G30.1 | Alzheimer's disease with late onset |
|  |  | or | diagnosis | UMLS:ICD10CM:G30.0 | Alzheimer's disease with early onset |
|  |  | or | diagnosis | UMLS:ICD10CM:G30.1 | Alzheimer's disease with late onset |
|  |  | or | diagnosis | UMLS:ICD10CM:G30.8 | Other Alzheimer's disease |
|  |  | or | diagnosis | UMLS:ICD10CM:G30.9 | Alzheimer's disease, unspecified |
|  |  | or | diagnosis | UMLS:ICD10CM:F02.A | Dementia in other diseases classified elsewhere, mild |
|  |  | or | diagnosis | UMLS:ICD10CM:F02.C | Dementia in other diseases classified elsewhere, severe |
|  |  | or | diagnosis | UMLS:ICD10CM:F02.A0 | Dementia in other diseases classified elsewhere, mild, without behavioral disturbance, psychotic disturbance, mood disturbance, and anxiety |
|  |  | or | diagnosis | UMLS:ICD10CM:F02.B0 | Dementia in other diseases classified elsewhere, moderate, without behavioral disturbance, psychotic disturbance, mood disturbance, and anxiety |
|  |  | or | diagnosis | UMLS:ICD10CM:F02.83 | Dementia in other diseases classified elsewhere, unspecified severity, with mood disturbance |
|  |  | or | diagnosis | UMLS:ICD10CM:F02.811 | Dementia in other diseases classified elsewhere, unspecified severity, with agitation |
|  |  | or | diagnosis | UMLS:ICD10CM:F02.B | Dementia in other diseases classified elsewhere, moderate |
|  |  | or | diagnosis | UMLS:ICD10CM:F02.818 | Dementia in other diseases classified elsewhere, unspecified severity, with other behavioral disturbance |
|  |  | or | diagnosis | UMLS:ICD10CM:F02.81 | Dementia in other diseases classified elsewhere, unspecified severity, with behavioral disturbance |
|  |  | or | diagnosis | UMLS:ICD10CM:F03 | Unspecified dementia |
|  |  | or | diagnosis | UMLS:ICD10CM:F03.90 | Unspecified dementia, unspecified severity, without behavioral disturbance, psychotic disturbance, mood disturbance, and anxiety |
|  |  | or | diagnosis | UMLS:ICD10CM:F02.80 | Dementia in other diseases classified elsewhere, unspecified severity, without behavioral disturbance, psychotic disturbance, mood disturbance, and anxiety |
|  |  | or | diagnosis | UMLS:ICD10CM:F06.0 | Psychotic disorder with hallucinations due to known physiological condition |
|  |  | or | diagnosis | UMLS:ICD10CM:F02 | Dementia in other diseases classified elsewhere |
|  |  | or | diagnosis | UMLS:ICD10CM:F02.8 | Dementia in other diseases classified elsewhere, unspecified severity |
| Group 6 | | | | | |
|  | **排除renal transplanation** | | | | |
|  | cannot have |  | procedure | UMLS:CPT:1008098 | Renal Transplantation Procedures |
|  | date constraint | | The terms in this group occurred at any time | | |
| Group 7 | | | | | |
|  | **Group 7A CKD history** | | | | |
|  | must have | any of | diagnosis | UMLS:ICD10CM:N18 | Chronic kidney disease (CKD) (at least 18 years old at event) |
|  |  |  | diagnosis | UMLS:ICD10CM:N18.9 | Chronic kidney disease, unspecified (at least 18 years old at event) |
|  |  |  | diagnosis | UMLS:ICD10CM:N18.3 | Chronic kidney disease, stage 3 (moderate) (at least 18 years old at event) |
|  |  |  | diagnosis | UMLS:ICD10CM:N18.30 | Chronic kidney disease, stage 3 unspecified (at least 18 years old at event) |
|  |  |  | diagnosis | UMLS:ICD10CM:N18.4 | Chronic kidney disease, stage 4 (severe) (at least 18 years old at event) |
|  |  |  | diagnosis | UMLS:ICD10CM:N18.2 | Chronic kidney disease, stage 2 (mild) (at least 18 years old at event) |
|  |  |  | diagnosis | UMLS:ICD10CM:N18.31 | Chronic kidney disease, stage 3a (at least 18 years old at event) |
|  |  |  | diagnosis | UMLS:ICD10CM:N18.32 | Chronic kidney disease, stage 3b (at least 18 years old at event) |
|  |  |  | diagnosis | UMLS:ICD10CM:N18.5 | Chronic kidney disease, stage 5 (at least 18 years old at event) |
|  |  |  | diagnosis | UMLS:ICD10CM:N18.1 | Chronic kidney disease, stage 1 (at least 18 years old at event) |
|  |  |  | diagnosis | UMLS:ICD10CM:N18.6 | End stage renal disease (at least 18 years old at event) |
|  | date constraint | | The terms in this group occurred at any time | | |
|  | event relationship | | Any instance of 排除vascular dementia occurred at least 1 day before any instance of CKD history | | |
|  | **Group 7B 排除vascular dementia** | | | | |
|  | cannot have |  | diagnosis | UMLS:ICD10CM:F01 | Vascular dementia |
|  |  | or | diagnosis | UMLS:ICD10CM:I67.2 | Cerebral atherosclerosis |
|  |  | or | diagnosis | UMLS:ICD10CM:F01.50 | Vascular dementia, unspecified severity, without behavioral disturbance, psychotic disturbance, mood disturbance, and anxiety |
|  |  | or | diagnosis | UMLS:ICD10CM:F01.5 | Vascular dementia, unspecified severity |
|  |  | or | diagnosis | UMLS:ICD10CM:F01.A | Vascular dementia, mild |
|  |  | or | diagnosis | UMLS:ICD10CM:F01.B | Vascular dementia, moderate |
|  |  | or | diagnosis | UMLS:ICD10CM:F01.A0 | Vascular dementia, mild, without behavioral disturbance, psychotic disturbance, mood disturbance, and anxiety |
|  |  | or | diagnosis | UMLS:ICD10CM:F01.B0 | Vascular dementia, moderate, without behavioral disturbance, psychotic disturbance, mood disturbance, and anxiety |
|  |  | or | diagnosis | UMLS:ICD10CM:F01.C | Vascular dementia, severe |
| Group 8 | | | | | |
|  | **Group 8A CKD history** | | | | |
|  | must have | any of | diagnosis | UMLS:ICD10CM:N18 | Chronic kidney disease (CKD) (at least 18 years old at event) |
|  |  |  | diagnosis | UMLS:ICD10CM:N18.9 | Chronic kidney disease, unspecified (at least 18 years old at event) |
|  |  |  | diagnosis | UMLS:ICD10CM:N18.3 | Chronic kidney disease, stage 3 (moderate) (at least 18 years old at event) |
|  |  |  | diagnosis | UMLS:ICD10CM:N18.30 | Chronic kidney disease, stage 3 unspecified (at least 18 years old at event) |
|  |  |  | diagnosis | UMLS:ICD10CM:N18.4 | Chronic kidney disease, stage 4 (severe) (at least 18 years old at event) |
|  |  |  | diagnosis | UMLS:ICD10CM:N18.2 | Chronic kidney disease, stage 2 (mild) (at least 18 years old at event) |
|  |  |  | diagnosis | UMLS:ICD10CM:N18.31 | Chronic kidney disease, stage 3a (at least 18 years old at event) |
|  |  |  | diagnosis | UMLS:ICD10CM:N18.32 | Chronic kidney disease, stage 3b (at least 18 years old at event) |
|  |  |  | diagnosis | UMLS:ICD10CM:N18.5 | Chronic kidney disease, stage 5 (at least 18 years old at event) |
|  |  |  | diagnosis | UMLS:ICD10CM:N18.1 | Chronic kidney disease, stage 1 (at least 18 years old at event) |
|  |  |  | diagnosis | UMLS:ICD10CM:N18.6 | End stage renal disease (at least 18 years old at event) |
|  | date constraint | | The terms in this group occurred at any time | | |
|  | event relationship | | Any instance of 排除Schizophrenia occurred at least 1 day before any instance of CKD history | | |
|  | **Group 8B 排除Schizophrenia** | | | | |
|  | cannot have |  | diagnosis | UMLS:ICD10CM:F20 | Schizophrenia |
|  |  | or | diagnosis | UMLS:ICD10CM:F20-F29 | Schizophrenia, schizotypal, delusional, and other non-mood psychotic disorders |
|  |  | or | diagnosis | UMLS:ICD10CM:F20.9 | Schizophrenia, unspecified |
|  |  | or | diagnosis | UMLS:ICD10CM:Z87.898 | Personal history of other specified conditions |
|  |  | or | diagnosis | UMLS:ICD10CM:F84 | Pervasive developmental disorders |
|  |  | or | diagnosis | UMLS:ICD10CM:F29 | Unspecified psychosis not due to a substance or known physiological condition |
| Group 9 | | | | | |
|  | **Group 9A CKD history** | | | | |
|  | must have | any of | diagnosis | UMLS:ICD10CM:N18 | Chronic kidney disease (CKD) (at least 18 years old at event) |
|  |  |  | diagnosis | UMLS:ICD10CM:N18.9 | Chronic kidney disease, unspecified (at least 18 years old at event) |
|  |  |  | diagnosis | UMLS:ICD10CM:N18.3 | Chronic kidney disease, stage 3 (moderate) (at least 18 years old at event) |
|  |  |  | diagnosis | UMLS:ICD10CM:N18.30 | Chronic kidney disease, stage 3 unspecified (at least 18 years old at event) |
|  |  |  | diagnosis | UMLS:ICD10CM:N18.4 | Chronic kidney disease, stage 4 (severe) (at least 18 years old at event) |
|  |  |  | diagnosis | UMLS:ICD10CM:N18.2 | Chronic kidney disease, stage 2 (mild) (at least 18 years old at event) |
|  |  |  | diagnosis | UMLS:ICD10CM:N18.31 | Chronic kidney disease, stage 3a (at least 18 years old at event) |
|  |  |  | diagnosis | UMLS:ICD10CM:N18.32 | Chronic kidney disease, stage 3b (at least 18 years old at event) |
|  |  |  | diagnosis | UMLS:ICD10CM:N18.5 | Chronic kidney disease, stage 5 (at least 18 years old at event) |
|  |  |  | diagnosis | UMLS:ICD10CM:N18.1 | Chronic kidney disease, stage 1 (at least 18 years old at event) |
|  |  |  | diagnosis | UMLS:ICD10CM:N18.6 | End stage renal disease (at least 18 years old at event) |
|  | date constraint | | The terms in this group occurred at any time | | |
|  | event relationship | | Any instance of 排除biploar disorders occurred at least 1 day before any instance of CKD history | | |
|  | **Group 9B 排除biploar disorders** | | | | |
|  | cannot have |  | diagnosis | UMLS:ICD10CM:F31 | Bipolar disorder |
|  |  | or | diagnosis | UMLS:ICD10CM:Z86.59 | Personal history of other mental and behavioral disorders |
|  |  | or | diagnosis | UMLS:ICD10CM:F31.81 | Bipolar II disorder |
|  |  | or | diagnosis | UMLS:ICD10CM:F31.2 | Bipolar disorder, current episode manic severe with psychotic features |
|  |  | or | diagnosis | UMLS:ICD10CM:F31.60 | Bipolar disorder, current episode mixed, unspecified |
| Group 10 | | | | | |
|  | **Group 10A CKD history** | | | | |
|  | must have | any of | diagnosis | UMLS:ICD10CM:N18 | Chronic kidney disease (CKD) (at least 18 years old at event) |
|  |  |  | diagnosis | UMLS:ICD10CM:N18.9 | Chronic kidney disease, unspecified (at least 18 years old at event) |
|  |  |  | diagnosis | UMLS:ICD10CM:N18.3 | Chronic kidney disease, stage 3 (moderate) (at least 18 years old at event) |
|  |  |  | diagnosis | UMLS:ICD10CM:N18.30 | Chronic kidney disease, stage 3 unspecified (at least 18 years old at event) |
|  |  |  | diagnosis | UMLS:ICD10CM:N18.4 | Chronic kidney disease, stage 4 (severe) (at least 18 years old at event) |
|  |  |  | diagnosis | UMLS:ICD10CM:N18.2 | Chronic kidney disease, stage 2 (mild) (at least 18 years old at event) |
|  |  |  | diagnosis | UMLS:ICD10CM:N18.31 | Chronic kidney disease, stage 3a (at least 18 years old at event) |
|  |  |  | diagnosis | UMLS:ICD10CM:N18.32 | Chronic kidney disease, stage 3b (at least 18 years old at event) |
|  |  |  | diagnosis | UMLS:ICD10CM:N18.5 | Chronic kidney disease, stage 5 (at least 18 years old at event) |
|  |  |  | diagnosis | UMLS:ICD10CM:N18.1 | Chronic kidney disease, stage 1 (at least 18 years old at event) |
|  |  |  | diagnosis | UMLS:ICD10CM:N18.6 | End stage renal disease (at least 18 years old at event) |
|  | date constraint | | The terms in this group occurred at any time | | |
|  | event relationship | | Any instance of 排除depressive disorder, occurred at least 1 day before any instance of CKD history | | |
|  | **Group 10B 排除depressive disorder,** | | | | |
|  | cannot have |  | diagnosis | UMLS:ICD10CM:F32 | Depressive episode |
|  |  | or | diagnosis | UMLS:ICD10CM:F32.A | Depression, unspecified |
|  |  | or | diagnosis | UMLS:ICD10CM:F32.8 | Other depressive episodes |
|  |  | or | diagnosis | UMLS:ICD10CM:F34.1 | Dysthymic disorder |
|  |  | or | diagnosis | UMLS:ICD10CM:F33 | Major depressive disorder, recurrent |
| Group 11 | | | | | |
|  | **Group 11A CKD history** | | | | |
|  | must have | any of | diagnosis | UMLS:ICD10CM:N18 | Chronic kidney disease (CKD) (at least 18 years old at event) |
|  |  |  | diagnosis | UMLS:ICD10CM:N18.9 | Chronic kidney disease, unspecified (at least 18 years old at event) |
|  |  |  | diagnosis | UMLS:ICD10CM:N18.3 | Chronic kidney disease, stage 3 (moderate) (at least 18 years old at event) |
|  |  |  | diagnosis | UMLS:ICD10CM:N18.30 | Chronic kidney disease, stage 3 unspecified (at least 18 years old at event) |
|  |  |  | diagnosis | UMLS:ICD10CM:N18.4 | Chronic kidney disease, stage 4 (severe) (at least 18 years old at event) |
|  |  |  | diagnosis | UMLS:ICD10CM:N18.2 | Chronic kidney disease, stage 2 (mild) (at least 18 years old at event) |
|  |  |  | diagnosis | UMLS:ICD10CM:N18.31 | Chronic kidney disease, stage 3a (at least 18 years old at event) |
|  |  |  | diagnosis | UMLS:ICD10CM:N18.32 | Chronic kidney disease, stage 3b (at least 18 years old at event) |
|  |  |  | diagnosis | UMLS:ICD10CM:N18.5 | Chronic kidney disease, stage 5 (at least 18 years old at event) |
|  |  |  | diagnosis | UMLS:ICD10CM:N18.1 | Chronic kidney disease, stage 1 (at least 18 years old at event) |
|  |  |  | diagnosis | UMLS:ICD10CM:N18.6 | End stage renal disease (at least 18 years old at event) |
|  | date constraint | | The terms in this group occurred at any time | | |
|  | event relationship | | Any instance of Substance Use Disorders occurred before or up to 5 years after any instance of CKD history | | |
|  | **Group 11B Substance Use Disorders** | | | | |
|  | cannot have |  | diagnosis | UMLS:ICD10CM:F10-F19 | Mental and behavioral disorders due to psychoactive substance use |
| Group 12 | | | | | |
|  | **Group 12A CKD history** | | | | |
|  | must have | any of | diagnosis | UMLS:ICD10CM:N18 | Chronic kidney disease (CKD) (at least 18 years old at event) |
|  |  |  | diagnosis | UMLS:ICD10CM:N18.9 | Chronic kidney disease, unspecified (at least 18 years old at event) |
|  |  |  | diagnosis | UMLS:ICD10CM:N18.3 | Chronic kidney disease, stage 3 (moderate) (at least 18 years old at event) |
|  |  |  | diagnosis | UMLS:ICD10CM:N18.30 | Chronic kidney disease, stage 3 unspecified (at least 18 years old at event) |
|  |  |  | diagnosis | UMLS:ICD10CM:N18.4 | Chronic kidney disease, stage 4 (severe) (at least 18 years old at event) |
|  |  |  | diagnosis | UMLS:ICD10CM:N18.2 | Chronic kidney disease, stage 2 (mild) (at least 18 years old at event) |
|  |  |  | diagnosis | UMLS:ICD10CM:N18.31 | Chronic kidney disease, stage 3a (at least 18 years old at event) |
|  |  |  | diagnosis | UMLS:ICD10CM:N18.32 | Chronic kidney disease, stage 3b (at least 18 years old at event) |
|  |  |  | diagnosis | UMLS:ICD10CM:N18.5 | Chronic kidney disease, stage 5 (at least 18 years old at event) |
|  |  |  | diagnosis | UMLS:ICD10CM:N18.1 | Chronic kidney disease, stage 1 (at least 18 years old at event) |
|  |  |  | diagnosis | UMLS:ICD10CM:N18.6 | End stage renal disease (at least 18 years old at event) |
|  | date constraint | | The terms in this group occurred at any time | | |
|  | event relationship | | Any instance of 排除stroke or ICH or head injury occurred at least 1 day before any instance of CKD history | | |
|  | **Group 12B 排除stroke or ICH or head injury** | | | | |
|  | cannot have |  | diagnosis | UMLS:ICD10CM:I63 | Cerebral infarction |
|  |  | or | diagnosis | UMLS:ICD10CM:I63.50 | Cerebral infarction due to unspecified occlusion or stenosis of unspecified cerebral artery |
|  |  | or | diagnosis | UMLS:ICD10CM:I63.9 | Cerebral infarction, unspecified |
|  |  | or | diagnosis | UMLS:ICD10CM:I63.5 | Cerebral infarction due to unspecified occlusion or stenosis of cerebral arteries |
|  |  | or | diagnosis | UMLS:ICD10CM:I63.8 | Other cerebral infarction |
|  |  | or | diagnosis | UMLS:ICD10CM:I63.4 | Cerebral infarction due to embolism of cerebral arteries |
|  |  | or | diagnosis | UMLS:ICD10CM:I63.51 | Cerebral infarction due to unspecified occlusion or stenosis of middle cerebral artery |
|  |  | or | diagnosis | UMLS:ICD10CM:I63.3 | Cerebral infarction due to thrombosis of cerebral arteries |
|  |  | or | diagnosis | UMLS:ICD10CM:I62 | Other and unspecified nontraumatic intracranial hemorrhage |
|  |  | or | diagnosis | UMLS:ICD10CM:I62.0 | Nontraumatic subdural hemorrhage |
|  |  | or | diagnosis | UMLS:ICD10CM:I62.00 | Nontraumatic subdural hemorrhage, unspecified |
|  |  | or | diagnosis | UMLS:ICD10CM:I62.9 | Nontraumatic intracranial hemorrhage, unspecified |
|  |  | or | diagnosis | UMLS:ICD10CM:I62.01 | Nontraumatic acute subdural hemorrhage |
|  |  | or | diagnosis | UMLS:ICD10CM:I62.03 | Nontraumatic chronic subdural hemorrhage |
|  |  | or | diagnosis | UMLS:ICD10CM:I62.1 | Nontraumatic extradural hemorrhage |
|  |  | or | diagnosis | UMLS:ICD10CM:I62.02 | Nontraumatic subacute subdural hemorrhage |
|  |  | or | diagnosis | UMLS:ICD10CM:S09 | Other and unspecified injuries of head |
| Group 13 | | | | | |
|  | **Group 13A CKD history** | | | | |
|  | must have | any of | diagnosis | UMLS:ICD10CM:N18 | Chronic kidney disease (CKD) (at least 18 years old at event) |
|  |  |  | diagnosis | UMLS:ICD10CM:N18.9 | Chronic kidney disease, unspecified (at least 18 years old at event) |
|  |  |  | diagnosis | UMLS:ICD10CM:N18.3 | Chronic kidney disease, stage 3 (moderate) (at least 18 years old at event) |
|  |  |  | diagnosis | UMLS:ICD10CM:N18.30 | Chronic kidney disease, stage 3 unspecified (at least 18 years old at event) |
|  |  |  | diagnosis | UMLS:ICD10CM:N18.4 | Chronic kidney disease, stage 4 (severe) (at least 18 years old at event) |
|  |  |  | diagnosis | UMLS:ICD10CM:N18.2 | Chronic kidney disease, stage 2 (mild) (at least 18 years old at event) |
|  |  |  | diagnosis | UMLS:ICD10CM:N18.31 | Chronic kidney disease, stage 3a (at least 18 years old at event) |
|  |  |  | diagnosis | UMLS:ICD10CM:N18.32 | Chronic kidney disease, stage 3b (at least 18 years old at event) |
|  |  |  | diagnosis | UMLS:ICD10CM:N18.5 | Chronic kidney disease, stage 5 (at least 18 years old at event) |
|  |  |  | diagnosis | UMLS:ICD10CM:N18.1 | Chronic kidney disease, stage 1 (at least 18 years old at event) |
|  |  |  | diagnosis | UMLS:ICD10CM:N18.6 | End stage renal disease (at least 18 years old at event) |
|  | date constraint | | The terms in this group occurred at any time | | |
|  | event relationship | | Any instance of 排除三年內mortality occurred within 3 years on or after any instance of CKD history | | |
|  | **Group 13B 排除三年內mortality** | | | | |
|  | cannot have |  | demographics | Deceased | Deceased |

### Query Criteria for Cohort 2 (query name: ###control V2 (>50, depression))

This query was run on the network Global Collaborative Network with 133 HCO(s) queried and 133 HCO(s) responded. A total of 81 provider(s) responded with patients. The final cohort included 83,731 patients who matched the query criteria listed in the table below.

| Ungrouped terms | | | | | |
| --- | --- | --- | --- | --- | --- |
|  | must have |  | demographics | Age | Age (at least 50 years (most recent occurrence)) |
| Group 1 | | | | | |
|  | **Visit>2** | | | | |
|  | must have |  | visit | TNX:Visit | Visit |
|  | number of instances | | Greater than or equal to 2 instances | | |
|  | date constraint | | The terms in this group occurred between Jan 1, 2010 and Dec 31, 2019 | | |
| Group 2 | | | | | |
|  | **Group 2A CKD history** | | | | |
|  | must have | any of | diagnosis | UMLS:ICD10CM:N18 | Chronic kidney disease (CKD) (at least 50 years old at event) |
|  |  |  | diagnosis | UMLS:ICD10CM:N18.9 | Chronic kidney disease, unspecified (at least 50 years old at event) |
|  |  |  | diagnosis | UMLS:ICD10CM:N18.3 | Chronic kidney disease, stage 3 (moderate) (at least 50 years old at event) |
|  |  |  | diagnosis | UMLS:ICD10CM:N18.30 | Chronic kidney disease, stage 3 unspecified (at least 50 years old at event) |
|  |  |  | diagnosis | UMLS:ICD10CM:N18.4 | Chronic kidney disease, stage 4 (severe) (at least 50 years old at event) |
|  |  |  | diagnosis | UMLS:ICD10CM:N18.2 | Chronic kidney disease, stage 2 (mild) (at least 50 years old at event) |
|  |  |  | diagnosis | UMLS:ICD10CM:N18.31 | Chronic kidney disease, stage 3a (at least 50 years old at event) |
|  |  |  | diagnosis | UMLS:ICD10CM:N18.32 | Chronic kidney disease, stage 3b (at least 50 years old at event) |
|  |  |  | diagnosis | UMLS:ICD10CM:N18.5 | Chronic kidney disease, stage 5 (at least 50 years old at event) |
|  |  |  | diagnosis | UMLS:ICD10CM:N18.1 | Chronic kidney disease, stage 1 (at least 50 years old at event) |
|  |  |  | diagnosis | UMLS:ICD10CM:N18.6 | End stage renal disease (at least 50 years old at event) |
|  | date constraint | | The terms in this group occurred at any time | | |
|  | event relationship | | Any instance of 在CKD三個月內vitamin D>30 occurred within 3 months on or after any instance of CKD history | | |
|  | **Group 2B 在CKD三個月內vitamin D>30** | | | | |
|  | must have | any of | laboratory | TNX:9034 | Calcidiol [Mass/volume] in Serum or Plasma (at least 30.00 ng/mL) |
|  |  |  | laboratory | UMLS:LNC:35365-6 | Vitamin D+Metabolites [Mass/volume] in Serum or Plasma (at least 30.00 ng/mL) |
|  |  |  | laboratory | TNX:LG25965-1 | Calcidiol+ercalcidiol [Mass/volume] in Serum, Plasma or Blood (at least 30.00 ng/mL) |
| Group 3 | | | | | |
|  | **Group 3A CKD history** | | | | |
|  | must have | any of | diagnosis | UMLS:ICD10CM:N18 | Chronic kidney disease (CKD) (at least 18 years old at event) |
|  |  |  | diagnosis | UMLS:ICD10CM:N18.9 | Chronic kidney disease, unspecified (at least 18 years old at event) |
|  |  |  | diagnosis | UMLS:ICD10CM:N18.3 | Chronic kidney disease, stage 3 (moderate) (at least 18 years old at event) |
|  |  |  | diagnosis | UMLS:ICD10CM:N18.30 | Chronic kidney disease, stage 3 unspecified (at least 18 years old at event) |
|  |  |  | diagnosis | UMLS:ICD10CM:N18.4 | Chronic kidney disease, stage 4 (severe) (at least 18 years old at event) |
|  |  |  | diagnosis | UMLS:ICD10CM:N18.2 | Chronic kidney disease, stage 2 (mild) (at least 18 years old at event) |
|  |  |  | diagnosis | UMLS:ICD10CM:N18.31 | Chronic kidney disease, stage 3a (at least 18 years old at event) |
|  |  |  | diagnosis | UMLS:ICD10CM:N18.32 | Chronic kidney disease, stage 3b (at least 18 years old at event) |
|  |  |  | diagnosis | UMLS:ICD10CM:N18.5 | Chronic kidney disease, stage 5 (at least 18 years old at event) |
|  |  |  | diagnosis | UMLS:ICD10CM:N18.1 | Chronic kidney disease, stage 1 (at least 18 years old at event) |
|  |  |  | diagnosis | UMLS:ICD10CM:N18.6 | End stage renal disease (at least 18 years old at event) |
|  | date constraint | | The terms in this group occurred at any time | | |
|  | event relationship | | Any instance of 排除在CKD三個月內vitamin D<29 occurred within 3 months on or after any instance of CKD history | | |
|  | **Group 3B 排除在CKD三個月內vitamin D<29** | | | | |
|  | cannot have |  | laboratory | TNX:LG25965-1 | Calcidiol+ercalcidiol [Mass/volume] in Serum, Plasma or Blood (at most 29.00 ng/mL) |
|  |  | or | laboratory | TNX:9034 | Calcidiol [Mass/volume] in Serum or Plasma (at most 29.00 ng/mL) |
|  |  | or | laboratory | UMLS:LNC:35365-6 | Vitamin D+Metabolites [Mass/volume] in Serum or Plasma (at most 29.00 ng/mL) |
| Group 4 | | | | | |
|  | **Group 4A CKD history** | | | | |
|  | must have | any of | diagnosis | UMLS:ICD10CM:N18 | Chronic kidney disease (CKD) (at least 18 years old at event) |
|  |  |  | diagnosis | UMLS:ICD10CM:N18.9 | Chronic kidney disease, unspecified (at least 18 years old at event) |
|  |  |  | diagnosis | UMLS:ICD10CM:N18.3 | Chronic kidney disease, stage 3 (moderate) (at least 18 years old at event) |
|  |  |  | diagnosis | UMLS:ICD10CM:N18.30 | Chronic kidney disease, stage 3 unspecified (at least 18 years old at event) |
|  |  |  | diagnosis | UMLS:ICD10CM:N18.4 | Chronic kidney disease, stage 4 (severe) (at least 18 years old at event) |
|  |  |  | diagnosis | UMLS:ICD10CM:N18.2 | Chronic kidney disease, stage 2 (mild) (at least 18 years old at event) |
|  |  |  | diagnosis | UMLS:ICD10CM:N18.31 | Chronic kidney disease, stage 3a (at least 18 years old at event) |
|  |  |  | diagnosis | UMLS:ICD10CM:N18.32 | Chronic kidney disease, stage 3b (at least 18 years old at event) |
|  |  |  | diagnosis | UMLS:ICD10CM:N18.5 | Chronic kidney disease, stage 5 (at least 18 years old at event) |
|  |  |  | diagnosis | UMLS:ICD10CM:N18.1 | Chronic kidney disease, stage 1 (at least 18 years old at event) |
|  |  |  | diagnosis | UMLS:ICD10CM:N18.6 | End stage renal disease (at least 18 years old at event) |
|  | date constraint | | The terms in this group occurred at any time | | |
|  | event relationship | | Any instance of 排除cognitive impairment occurred at least 1 day before any instance of CKD history | | |
|  | **Group 4B 排除cognitive impairment** | | | | |
|  | cannot have |  | diagnosis | UMLS:ICD10CM:B20 | Human immunodeficiency virus [HIV] disease |
|  |  | or | diagnosis | UMLS:ICD10CM:G31.84 | Mild cognitive impairment of uncertain or unknown etiology |
|  |  | or | diagnosis | UMLS:ICD10CM:F06 | Other mental disorders due to known physiological condition |
|  |  | or | diagnosis | UMLS:ICD10CM:F06.8 | Other specified mental disorders due to known physiological condition |
|  |  | or | diagnosis | UMLS:ICD10CM:F88 | Other disorders of psychological development |
|  |  | or | procedure | UMLS:ICD10PCS:GZ52ZZZ | Individual Psychotherapy, Cognitive |
|  |  | or | diagnosis | UMLS:ICD10CM:F06.7 | Mild neurocognitive disorder due to known physiological condition |
|  |  | or | diagnosis | UMLS:ICD10CM:F09 | Unspecified mental disorder due to known physiological condition |
| Group 5 | | | | | |
|  | **Group 5A CKD history** | | | | |
|  | must have | any of | diagnosis | UMLS:ICD10CM:N18 | Chronic kidney disease (CKD) (at least 18 years old at event) |
|  |  |  | diagnosis | UMLS:ICD10CM:N18.9 | Chronic kidney disease, unspecified (at least 18 years old at event) |
|  |  |  | diagnosis | UMLS:ICD10CM:N18.3 | Chronic kidney disease, stage 3 (moderate) (at least 18 years old at event) |
|  |  |  | diagnosis | UMLS:ICD10CM:N18.30 | Chronic kidney disease, stage 3 unspecified (at least 18 years old at event) |
|  |  |  | diagnosis | UMLS:ICD10CM:N18.4 | Chronic kidney disease, stage 4 (severe) (at least 18 years old at event) |
|  |  |  | diagnosis | UMLS:ICD10CM:N18.2 | Chronic kidney disease, stage 2 (mild) (at least 18 years old at event) |
|  |  |  | diagnosis | UMLS:ICD10CM:N18.31 | Chronic kidney disease, stage 3a (at least 18 years old at event) |
|  |  |  | diagnosis | UMLS:ICD10CM:N18.32 | Chronic kidney disease, stage 3b (at least 18 years old at event) |
|  |  |  | diagnosis | UMLS:ICD10CM:N18.5 | Chronic kidney disease, stage 5 (at least 18 years old at event) |
|  |  |  | diagnosis | UMLS:ICD10CM:N18.1 | Chronic kidney disease, stage 1 (at least 18 years old at event) |
|  |  |  | diagnosis | UMLS:ICD10CM:N18.6 | End stage renal disease (at least 18 years old at event) |
|  | date constraint | | The terms in this group occurred at any time | | |
|  | event relationship | | Any instance of 排除dementia，Alzheimer's disease, Parkinsom disease occurred at least 1 day before any instance of CKD history | | |
|  | **Group 5B 排除dementia，Alzheimer's disease, Parkinsom disease** | | | | |
|  | cannot have |  | diagnosis | UMLS:ICD10CM:F03 | Unspecified dementia |
|  |  | or | diagnosis | UMLS:ICD10CM:F03.90 | Unspecified dementia, unspecified severity, without behavioral disturbance, psychotic disturbance, mood disturbance, and anxiety |
|  |  | or | diagnosis | UMLS:ICD10CM:F02.80 | Dementia in other diseases classified elsewhere, unspecified severity, without behavioral disturbance, psychotic disturbance, mood disturbance, and anxiety |
|  |  | or | diagnosis | UMLS:ICD10CM:F06.0 | Psychotic disorder with hallucinations due to known physiological condition |
|  |  | or | diagnosis | UMLS:ICD10CM:G30 | Alzheimer's disease |
|  |  | or | diagnosis | UMLS:ICD10CM:G20 | Parkinson's disease |
|  |  | or | diagnosis | UMLS:ICD10CM:F05 | Delirium due to known physiological condition |
|  |  | or | diagnosis | UMLS:ICD10CM:G30 | Alzheimer's disease |
|  |  | or | diagnosis | UMLS:ICD10CM:F02.80 | Dementia in other diseases classified elsewhere, unspecified severity, without behavioral disturbance, psychotic disturbance, mood disturbance, and anxiety |
|  |  | or | diagnosis | UMLS:ICD10CM:G30.9 | Alzheimer's disease, unspecified |
|  |  | or | diagnosis | UMLS:ICD10CM:G30.8 | Other Alzheimer's disease |
|  |  | or | diagnosis | UMLS:ICD10CM:F05 | Delirium due to known physiological condition |
|  |  | or | diagnosis | UMLS:ICD10CM:F06.2 | Psychotic disorder with delusions due to known physiological condition |
|  |  | or | diagnosis | UMLS:ICD10CM:G30.1 | Alzheimer's disease with late onset |
|  |  | or | diagnosis | UMLS:ICD10CM:G30.0 | Alzheimer's disease with early onset |
|  |  | or | diagnosis | UMLS:ICD10CM:G30.1 | Alzheimer's disease with late onset |
|  |  | or | diagnosis | UMLS:ICD10CM:G30.8 | Other Alzheimer's disease |
|  |  | or | diagnosis | UMLS:ICD10CM:G30.9 | Alzheimer's disease, unspecified |
|  |  | or | diagnosis | UMLS:ICD10CM:F02.A | Dementia in other diseases classified elsewhere, mild |
|  |  | or | diagnosis | UMLS:ICD10CM:F02.C | Dementia in other diseases classified elsewhere, severe |
|  |  | or | diagnosis | UMLS:ICD10CM:F02.A0 | Dementia in other diseases classified elsewhere, mild, without behavioral disturbance, psychotic disturbance, mood disturbance, and anxiety |
|  |  | or | diagnosis | UMLS:ICD10CM:F02.B0 | Dementia in other diseases classified elsewhere, moderate, without behavioral disturbance, psychotic disturbance, mood disturbance, and anxiety |
|  |  | or | diagnosis | UMLS:ICD10CM:F02.83 | Dementia in other diseases classified elsewhere, unspecified severity, with mood disturbance |
|  |  | or | diagnosis | UMLS:ICD10CM:F02.811 | Dementia in other diseases classified elsewhere, unspecified severity, with agitation |
|  |  | or | diagnosis | UMLS:ICD10CM:F02.B | Dementia in other diseases classified elsewhere, moderate |
|  |  | or | diagnosis | UMLS:ICD10CM:F02.818 | Dementia in other diseases classified elsewhere, unspecified severity, with other behavioral disturbance |
|  |  | or | diagnosis | UMLS:ICD10CM:F02.81 | Dementia in other diseases classified elsewhere, unspecified severity, with behavioral disturbance |
|  |  | or | diagnosis | UMLS:ICD10CM:F03 | Unspecified dementia |
|  |  | or | diagnosis | UMLS:ICD10CM:F03.90 | Unspecified dementia, unspecified severity, without behavioral disturbance, psychotic disturbance, mood disturbance, and anxiety |
|  |  | or | diagnosis | UMLS:ICD10CM:F02.80 | Dementia in other diseases classified elsewhere, unspecified severity, without behavioral disturbance, psychotic disturbance, mood disturbance, and anxiety |
|  |  | or | diagnosis | UMLS:ICD10CM:F06.0 | Psychotic disorder with hallucinations due to known physiological condition |
|  |  | or | diagnosis | UMLS:ICD10CM:F02 | Dementia in other diseases classified elsewhere |
|  |  | or | diagnosis | UMLS:ICD10CM:F02.8 | Dementia in other diseases classified elsewhere, unspecified severity |
| Group 6 | | | | | |
|  | **排除renal transplanation** | | | | |
|  | cannot have |  | procedure | UMLS:CPT:1008098 | Renal Transplantation Procedures |
|  | date constraint | | The terms in this group occurred at any time | | |
| Group 7 | | | | | |
|  | **Group 7A CKD history** | | | | |
|  | must have | any of | diagnosis | UMLS:ICD10CM:N18 | Chronic kidney disease (CKD) (at least 18 years old at event) |
|  |  |  | diagnosis | UMLS:ICD10CM:N18.9 | Chronic kidney disease, unspecified (at least 18 years old at event) |
|  |  |  | diagnosis | UMLS:ICD10CM:N18.3 | Chronic kidney disease, stage 3 (moderate) (at least 18 years old at event) |
|  |  |  | diagnosis | UMLS:ICD10CM:N18.30 | Chronic kidney disease, stage 3 unspecified (at least 18 years old at event) |
|  |  |  | diagnosis | UMLS:ICD10CM:N18.4 | Chronic kidney disease, stage 4 (severe) (at least 18 years old at event) |
|  |  |  | diagnosis | UMLS:ICD10CM:N18.2 | Chronic kidney disease, stage 2 (mild) (at least 18 years old at event) |
|  |  |  | diagnosis | UMLS:ICD10CM:N18.31 | Chronic kidney disease, stage 3a (at least 18 years old at event) |
|  |  |  | diagnosis | UMLS:ICD10CM:N18.32 | Chronic kidney disease, stage 3b (at least 18 years old at event) |
|  |  |  | diagnosis | UMLS:ICD10CM:N18.5 | Chronic kidney disease, stage 5 (at least 18 years old at event) |
|  |  |  | diagnosis | UMLS:ICD10CM:N18.1 | Chronic kidney disease, stage 1 (at least 18 years old at event) |
|  |  |  | diagnosis | UMLS:ICD10CM:N18.6 | End stage renal disease (at least 18 years old at event) |
|  | date constraint | | The terms in this group occurred at any time | | |
|  | event relationship | | Any instance of 排除vascular dementia occurred at least 1 day before any instance of CKD history | | |
|  | **Group 7B 排除vascular dementia** | | | | |
|  | cannot have |  | diagnosis | UMLS:ICD10CM:F01 | Vascular dementia |
|  |  | or | diagnosis | UMLS:ICD10CM:I67.2 | Cerebral atherosclerosis |
|  |  | or | diagnosis | UMLS:ICD10CM:F01.50 | Vascular dementia, unspecified severity, without behavioral disturbance, psychotic disturbance, mood disturbance, and anxiety |
|  |  | or | diagnosis | UMLS:ICD10CM:F01.5 | Vascular dementia, unspecified severity |
|  |  | or | diagnosis | UMLS:ICD10CM:F01.A | Vascular dementia, mild |
|  |  | or | diagnosis | UMLS:ICD10CM:F01.B | Vascular dementia, moderate |
|  |  | or | diagnosis | UMLS:ICD10CM:F01.A0 | Vascular dementia, mild, without behavioral disturbance, psychotic disturbance, mood disturbance, and anxiety |
|  |  | or | diagnosis | UMLS:ICD10CM:F01.B0 | Vascular dementia, moderate, without behavioral disturbance, psychotic disturbance, mood disturbance, and anxiety |
|  |  | or | diagnosis | UMLS:ICD10CM:F01.C | Vascular dementia, severe |
| Group 8 | | | | | |
|  | **Group 8A CKD history** | | | | |
|  | must have | any of | diagnosis | UMLS:ICD10CM:N18 | Chronic kidney disease (CKD) (at least 18 years old at event) |
|  |  |  | diagnosis | UMLS:ICD10CM:N18.9 | Chronic kidney disease, unspecified (at least 18 years old at event) |
|  |  |  | diagnosis | UMLS:ICD10CM:N18.3 | Chronic kidney disease, stage 3 (moderate) (at least 18 years old at event) |
|  |  |  | diagnosis | UMLS:ICD10CM:N18.30 | Chronic kidney disease, stage 3 unspecified (at least 18 years old at event) |
|  |  |  | diagnosis | UMLS:ICD10CM:N18.4 | Chronic kidney disease, stage 4 (severe) (at least 18 years old at event) |
|  |  |  | diagnosis | UMLS:ICD10CM:N18.2 | Chronic kidney disease, stage 2 (mild) (at least 18 years old at event) |
|  |  |  | diagnosis | UMLS:ICD10CM:N18.31 | Chronic kidney disease, stage 3a (at least 18 years old at event) |
|  |  |  | diagnosis | UMLS:ICD10CM:N18.32 | Chronic kidney disease, stage 3b (at least 18 years old at event) |
|  |  |  | diagnosis | UMLS:ICD10CM:N18.5 | Chronic kidney disease, stage 5 (at least 18 years old at event) |
|  |  |  | diagnosis | UMLS:ICD10CM:N18.1 | Chronic kidney disease, stage 1 (at least 18 years old at event) |
|  |  |  | diagnosis | UMLS:ICD10CM:N18.6 | End stage renal disease (at least 18 years old at event) |
|  | date constraint | | The terms in this group occurred at any time | | |
|  | event relationship | | Any instance of 排除Schizophrenia occurred at least 1 day before any instance of CKD history | | |
|  | **Group 8B 排除Schizophrenia** | | | | |
|  | cannot have |  | diagnosis | UMLS:ICD10CM:F20 | Schizophrenia |
|  |  | or | diagnosis | UMLS:ICD10CM:F20-F29 | Schizophrenia, schizotypal, delusional, and other non-mood psychotic disorders |
|  |  | or | diagnosis | UMLS:ICD10CM:F20.9 | Schizophrenia, unspecified |
|  |  | or | diagnosis | UMLS:ICD10CM:Z87.898 | Personal history of other specified conditions |
|  |  | or | diagnosis | UMLS:ICD10CM:F84 | Pervasive developmental disorders |
|  |  | or | diagnosis | UMLS:ICD10CM:F29 | Unspecified psychosis not due to a substance or known physiological condition |
| Group 9 | | | | | |
|  | **Group 9A CKD history** | | | | |
|  | must have | any of | diagnosis | UMLS:ICD10CM:N18 | Chronic kidney disease (CKD) (at least 18 years old at event) |
|  |  |  | diagnosis | UMLS:ICD10CM:N18.9 | Chronic kidney disease, unspecified (at least 18 years old at event) |
|  |  |  | diagnosis | UMLS:ICD10CM:N18.3 | Chronic kidney disease, stage 3 (moderate) (at least 18 years old at event) |
|  |  |  | diagnosis | UMLS:ICD10CM:N18.30 | Chronic kidney disease, stage 3 unspecified (at least 18 years old at event) |
|  |  |  | diagnosis | UMLS:ICD10CM:N18.4 | Chronic kidney disease, stage 4 (severe) (at least 18 years old at event) |
|  |  |  | diagnosis | UMLS:ICD10CM:N18.2 | Chronic kidney disease, stage 2 (mild) (at least 18 years old at event) |
|  |  |  | diagnosis | UMLS:ICD10CM:N18.31 | Chronic kidney disease, stage 3a (at least 18 years old at event) |
|  |  |  | diagnosis | UMLS:ICD10CM:N18.32 | Chronic kidney disease, stage 3b (at least 18 years old at event) |
|  |  |  | diagnosis | UMLS:ICD10CM:N18.5 | Chronic kidney disease, stage 5 (at least 18 years old at event) |
|  |  |  | diagnosis | UMLS:ICD10CM:N18.1 | Chronic kidney disease, stage 1 (at least 18 years old at event) |
|  |  |  | diagnosis | UMLS:ICD10CM:N18.6 | End stage renal disease (at least 18 years old at event) |
|  | date constraint | | The terms in this group occurred at any time | | |
|  | event relationship | | Any instance of 排除biploar disorders occurred at least 1 day before any instance of CKD history | | |
|  | **Group 9B 排除biploar disorders** | | | | |
|  | cannot have |  | diagnosis | UMLS:ICD10CM:F31 | Bipolar disorder |
|  |  | or | diagnosis | UMLS:ICD10CM:Z86.59 | Personal history of other mental and behavioral disorders |
|  |  | or | diagnosis | UMLS:ICD10CM:F31.81 | Bipolar II disorder |
|  |  | or | diagnosis | UMLS:ICD10CM:F31.2 | Bipolar disorder, current episode manic severe with psychotic features |
|  |  | or | diagnosis | UMLS:ICD10CM:F31.60 | Bipolar disorder, current episode mixed, unspecified |
| Group 10 | | | | | |
|  | **Group 10A CKD history** | | | | |
|  | must have | any of | diagnosis | UMLS:ICD10CM:N18 | Chronic kidney disease (CKD) (at least 18 years old at event) |
|  |  |  | diagnosis | UMLS:ICD10CM:N18.9 | Chronic kidney disease, unspecified (at least 18 years old at event) |
|  |  |  | diagnosis | UMLS:ICD10CM:N18.3 | Chronic kidney disease, stage 3 (moderate) (at least 18 years old at event) |
|  |  |  | diagnosis | UMLS:ICD10CM:N18.30 | Chronic kidney disease, stage 3 unspecified (at least 18 years old at event) |
|  |  |  | diagnosis | UMLS:ICD10CM:N18.4 | Chronic kidney disease, stage 4 (severe) (at least 18 years old at event) |
|  |  |  | diagnosis | UMLS:ICD10CM:N18.2 | Chronic kidney disease, stage 2 (mild) (at least 18 years old at event) |
|  |  |  | diagnosis | UMLS:ICD10CM:N18.31 | Chronic kidney disease, stage 3a (at least 18 years old at event) |
|  |  |  | diagnosis | UMLS:ICD10CM:N18.32 | Chronic kidney disease, stage 3b (at least 18 years old at event) |
|  |  |  | diagnosis | UMLS:ICD10CM:N18.5 | Chronic kidney disease, stage 5 (at least 18 years old at event) |
|  |  |  | diagnosis | UMLS:ICD10CM:N18.1 | Chronic kidney disease, stage 1 (at least 18 years old at event) |
|  |  |  | diagnosis | UMLS:ICD10CM:N18.6 | End stage renal disease (at least 18 years old at event) |
|  | date constraint | | The terms in this group occurred at any time | | |
|  | event relationship | | Any instance of 排除depressive disorder, occurred at least 1 day before any instance of CKD history | | |
|  | **Group 10B 排除depressive disorder,** | | | | |
|  | cannot have |  | diagnosis | UMLS:ICD10CM:F32 | Depressive episode |
|  |  | or | diagnosis | UMLS:ICD10CM:F32.A | Depression, unspecified |
|  |  | or | diagnosis | UMLS:ICD10CM:F32.8 | Other depressive episodes |
|  |  | or | diagnosis | UMLS:ICD10CM:F34.1 | Dysthymic disorder |
|  |  | or | diagnosis | UMLS:ICD10CM:F33 | Major depressive disorder, recurrent |
| Group 11 | | | | | |
|  | **Group 11A CKD history** | | | | |
|  | must have | any of | diagnosis | UMLS:ICD10CM:N18 | Chronic kidney disease (CKD) (at least 18 years old at event) |
|  |  |  | diagnosis | UMLS:ICD10CM:N18.9 | Chronic kidney disease, unspecified (at least 18 years old at event) |
|  |  |  | diagnosis | UMLS:ICD10CM:N18.3 | Chronic kidney disease, stage 3 (moderate) (at least 18 years old at event) |
|  |  |  | diagnosis | UMLS:ICD10CM:N18.30 | Chronic kidney disease, stage 3 unspecified (at least 18 years old at event) |
|  |  |  | diagnosis | UMLS:ICD10CM:N18.4 | Chronic kidney disease, stage 4 (severe) (at least 18 years old at event) |
|  |  |  | diagnosis | UMLS:ICD10CM:N18.2 | Chronic kidney disease, stage 2 (mild) (at least 18 years old at event) |
|  |  |  | diagnosis | UMLS:ICD10CM:N18.31 | Chronic kidney disease, stage 3a (at least 18 years old at event) |
|  |  |  | diagnosis | UMLS:ICD10CM:N18.32 | Chronic kidney disease, stage 3b (at least 18 years old at event) |
|  |  |  | diagnosis | UMLS:ICD10CM:N18.5 | Chronic kidney disease, stage 5 (at least 18 years old at event) |
|  |  |  | diagnosis | UMLS:ICD10CM:N18.1 | Chronic kidney disease, stage 1 (at least 18 years old at event) |
|  |  |  | diagnosis | UMLS:ICD10CM:N18.6 | End stage renal disease (at least 18 years old at event) |
|  | date constraint | | The terms in this group occurred at any time | | |
|  | event relationship | | Any instance of Substance Use Disorders occurred before or up to 5 years after any instance of CKD history | | |
|  | **Group 11B Substance Use Disorders** | | | | |
|  | cannot have |  | diagnosis | UMLS:ICD10CM:F10-F19 | Mental and behavioral disorders due to psychoactive substance use |
| Group 12 | | | | | |
|  | **Group 12A CKD history** | | | | |
|  | must have | any of | diagnosis | UMLS:ICD10CM:N18 | Chronic kidney disease (CKD) (at least 18 years old at event) |
|  |  |  | diagnosis | UMLS:ICD10CM:N18.9 | Chronic kidney disease, unspecified (at least 18 years old at event) |
|  |  |  | diagnosis | UMLS:ICD10CM:N18.3 | Chronic kidney disease, stage 3 (moderate) (at least 18 years old at event) |
|  |  |  | diagnosis | UMLS:ICD10CM:N18.30 | Chronic kidney disease, stage 3 unspecified (at least 18 years old at event) |
|  |  |  | diagnosis | UMLS:ICD10CM:N18.4 | Chronic kidney disease, stage 4 (severe) (at least 18 years old at event) |
|  |  |  | diagnosis | UMLS:ICD10CM:N18.2 | Chronic kidney disease, stage 2 (mild) (at least 18 years old at event) |
|  |  |  | diagnosis | UMLS:ICD10CM:N18.31 | Chronic kidney disease, stage 3a (at least 18 years old at event) |
|  |  |  | diagnosis | UMLS:ICD10CM:N18.32 | Chronic kidney disease, stage 3b (at least 18 years old at event) |
|  |  |  | diagnosis | UMLS:ICD10CM:N18.5 | Chronic kidney disease, stage 5 (at least 18 years old at event) |
|  |  |  | diagnosis | UMLS:ICD10CM:N18.1 | Chronic kidney disease, stage 1 (at least 18 years old at event) |
|  |  |  | diagnosis | UMLS:ICD10CM:N18.6 | End stage renal disease (at least 18 years old at event) |
|  | date constraint | | The terms in this group occurred at any time | | |
|  | event relationship | | Any instance of 排除stroke or ICH or head injury occurred at least 1 day before any instance of CKD history | | |
|  | **Group 12B 排除stroke or ICH or head injury** | | | | |
|  | cannot have |  | diagnosis | UMLS:ICD10CM:I63 | Cerebral infarction |
|  |  | or | diagnosis | UMLS:ICD10CM:I63.50 | Cerebral infarction due to unspecified occlusion or stenosis of unspecified cerebral artery |
|  |  | or | diagnosis | UMLS:ICD10CM:I63.9 | Cerebral infarction, unspecified |
|  |  | or | diagnosis | UMLS:ICD10CM:I63.5 | Cerebral infarction due to unspecified occlusion or stenosis of cerebral arteries |
|  |  | or | diagnosis | UMLS:ICD10CM:I63.8 | Other cerebral infarction |
|  |  | or | diagnosis | UMLS:ICD10CM:I63.4 | Cerebral infarction due to embolism of cerebral arteries |
|  |  | or | diagnosis | UMLS:ICD10CM:I63.51 | Cerebral infarction due to unspecified occlusion or stenosis of middle cerebral artery |
|  |  | or | diagnosis | UMLS:ICD10CM:I63.3 | Cerebral infarction due to thrombosis of cerebral arteries |
|  |  | or | diagnosis | UMLS:ICD10CM:I62 | Other and unspecified nontraumatic intracranial hemorrhage |
|  |  | or | diagnosis | UMLS:ICD10CM:I62.0 | Nontraumatic subdural hemorrhage |
|  |  | or | diagnosis | UMLS:ICD10CM:I62.00 | Nontraumatic subdural hemorrhage, unspecified |
|  |  | or | diagnosis | UMLS:ICD10CM:I62.9 | Nontraumatic intracranial hemorrhage, unspecified |
|  |  | or | diagnosis | UMLS:ICD10CM:I62.01 | Nontraumatic acute subdural hemorrhage |
|  |  | or | diagnosis | UMLS:ICD10CM:I62.03 | Nontraumatic chronic subdural hemorrhage |
|  |  | or | diagnosis | UMLS:ICD10CM:I62.1 | Nontraumatic extradural hemorrhage |
|  |  | or | diagnosis | UMLS:ICD10CM:I62.02 | Nontraumatic subacute subdural hemorrhage |
|  |  | or | diagnosis | UMLS:ICD10CM:S09 | Other and unspecified injuries of head |
| Group 13 | | | | | |
|  | **Group 13A CKD history** | | | | |
|  | must have | any of | diagnosis | UMLS:ICD10CM:N18 | Chronic kidney disease (CKD) (at least 18 years old at event) |
|  |  |  | diagnosis | UMLS:ICD10CM:N18.9 | Chronic kidney disease, unspecified (at least 18 years old at event) |
|  |  |  | diagnosis | UMLS:ICD10CM:N18.3 | Chronic kidney disease, stage 3 (moderate) (at least 18 years old at event) |
|  |  |  | diagnosis | UMLS:ICD10CM:N18.30 | Chronic kidney disease, stage 3 unspecified (at least 18 years old at event) |
|  |  |  | diagnosis | UMLS:ICD10CM:N18.4 | Chronic kidney disease, stage 4 (severe) (at least 18 years old at event) |
|  |  |  | diagnosis | UMLS:ICD10CM:N18.2 | Chronic kidney disease, stage 2 (mild) (at least 18 years old at event) |
|  |  |  | diagnosis | UMLS:ICD10CM:N18.31 | Chronic kidney disease, stage 3a (at least 18 years old at event) |
|  |  |  | diagnosis | UMLS:ICD10CM:N18.32 | Chronic kidney disease, stage 3b (at least 18 years old at event) |
|  |  |  | diagnosis | UMLS:ICD10CM:N18.5 | Chronic kidney disease, stage 5 (at least 18 years old at event) |
|  |  |  | diagnosis | UMLS:ICD10CM:N18.1 | Chronic kidney disease, stage 1 (at least 18 years old at event) |
|  |  |  | diagnosis | UMLS:ICD10CM:N18.6 | End stage renal disease (at least 18 years old at event) |
|  | date constraint | | The terms in this group occurred at any time | | |
|  | event relationship | | Any instance of 排除三年內mortality occurred within 3 years on or after any instance of CKD history | | |
|  | **Group 13B 排除三年內mortality** | | | | |
|  | cannot have |  | demographics | Deceased | Deceased |

## Analysis Setup

This section contains the Index Event and Time Window definitions and a list of selected outcomes and the analyses.

### Index Event & Time Window Definitions

The index event defines the point in time when each patient in the cohort enters the analysis. To define an index event for the cohort, one or more criteria for the cohort must be selected. The index date for each patient within a cohort is the day on which the patient first met the selected criteria for the cohort (listed in the table below).

As the index event defines the earliest time point after which outcomes are analyzed, the time window defines the duration during which outcomes are analyzed. The time window can start on the same day as the index event or at any specified time interval after the index event. The time window can end any time after the start date. Outcomes are defined as diagnoses, medications, procedures, or laboratory values that happened in the time window starting after the first occurrence of the index event.

### Time Window Used in this Analysis

This analysis included outcomes that occurred in the time window that started 1 day after the first occurrence of the index event and ended 365 days after the first occurrence of the index event.

The index event only includes events that occurred up to 20 years ago. Patients whose index event occurred 20 years or more ago are excluded. In this analysis, 0 patients in Cohort 1 and 0 patients in Cohort 2 were excluded because they met the index event more than 20 years ago.

### Index Events Used in this Analysis

Index events for the Compare Outcomes analysis were derived from the cohort definitions. Index events were defined separately for each cohort and were based on the criteria used in the original cohort definition. Please see Appendix B for the text representation of the index event definition.

The index event for Cohort 1 (query name: ###VDD v2 (>50, depression) was defined as the following:

|  | | | | | |
| --- | --- | --- | --- | --- | --- |
| Group 1 | | | | | |
|  | **Group 1A CKD history** | | | | |
|  | must have | any of | diagnosis | UMLS:ICD10CM:N18 | Chronic kidney disease (CKD) (at least 18 years old at event) |
|  |  |  | diagnosis | UMLS:ICD10CM:N18.9 | Chronic kidney disease, unspecified (at least 18 years old at event) |
|  |  |  | diagnosis | UMLS:ICD10CM:N18.3 | Chronic kidney disease, stage 3 (moderate) (at least 18 years old at event) |
|  |  |  | diagnosis | UMLS:ICD10CM:N18.30 | Chronic kidney disease, stage 3 unspecified (at least 18 years old at event) |
|  |  |  | diagnosis | UMLS:ICD10CM:N18.4 | Chronic kidney disease, stage 4 (severe) (at least 18 years old at event) |
|  |  |  | diagnosis | UMLS:ICD10CM:N18.2 | Chronic kidney disease, stage 2 (mild) (at least 18 years old at event) |
|  |  |  | diagnosis | UMLS:ICD10CM:N18.31 | Chronic kidney disease, stage 3a (at least 18 years old at event) |
|  |  |  | diagnosis | UMLS:ICD10CM:N18.32 | Chronic kidney disease, stage 3b (at least 18 years old at event) |
|  |  |  | diagnosis | UMLS:ICD10CM:N18.5 | Chronic kidney disease, stage 5 (at least 18 years old at event) |
|  |  |  | diagnosis | UMLS:ICD10CM:N18.1 | Chronic kidney disease, stage 1 (at least 18 years old at event) |
|  |  |  | diagnosis | UMLS:ICD10CM:N18.6 | End stage renal disease (at least 18 years old at event) |
|  | date constraint | | The terms in this group occurred at any time | | |
|  | event relationship | | Any instance of CKD三個月內vitamin D<20 occurred within 3 months on or after any instance of CKD history | | |
|  | **Group 1B CKD三個月內vitamin D<20** | | | | |
|  | must have | any of | laboratory | UMLS:LNC:35365-6 | Vitamin D+Metabolites [Mass/volume] in Serum or Plasma (at most 20.00 ng/mL) |
|  |  |  | laboratory | TNX:LG25965-1 | Calcidiol+ercalcidiol [Mass/volume] in Serum, Plasma or Blood (at most 20.00 ng/mL) |
|  |  |  | laboratory | TNX:9034 | Calcidiol [Mass/volume] in Serum or Plasma (at most 20.00 ng/mL) |

The index event for Cohort 2 (query name: ###control V2 (>50, depression)) was defined as the following:

|  | | | | | |
| --- | --- | --- | --- | --- | --- |
| Group 1 | | | | | |
|  | **Group 1A CKD history** | | | | |
|  | must have | any of | diagnosis | UMLS:ICD10CM:N18 | Chronic kidney disease (CKD) (at least 50 years old at event) |
|  |  |  | diagnosis | UMLS:ICD10CM:N18.9 | Chronic kidney disease, unspecified (at least 50 years old at event) |
|  |  |  | diagnosis | UMLS:ICD10CM:N18.3 | Chronic kidney disease, stage 3 (moderate) (at least 50 years old at event) |
|  |  |  | diagnosis | UMLS:ICD10CM:N18.30 | Chronic kidney disease, stage 3 unspecified (at least 50 years old at event) |
|  |  |  | diagnosis | UMLS:ICD10CM:N18.4 | Chronic kidney disease, stage 4 (severe) (at least 50 years old at event) |
|  |  |  | diagnosis | UMLS:ICD10CM:N18.2 | Chronic kidney disease, stage 2 (mild) (at least 50 years old at event) |
|  |  |  | diagnosis | UMLS:ICD10CM:N18.31 | Chronic kidney disease, stage 3a (at least 50 years old at event) |
|  |  |  | diagnosis | UMLS:ICD10CM:N18.32 | Chronic kidney disease, stage 3b (at least 50 years old at event) |
|  |  |  | diagnosis | UMLS:ICD10CM:N18.5 | Chronic kidney disease, stage 5 (at least 50 years old at event) |
|  |  |  | diagnosis | UMLS:ICD10CM:N18.1 | Chronic kidney disease, stage 1 (at least 50 years old at event) |
|  |  |  | diagnosis | UMLS:ICD10CM:N18.6 | End stage renal disease (at least 50 years old at event) |
|  | date constraint | | The terms in this group occurred at any time | | |
|  | event relationship | | Any instance of 在CKD三個月內vitamin D>30 occurred within 3 months on or after any instance of CKD history | | |
|  | **Group 1B 在CKD三個月內vitamin D>30** | | | | |
|  | must have | any of | laboratory | TNX:9034 | Calcidiol [Mass/volume] in Serum or Plasma (at least 30.00 ng/mL) |
|  |  |  | laboratory | UMLS:LNC:35365-6 | Vitamin D+Metabolites [Mass/volume] in Serum or Plasma (at least 30.00 ng/mL) |
|  |  |  | laboratory | TNX:LG25965-1 | Calcidiol+ercalcidiol [Mass/volume] in Serum, Plasma or Blood (at least 30.00 ng/mL) |

### Analyses Specifications

The Compare Outcomes Analytic supports four types of analyses: Measure of Association, Survival, Number of Instances, and Lab result distribution. The first three analyses support the “exclude patients with outcomes prior to the window” setting. This option can exclude patients from the analysis if they are not at risk for an outcome (e.g., if the outcome is a chronic disease). When "exclude patients with the outcome prior to the time window" is not checked, all patients in the cohort are included in the analysis, regardless of whether they had the outcome prior to the time window. When "exclude patients with the outcome prior to the time window" is checked, patients are excluded from the analysis if their record includes the outcome prior to the beginning of the time window. This selection will exclude all patients with the outcome prior to the index event. If the start of the time window for the analysis falls some days after the index event, patients will also be excluded if they have the outcome between the index event and the start of the time window.

### Measure of Association Analysis

The Measure of Association Analysis calculates and compares the fraction of patients with the selected outcome. The output summary includes: Patients in each Cohort (count of patients meeting query criteria); Patients with Outcome in each Cohort (of the patients in the cohort, count of patients that had the outcome in the time window); and Risk (the fraction of patients in the cohort that have the outcome in the time window, i.e. Patients with Outcome / Patients in Cohort). In addition, Risk Difference (the difference in the risks in Cohort 1 and Cohort 2), Risk Ratio (the ratio of the risks in Cohort 1 and Cohort 2), and Odds Ratio (the ratio of the odds in Cohort 1 and Cohort 2). The bar chart shows the risk of the outcome for the both cohorts.

### Survival Analysis

The Kaplan-Meier Analysis estimates probability of the outcome at a respective time interval (daily time interval is used in this analysis). In order to account for the patients who exited the cohort during the analysis period, and therefore should not be included in the analysis, censoring is applied. In this analysis, patients are removed from the analysis (censored) after the last fact in their record.

The output summary includes: Patients in each Cohort (count of patients meeting query criteria); Patients with Outcome (of the patients in the cohort, count of patients that had the outcome in the time window); Median Survival (the number of days when the survival drops below 50%; the “-” indicates that survival does not drop below 50% during the time window); and Survival Probability at End of Time Window (the % survival at the end of the time window). In addition, Log-Rank test, Hazard Ratio and test for Proportionality.

### Number of Instances Analysis

The Number of Instances Analysis calculates how many times the outcome occurred in the time window. This analysis includes two additional settings: include patients with zero instances; the definition of an instance.

Selecting to exclude patients with zero instances will remove these patients from the calculations for mean number of instances, standard deviation, or median. The histogram showing the distribution of patients by number of instances will not contain a bar for zero. Alternatively, by selecting to include patients with zero instances, the mean, standard deviation, and median for number of instances will reflect these patients. The histogram will contain a bar for zero patients.

The definition of an instance affects how counts are analyzed. By selecting Date, each calendar date on which any of the terms selected in the outcome are recorded will represent one instance. For example, if the outcome is “Med A or Med B,” and a patient has “Med A” on January 3, then both medications on January 4, then “Med B” on January 6, then that patient is considered to have three instances– January 3, January 4, and January 6. Note that if an outcome occurs across several dates (e.g. Visit: inpatient encounter), then only the start date is tracked for the purpose of counting instances. A patient who begins at stay on January 1, ends that stay on January 3, begins another stay on January 10, and ends that stay on January 15, is considered to have two instances of the outcome.

Selecting Visit as an Instance will count any visit that includes the outcome as one instance, regardless of how many times it occurred. For instance, consider a patient administered an analgesic on each of the three days that make up an inpatient stay following some index event. If analgesic is an outcome, these three administrations will represent only one instance, because all three are associated with the same visit.

The output summary includes: Patients in Cohort (count of patients meeting query criteria); Patients with Outcome (of the patients in the cohort, count of patients that had the outcome in the time window); Mean (mean of the counts); Standard Deviation (standard deviation of the counts); Median (median of the counts); and Median (1+ instances) when patients with zero instances included in the analysis. In addition, T-Test statistics testing for the difference between the cohorts is included.

### Laboratory Results Analysis

Lab Results can be included in the analysis only for the outcomes that are labs. Only the most recent lab values in the time window are included. For the lab results that are numeric, the outcome summary includes: Patients in Cohort (count of patients meeting query criteria); Patients with Outcome (of the patients in the cohort, count of patients that had the outcome in the time window); Mean (mean of the counts); and Standard Deviation (the standard deviation for lab values across patients in the cohort). In addition, T-Test statistics testing for the difference between the cohorts is included.

For the non-numeric lab results, three values are reported: counts of Negative; Positives; and Unknowns.

The counts are represented in the bar chart as percentages of the total counts.

### Outcome Definitions

Table below outlines the definitions for each outcome and the analysis specifications. For outcome definitions consisting of more than one term, at least one term must match. Please see Appendix C for the text representation of the outcome definitions.

| cognitive impairment | | | | |
| --- | --- | --- | --- | --- |
|  | **Outcome definition** | | | |
|  | | Diagnosis | UMLS:ICD10CM:G31.84 | Mild cognitive impairment of uncertain or unknown etiology |
|  | | Diagnosis | UMLS:ICD10CM:F09 | Unspecified mental disorder due to known physiological condition |
|  | **Settings for the performed analyses** | | | |
|  | | Risk analysis | | including patients with outcome prior to the time window |
|  | | Kaplan - Meier survival analysis | | including patients with outcome prior to the time window |
| depression | | | | |
|  | **Outcome definition** | | | |
|  | | Diagnosis | UMLS:ICD10CM:F32 | Depressive episode |
|  | **Settings for the performed analyses** | | | |
|  | | Risk analysis | | including patients with outcome prior to the time window |
|  | | Kaplan - Meier survival analysis | | including patients with outcome prior to the time window |
| dementia | | | | |
|  | **Outcome definition** | | | |
|  | | Diagnosis | UMLS:ICD10CM:F03 | Unspecified dementia |
|  | **Settings for the performed analyses** | | | |
|  | | Risk analysis | | including patients with outcome prior to the time window |
|  | | Kaplan - Meier survival analysis | | including patients with outcome prior to the time window |
| Unnamed Outcome | | | | |
|  | **Outcome definition** | | | |
|  | | Diagnosis | UMLS:ICD10CM:I63 | Cerebral infarction |
|  | **Settings for the performed analyses** | | | |
|  | | Kaplan - Meier survival analysis | | including patients with outcome prior to the time window |
|  | | Risk analysis | | including patients with outcome prior to the time window |

## Propensity Score Matching

Propensity score matching was performed on all listed characteristics. Characteristics of the cohorts before and after matching are summarized in the table below.

| **Cohort 1 and cohort 2 patient count before and after propensity score matching** | | | | | | | | | | | | |
| --- | --- | --- | --- | --- | --- | --- | --- | --- | --- | --- | --- | --- |
|  | | | Cohort | | | Patient count before matching | | | | Patient count after matching | | |
|  | | | 1 - ###VDD v2 (>50, depression | | | 19,614 | | | | 17,955 | | |
|  | | | 2 - ###control V2 (>50, depression) | | | 83,254 | | | | 17,955 | | |
| **Propensity score density function - Before and after matching (cohort 1 - purple, cohort 2 - green)** | | | | | | | | | | | | |
|  |  | | 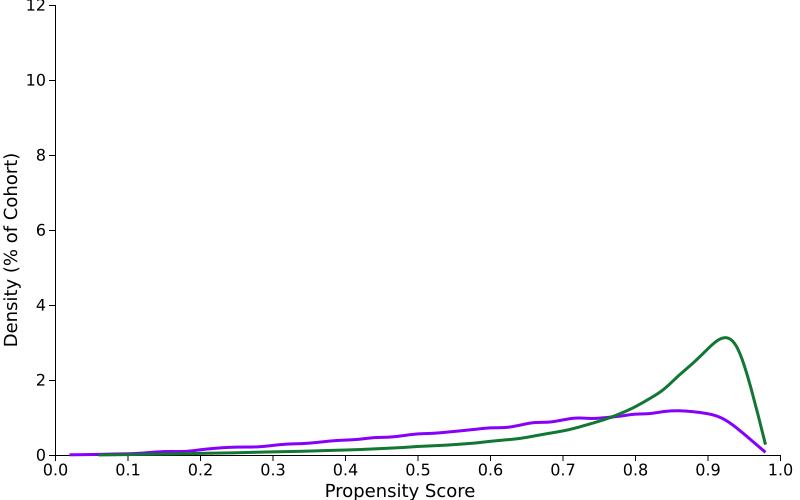 | | | | 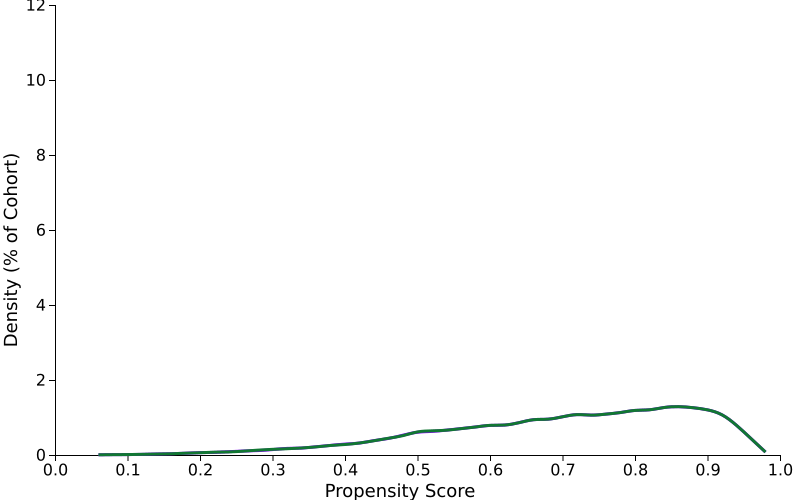 | | | | | |
| **Cohort 1 (N = 19,614) and cohort 2 (N = 83,254) characteristics before propensity score matching** | | | | | | | | | | | | |
|  | **Demographics** | | | | | | | | | | | |
|  |  | Cohort | | |  | Mean ± SD | | Patients | % of Cohort | | P-Value | Std diff. |
|  |  | 1 2 | | AI | Age at Index | 65.8 +/- 10.5 71.3 +/- 9.0 | | 19,447 83,252 | 100% 100% | | <0.001 | 0.568 |
|  |  | 1 2 | | 2106-3 | White |  | | 8,678 57,229 | 44.6% 68.7% | | <0.001 | 0.502 |
|  |  | 1 2 | | UNK | Unknown Race |  | | 3,712 8,438 | 19.1% 10.1% | | <0.001 | 0.256 |
|  |  | 1 2 | | 2054-5 | Black or African American |  | | 4,927 10,479 | 25.3% 12.6% | | <0.001 | 0.330 |
|  |  | 1 2 | | M | Male |  | | 10,140 35,919 | 52.1% 43.1% | | <0.001 | 0.181 |
|  | **Diagnosis** | | | | | | | | | | | |
|  |  | Cohort | | |  | Mean ± SD | | Patients | % of Cohort | | P-Value | Std diff. |
|  |  | 1 2 | | I10 | Essential (primary) hypertension |  | | 12,668 60,294 | 65.1% 72.4% | | <0.001 | 0.158 |
|  |  | 1 2 | | C00-D49 | Neoplasms |  | | 4,655 28,670 | 23.9% 34.4% | | <0.001 | 0.233 |
|  |  | 1 2 | | E66 | Overweight and obesity |  | | 5,206 19,405 | 26.8% 23.3% | | <0.001 | 0.080 |
|  |  | 1 2 | | E08-E13 | Diabetes mellitus |  | | 10,061 31,485 | 51.7% 37.8% | | <0.001 | 0.283 |
|  |  | 1 2 | | F17 | Nicotine dependence |  | | 0 0 | 0% 0% | | -- | -- |
|  |  | 1 2 | | I20-I25 | Ischemic heart diseases |  | | 5,282 19,116 | 27.2% 23.0% | | <0.001 | 0.097 |
|  |  | 1 2 | | F10 | Alcohol related disorders |  | | 0 0 | 0% 0% | | -- | -- |
|  |  | 1 2 | | I60-I69 | Cerebrovascular diseases |  | | 1,295 5,047 | 6.7% 6.1% | | 0.002 | 0.024 |
|  |  | 1 2 | | E40-E46 | Malnutrition |  | | 1,064 1,858 | 5.5% 2.2% | | <0.001 | 0.169 |
|  |  | 1 2 | | Z00-Z99 | Factors influencing health status and contact with health services |  | | 14,415 63,538 | 74.1% 76.3% | | <0.001 | 0.051 |
|  |  | 1 2 | | K76 | Other diseases of liver |  | | 1,380 5,416 | 7.1% 6.5% | | 0.003 | 0.023 |
|  |  | 1 2 | | E21 | Hyperparathyroidism and other disorders of parathyroid gland |  | | 771 4,053 | 4.0% 4.9% | | <0.001 | 0.044 |
|  |  | 1 2 | | N18.1 | Chronic kidney disease, stage 1 |  | | 376 1,841 | 1.9% 2.2% | | 0.016 | 0.020 |
|  |  | 1 2 | | N18.2 | Chronic kidney disease, stage 2 (mild) |  | | 1,708 9,939 | 8.8% 11.9% | | <0.001 | 0.104 |
|  |  | 1 2 | | N18.3 | Chronic kidney disease, stage 3 (moderate) |  | | 8,222 48,775 | 42.3% 58.6% | | <0.001 | 0.331 |
|  |  | 1 2 | | N18.4 | Chronic kidney disease, stage 4 (severe) |  | | 2,781 7,278 | 14.3% 8.7% | | <0.001 | 0.175 |
|  |  | 1 2 | | N18.5 | Chronic kidney disease, stage 5 |  | | 1,273 1,664 | 6.5% 2.0% | | <0.001 | 0.226 |
|  |  | 1 2 | | N18.6 | End stage renal disease |  | | 3,137 4,032 | 16.1% 4.8% | | <0.001 | 0.375 |
|  | **Laboratory** | | | | | | | | | | | |
|  |  | Cohort | | |  | Mean ± SD | | Patients | % of Cohort | | P-Value | Std diff. |
|  |  | 1 2 | | 9083 | BMI | 31.5 +/- 7.9 29.8 +/- 6.7 | | 12,135 58,913 | 62.4% 70.8% | | <0.001 | 0.232 |
|  |  | 1 2 | |  | 30 - 0 kg/m2 |  | | 7,548 30,790 | 38.8% 37.0% | | <0.001 | 0.038 |
|  |  | 1 2 | | 8001 | Glomerular filtration rate/1.73 sq M.predicted [Volume Rate/Area] in Serum, Plasma or Blood by Creatinine-based formula (MDRD) | 37.6 +/- 23.7 46.5 +/- 17.6 | | 16,831 72,872 | 86.5% 87.5% | | <0.001 | 0.430 |
|  |  | 1 2 | |  | 60 - 0 mL/min/{1.73_m2} |  | | 7,039 36,190 | 36.2% 43.5% | | <0.001 | 0.149 |
|  |  | 1 2 | | 9045 | Albumin [Mass/volume] in Serum, Plasma or Blood | 3.6 +/- 0.7 4.0 +/- 0.5 | | 15,489 67,551 | 79.6% 81.1% | | <0.001 | 0.692 |
|  |  | 1 2 | |  | 3.50 - 0 g/dL |  | | 12,655 65,227 | 65.1% 78.3% | | <0.001 | 0.298 |
|  |  | 1 2 | | 9063 | C reactive protein [Mass/volume] in Serum, Plasma or Blood | 44.0 +/- 67.7 25.3 +/- 49.9 | | 4,412 12,295 | 22.7% 14.8% | | <0.001 | 0.315 |
|  |  | 1 2 | |  | 5 - 0 mg/L |  | | 3,626 7,810 | 18.6% 9.4% | | <0.001 | 0.269 |
|  |  | 1 2 | | 9014 | Hemoglobin [Mass/volume] in Blood | 11.2 +/- 2.5 12.7 +/- 2.0 | | 16,396 68,171 | 84.3% 81.9% | | <0.001 | 0.626 |
|  |  | 1 2 | |  | 10 - 0 g/dL |  | | 14,887 66,570 | 76.6% 80.0% | | <0.001 | 0.083 |
| **Cohort 1 (N = 17,955) and cohort 2 (N = 17,955) characteristics after propensity score matching** | | | | | | | | | | | | |
|  | **Demographics** | | | | | | | | | | | |
|  |  | Cohort | | |  | Mean ± SD | | Patients | % of Cohort | | P-Value | Std diff. |
|  |  | 1 2 | | AI | Age at Index | 66.5 +/- 10.3 66.7 +/- 9.3 | | 17,955 17,955 | 100% 100% | | 0.134 | 0.016 |
|  |  | 1 2 | | 2106-3 | White |  | | 8,511 8,428 | 47.4% 46.9% | | 0.380 | 0.009 |
|  |  | 1 2 | | UNK | Unknown Race |  | | 3,227 3,228 | 18.0% 18.0% | | 0.989 | <0.001 |
|  |  | 1 2 | | 2054-5 | Black or African American |  | | 4,282 4,405 | 23.8% 24.5% | | 0.130 | 0.016 |
|  |  | 1 2 | | M | Male |  | | 9,137 9,159 | 50.9% 51.0% | | 0.816 | 0.002 |
|  | **Diagnosis** | | | | | | | | | | | |
|  |  | Cohort | | |  | Mean ± SD | | Patients | % of Cohort | | P-Value | Std diff. |
|  |  | 1 2 | | I10 | Essential (primary) hypertension |  | | 11,832 11,876 | 65.9% 66.1% | | 0.624 | 0.005 |
|  |  | 1 2 | | C00-D49 | Neoplasms |  | | 4,494 4,474 | 25.0% 24.9% | | 0.807 | 0.003 |
|  |  | 1 2 | | E66 | Overweight and obesity |  | | 4,750 4,772 | 26.5% 26.6% | | 0.793 | 0.003 |
|  |  | 1 2 | | E08-E13 | Diabetes mellitus |  | | 9,009 8,967 | 50.2% 49.9% | | 0.658 | 0.005 |
|  |  | 1 2 | | F17 | Nicotine dependence |  | | 0 0 | 0% 0% | | -- | -- |
|  |  | 1 2 | | I20-I25 | Ischemic heart diseases |  | | 4,737 4,724 | 26.4% 26.3% | | 0.876 | 0.002 |
|  |  | 1 2 | | F10 | Alcohol related disorders |  | | 0 0 | 0% 0% | | -- | -- |
|  |  | 1 2 | | I60-I69 | Cerebrovascular diseases |  | | 1,174 1,100 | 6.5% 6.1% | | 0.109 | 0.017 |
|  |  | 1 2 | | E40-E46 | Malnutrition |  | | 841 884 | 4.7% 4.9% | | 0.289 | 0.011 |
|  |  | 1 2 | | Z00-Z99 | Factors influencing health status and contact with health services |  | | 13,274 13,225 | 73.9% 73.7% | | 0.557 | 0.006 |
|  |  | 1 2 | | K76 | Other diseases of liver |  | | 1,264 1,191 | 7.0% 6.6% | | 0.127 | 0.016 |
|  |  | 1 2 | | E21 | Hyperparathyroidism and other disorders of parathyroid gland |  | | 703 641 | 3.9% 3.6% | | 0.085 | 0.018 |
|  |  | 1 2 | | N18.1 | Chronic kidney disease, stage 1 |  | | 366 356 | 2.0% 2.0% | | 0.707 | 0.004 |
|  |  | 1 2 | | N18.2 | Chronic kidney disease, stage 2 (mild) |  | | 1,681 1,643 | 9.4% 9.2% | | 0.489 | 0.007 |
|  |  | 1 2 | | N18.3 | Chronic kidney disease, stage 3 (moderate) |  | | 7,964 7,947 | 44.4% 44.3% | | 0.857 | 0.002 |
|  |  | 1 2 | | N18.4 | Chronic kidney disease, stage 4 (severe) |  | | 2,412 2,466 | 13.4% 13.7% | | 0.406 | 0.009 |
|  |  | 1 2 | | N18.5 | Chronic kidney disease, stage 5 |  | | 974 994 | 5.4% 5.5% | | 0.643 | 0.005 |
|  |  | 1 2 | | N18.6 | End stage renal disease |  | | 2,397 2,446 | 13.4% 13.6% | | 0.449 | 0.008 |
|  | **Laboratory** | | | | | | | | | | | |
|  |  | Cohort | | |  | Mean ± SD | | Patients | % of Cohort | | P-Value | Std diff. |
|  |  | 1 2 | | 9083 | BMI | 31.5 +/- 7.9 30.8 +/- 7.3 | | 11,233 11,944 | 62.6% 66.5% | | <0.001 | 0.096 |
|  |  | 1 2 | |  | 30 - 0 kg/m2 |  | | 6,970 7,040 | 38.8% 39.2% | | 0.449 | 0.008 |
|  |  | 1 2 | | 8001 | Glomerular filtration rate/1.73 sq M.predicted [Volume Rate/Area] in Serum, Plasma or Blood by Creatinine-based formula (MDRD) | 38.7 +/- 23.4 42.9 +/- 21.1 | | 15,514 14,670 | 86.4% 81.7% | | <0.001 | 0.190 |
|  |  | 1 2 | |  | 60 - 0 mL/min/{1.73_m2} |  | | 6,714 6,660 | 37.4% 37.1% | | 0.556 | 0.006 |
|  |  | 1 2 | | 9045 | Albumin [Mass/volume] in Serum, Plasma or Blood | 3.6 +/- 0.7 3.9 +/- 0.6 | | 14,314 13,269 | 79.7% 73.9% | | <0.001 | 0.428 |
|  |  | 1 2 | |  | 3.50 - 0 g/dL |  | | 12,049 12,119 | 67.1% 67.5% | | 0.431 | 0.008 |
|  |  | 1 2 | | 9063 | C reactive protein [Mass/volume] in Serum, Plasma or Blood | 40.7 +/- 64.2 34.2 +/- 56.8 | | 3,756 3,730 | 20.9% 20.8% | | <0.001 | 0.107 |
|  |  | 1 2 | |  | 5 - 0 mg/L |  | | 3,000 2,988 | 16.7% 16.6% | | 0.865 | 0.002 |
|  |  | 1 2 | | 9014 | Hemoglobin [Mass/volume] in Blood | 11.3 +/- 2.5 12.2 +/- 2.2 | | 15,044 14,312 | 83.8% 79.7% | | <0.001 | 0.363 |
|  |  | 1 2 | |  | 10 - 0 g/dL |  | | 13,744 13,748 | 76.5% 76.6% | | 0.960 | 0.001 |

# Results

Results are summarized in the tables below. Outcomes analysis was performed on the cohorts after propensity score matching.

| **1 cognitive impairment** | | | | | | | | | | | | |
| --- | --- | --- | --- | --- | --- | --- | --- | --- | --- | --- | --- | --- |
|  | | **Risk analysis** | | | | | | | | | | |
|  |  | | | Cohort | | | Patients in cohort | Patients with outcome | Risk | | | |
|  | | |  | 1 | | ###VDD v2 (>50, depression | 17,955 | 28 | 0.002 | | | |
|  | | |  | 2 | | ###control V2 (>50, depression) | 17,955 | 20 | 0.001 | | | |
|  | | | | | | | | | | | | |
|  | | |  |  | | |  | 95% CI | z | p |  |  |
|  | | |  | **Risk Difference** | | | 0.000 | (-0.000, 0.001) | 1.155 | 0.248 |  |  |
|  | | |  | **Risk Ratio** | | | 1.400 | (0.789, 2.484) | N/A | N/A |  |  |
|  | | |  | **Odds Ratio** | | | 1.401 | (0.789, 2.487) | N/A | N/A |  |  |
|  | | | | | | | | | | | | |
|  | |  | | | 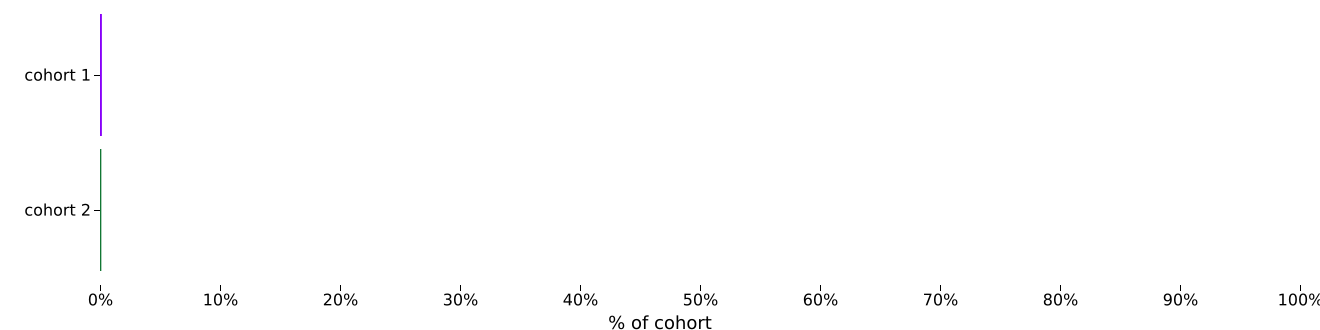 | | | | | | | |
|  | | **Kaplan - Meier survival analysis** | | | | | | | | | | |
|  | | |  | Cohort | | | Patients in cohort | Patients with outcome | Median survival (days) | Survival probability at end of time window | | |
|  | | |  | 1 | | ###VDD v2 (>50, depression | 17,955 | 28 | -- | 99.82% | | |
|  | | |  | 2 | | ###control V2 (>50, depression) | 17,955 | 20 | -- | 99.88% | | |
|  | | | | | | | | | | | | |
|  | | |  |  | | | χ^2^ | df | p |  |  |  |
|  | | |  | **Log-Rank Test** | | | 1.747 | 1 | 0.186 |  |  |  |
|  | | | | | | | | | | | | |
|  | | |  |  | | | Hazard Ratio | 95% CI | χ^2^ | df | p | |
|  | | |  | **Hazard Ratio and Proportionality** | | | 1.469 | (0.828, 2.608) | 0.010 | 1 | 0.918 | |
|  | | | | | | | | | | | | |
|  | |  | | | 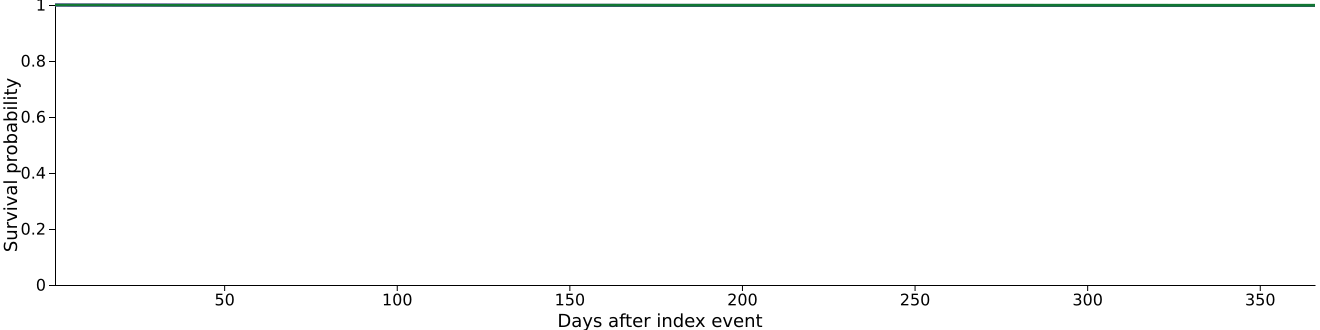 | | | | | | | |
| **2 depression** | | | | | | | | | | | | |
|  | | **Risk analysis** | | | | | | | | | | |
|  |  | | | Cohort | | | Patients in cohort | Patients with outcome | Risk | | | |
|  | | |  | 1 | | ###VDD v2 (>50, depression | 17,955 | 191 | 0.011 | | | |
|  | | |  | 2 | | ###control V2 (>50, depression) | 17,955 | 105 | 0.006 | | | |
|  | | | | | | | | | | | | |
|  | | |  |  | | |  | 95% CI | z | p |  |  |
|  | | |  | **Risk Difference** | | | 0.005 | (0.003, 0.007) | 5.019 | 0.000 |  |  |
|  | | |  | **Risk Ratio** | | | 1.819 | (1.435, 2.306) | N/A | N/A |  |  |
|  | | |  | **Odds Ratio** | | | 1.828 | (1.439, 2.321) | N/A | N/A |  |  |
|  | | | | | | | | | | | | |
|  | |  | | | 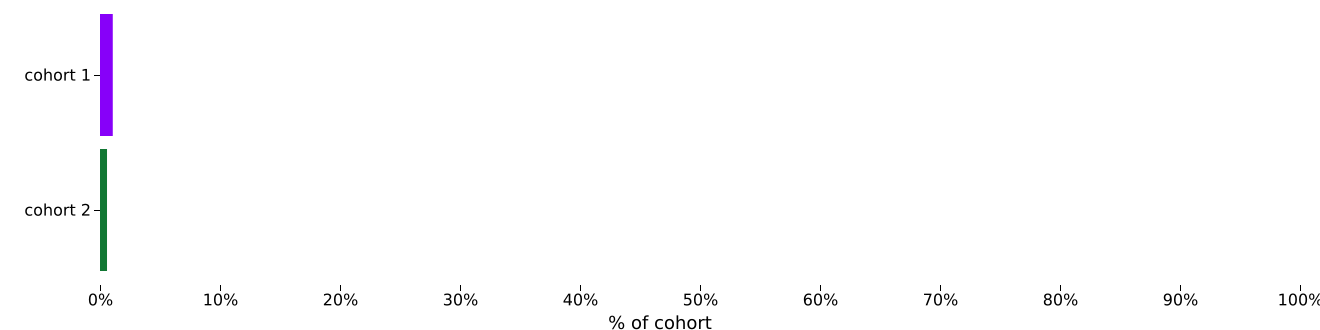 | | | | | | | |
|  | | **Kaplan - Meier survival analysis** | | | | | | | | | | |
|  | | |  | Cohort | | | Patients in cohort | Patients with outcome | Median survival (days) | Survival probability at end of time window | | |
|  | | |  | 1 | | ###VDD v2 (>50, depression | 17,955 | 191 | -- | 98.73% | | |
|  | | |  | 2 | | ###control V2 (>50, depression) | 17,955 | 105 | -- | 99.33% | | |
|  | | | | | | | | | | | | |
|  | | |  |  | | | χ^2^ | df | p |  |  |  |
|  | | |  | **Log-Rank Test** | | | 30.318 | 1 | 0.000 |  |  |  |
|  | | | | | | | | | | | | |
|  | | |  |  | | | Hazard Ratio | 95% CI | χ^2^ | df | p | |
|  | | |  | **Hazard Ratio and Proportionality** | | | 1.929 | (1.520, 2.448) | 4.138 | 1 | 0.042 | |
|  | | | | | | | | | | | | |
|  | |  | | | 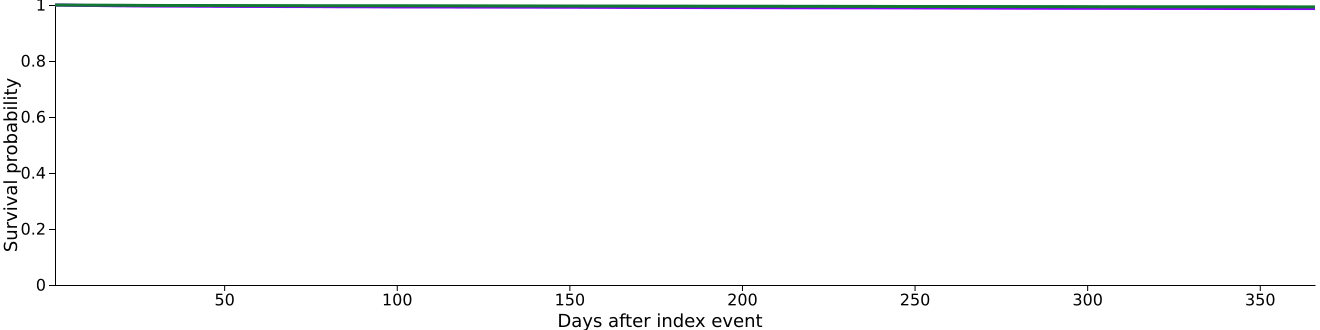 | | | | | | | |
| **3 dementia** | | | | | | | | | | | | |
|  | | **Risk analysis** | | | | | | | | | | |
|  |  | | | Cohort | | | Patients in cohort | Patients with outcome | Risk | | | |
|  | | |  | 1 | | ###VDD v2 (>50, depression | 17,955 | 73 | 0.004 | | | |
|  | | |  | 2 | | ###control V2 (>50, depression) | 17,955 | 25 | 0.001 | | | |
|  | | | | | | | | | | | | |
|  | | |  |  | | |  | 95% CI | z | p |  |  |
|  | | |  | **Risk Difference** | | | 0.003 | (0.002, 0.004) | 4.855 | 0.000 |  |  |
|  | | |  | **Risk Ratio** | | | 2.920 | (1.855, 4.597) | N/A | N/A |  |  |
|  | | |  | **Odds Ratio** | | | 2.928 | (1.858, 4.613) | N/A | N/A |  |  |
|  | | | | | | | | | | | | |
|  | |  | | | 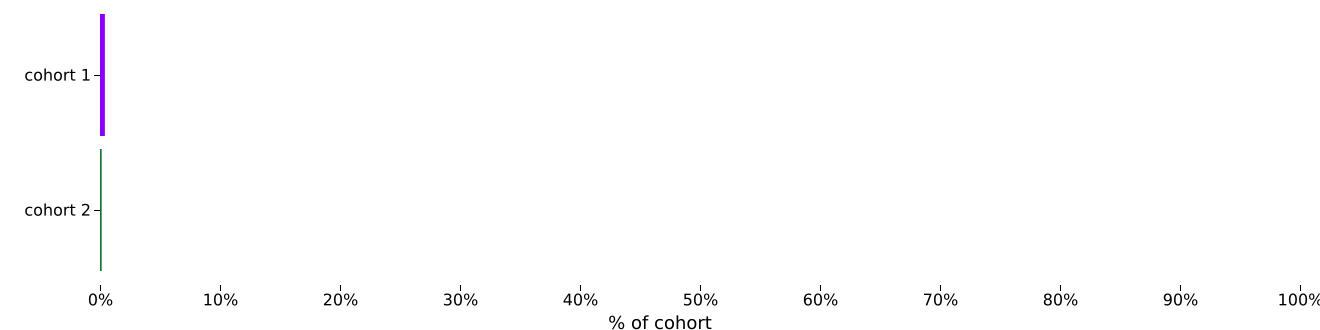 | | | | | | | |
|  | | **Kaplan - Meier survival analysis** | | | | | | | | | | |
|  | | |  | Cohort | | | Patients in cohort | Patients with outcome | Median survival (days) | Survival probability at end of time window | | |
|  | | |  | 1 | | ###VDD v2 (>50, depression | 17,955 | 73 | -- | 99.52% | | |
|  | | |  | 2 | | ###control V2 (>50, depression) | 17,955 | 25 | -- | 99.84% | | |
|  | | | | | | | | | | | | |
|  | | |  |  | | | χ^2^ | df | p |  |  |  |
|  | | |  | **Log-Rank Test** | | | 26.066 | 1 | 0.000 |  |  |  |
|  | | | | | | | | | | | | |
|  | | |  |  | | | Hazard Ratio | 95% CI | χ^2^ | df | p | |
|  | | |  | **Hazard Ratio and Proportionality** | | | 3.075 | (1.952, 4.843) | 1.462 | 1 | 0.227 | |
|  | | | | | | | | | | | | |
|  | |  | | | 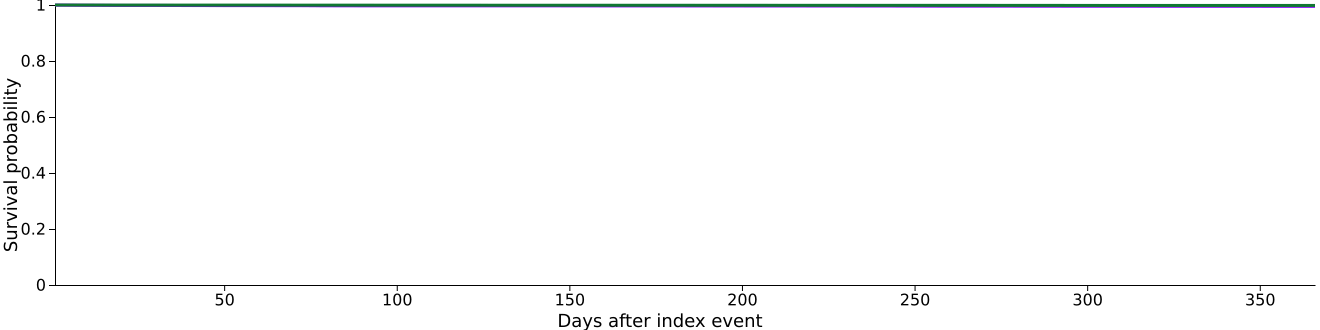 | | | | | | | |
| **4 Unnamed Outcome** | | | | | | | | | | | | |
|  | | **Risk analysis** | | | | | | | | | | |
|  |  | | | Cohort | | | Patients in cohort | Patients with outcome | Risk | | | |
|  | | |  | 1 | | ###VDD v2 (>50, depression | 17,955 | 130 | 0.007 | | | |
|  | | |  | 2 | | ###control V2 (>50, depression) | 17,955 | 61 | 0.003 | | | |
|  | | | | | | | | | | | | |
|  | | |  |  | | |  | 95% CI | z | p |  |  |
|  | | |  | **Risk Difference** | | | 0.004 | (0.002, 0.005) | 5.006 | 0.000 |  |  |
|  | | |  | **Risk Ratio** | | | 2.131 | (1.573, 2.887) | N/A | N/A |  |  |
|  | | |  | **Odds Ratio** | | | 2.139 | (1.577, 2.902) | N/A | N/A |  |  |
|  | | | | | | | | | | | | |
|  | |  | | | 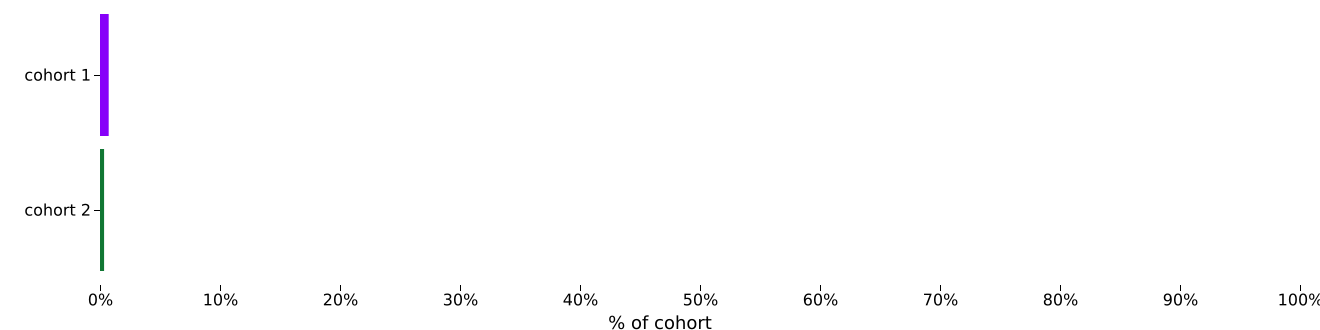 | | | | | | | |
|  | | **Kaplan - Meier survival analysis** | | | | | | | | | | |
|  | | |  | Cohort | | | Patients in cohort | Patients with outcome | Median survival (days) | Survival probability at end of time window | | |
|  | | |  | 1 | | ###VDD v2 (>50, depression | 17,955 | 130 | -- | 99.18% | | |
|  | | |  | 2 | | ###control V2 (>50, depression) | 17,955 | 61 | -- | 99.63% | | |
|  | | | | | | | | | | | | |
|  | | |  |  | | | χ^2^ | df | p |  |  |  |
|  | | |  | **Log-Rank Test** | | | 27.758 | 1 | 0.000 |  |  |  |
|  | | | | | | | | | | | | |
|  | | |  |  | | | Hazard Ratio | 95% CI | χ^2^ | df | p | |
|  | | |  | **Hazard Ratio and Proportionality** | | | 2.218 | (1.636, 3.006) | 0.000 | 1 | 0.997 | |
|  | | | | | | | | | | | | |
|  | |  | | | 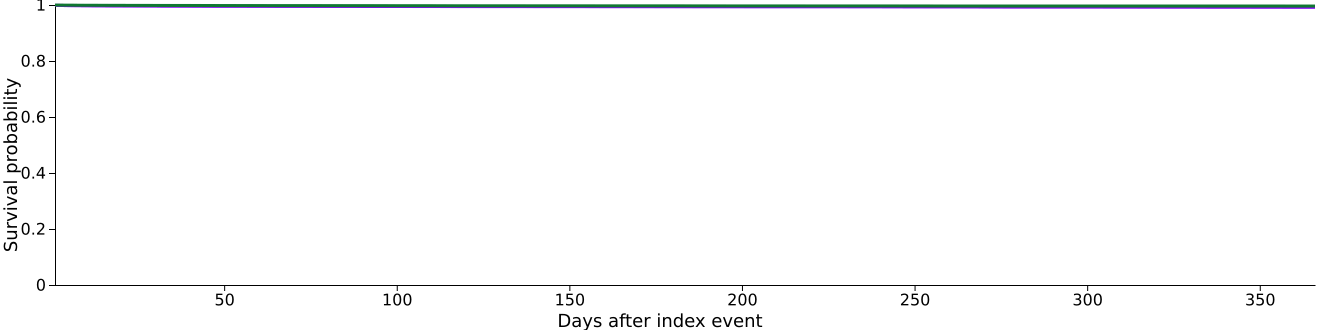 | | | | | | | |

# Appendix A – Text Representation of the Cohorts Definition

This section lists all terms used in the definitions of the two cohorts.

### Query Criteria for Cohort 1 (query name: ###VDD v2 (>50, depression)

Patients must have:
 Age (Age) (at least 50 years (most recent occurrence)).

All the following must be satisfied:

 Visit>2: The terms in this group occurred between Jan 1, 2010 and Dec 31, 2019 (Greater than or equal to 2 instances)
 Patients must have:
 Visit (TNX:Visit).


 CKD history: The terms in this group occurred at any time
 Patients must have:
 any of the following:
 Chronic kidney disease (CKD) (UMLS:ICD10CM:N18) (at least 18 years old at event); or
 Chronic kidney disease, unspecified (UMLS:ICD10CM:N18.9) (at least 18 years old at event); or
 Chronic kidney disease, stage 3 (moderate) (UMLS:ICD10CM:N18.3) (at least 18 years old at event); or
 Chronic kidney disease, stage 3 unspecified (UMLS:ICD10CM:N18.30) (at least 18 years old at event); or
 Chronic kidney disease, stage 4 (severe) (UMLS:ICD10CM:N18.4) (at least 18 years old at event); or
 Chronic kidney disease, stage 2 (mild) (UMLS:ICD10CM:N18.2) (at least 18 years old at event); or
 Chronic kidney disease, stage 3a (UMLS:ICD10CM:N18.31) (at least 18 years old at event); or
 Chronic kidney disease, stage 3b (UMLS:ICD10CM:N18.32) (at least 18 years old at event); or
 Chronic kidney disease, stage 5 (UMLS:ICD10CM:N18.5) (at least 18 years old at event); or
 Chronic kidney disease, stage 1 (UMLS:ICD10CM:N18.1) (at least 18 years old at event); or
 End stage renal disease (UMLS:ICD10CM:N18.6) (at least 18 years old at event).
 CKD三個月內vitamin D<20: Any instance of CKD三個月內vitamin D<20 occurred within 3 months on or after any instance of CKD history
 Patients must have:
 any of the following:
 Vitamin D+Metabolites [Mass/volume] in Serum or Plasma (UMLS:LNC:35365-6) (at most 20.00 ng/mL); or
 Calcidiol+ercalcidiol [Mass/volume] in Serum, Plasma or Blood (TNX:LG25965-1) (at most 20.00 ng/mL); or
 Calcidiol [Mass/volume] in Serum or Plasma (TNX:9034) (at most 20.00 ng/mL).


 CKD history: The terms in this group occurred at any time
 Patients must have:
 any of the following:
 Chronic kidney disease (CKD) (UMLS:ICD10CM:N18) (at least 50 years old at event); or
 Chronic kidney disease, unspecified (UMLS:ICD10CM:N18.9) (at least 50 years old at event); or
 Chronic kidney disease, stage 3 (moderate) (UMLS:ICD10CM:N18.3) (at least 50 years old at event); or
 Chronic kidney disease, stage 3 unspecified (UMLS:ICD10CM:N18.30) (at least 50 years old at event); or
 Chronic kidney disease, stage 4 (severe) (UMLS:ICD10CM:N18.4) (at least 50 years old at event); or
 Chronic kidney disease, stage 2 (mild) (UMLS:ICD10CM:N18.2) (at least 50 years old at event); or
 Chronic kidney disease, stage 3a (UMLS:ICD10CM:N18.31) (at least 50 years old at event); or
 Chronic kidney disease, stage 3b (UMLS:ICD10CM:N18.32) (at least 50 years old at event); or
 Chronic kidney disease, stage 5 (UMLS:ICD10CM:N18.5) (at least 50 years old at event); or
 Chronic kidney disease, stage 1 (UMLS:ICD10CM:N18.1) (at least 50 years old at event); or
 End stage renal disease (UMLS:ICD10CM:N18.6) (at least 50 years old at event).
 在CKD三個月內排除vitamin D>21: Any instance of 在CKD三個月內排除vitamin D>21 occurred within 3 months on or after any instance of CKD history
 Patients cannot have:
 any of the following:
 Calcidiol+ercalcidiol [Mass/volume] in Serum, Plasma or Blood (TNX:LG25965-1) (at least 21.00 ng/mL); or
 Calcidiol [Mass/volume] in Serum or Plasma (TNX:9034) (at least 21.00 ng/mL); or
 Vitamin D+Metabolites [Mass/volume] in Serum or Plasma (UMLS:LNC:35365-6) (at least 21.00 ng/mL).


 CKD history: The terms in this group occurred at any time
 Patients must have:
 any of the following:
 Chronic kidney disease (CKD) (UMLS:ICD10CM:N18) (at least 18 years old at event); or
 Chronic kidney disease, unspecified (UMLS:ICD10CM:N18.9) (at least 18 years old at event); or
 Chronic kidney disease, stage 3 (moderate) (UMLS:ICD10CM:N18.3) (at least 18 years old at event); or
 Chronic kidney disease, stage 3 unspecified (UMLS:ICD10CM:N18.30) (at least 18 years old at event); or
 Chronic kidney disease, stage 4 (severe) (UMLS:ICD10CM:N18.4) (at least 18 years old at event); or
 Chronic kidney disease, stage 2 (mild) (UMLS:ICD10CM:N18.2) (at least 18 years old at event); or
 Chronic kidney disease, stage 3a (UMLS:ICD10CM:N18.31) (at least 18 years old at event); or
 Chronic kidney disease, stage 3b (UMLS:ICD10CM:N18.32) (at least 18 years old at event); or
 Chronic kidney disease, stage 5 (UMLS:ICD10CM:N18.5) (at least 18 years old at event); or
 Chronic kidney disease, stage 1 (UMLS:ICD10CM:N18.1) (at least 18 years old at event); or
 End stage renal disease (UMLS:ICD10CM:N18.6) (at least 18 years old at event).
 排除cognitive impairment: Any instance of 排除cognitive impairment occurred at least 1 day before any instance of CKD history
 Patients cannot have:
 any of the following:
 Human immunodeficiency virus [HIV] disease (UMLS:ICD10CM:B20); or
 Mild cognitive impairment of uncertain or unknown etiology (UMLS:ICD10CM:G31.84); or
 Other mental disorders due to known physiological condition (UMLS:ICD10CM:F06); or
 Other specified mental disorders due to known physiological condition (UMLS:ICD10CM:F06.8); or
 Other disorders of psychological development (UMLS:ICD10CM:F88); or
 Individual Psychotherapy, Cognitive (UMLS:ICD10PCS:GZ52ZZZ); or
 Mild neurocognitive disorder due to known physiological condition (UMLS:ICD10CM:F06.7); or
 Unspecified mental disorder due to known physiological condition (UMLS:ICD10CM:F09).


 CKD history: The terms in this group occurred at any time
 Patients must have:
 any of the following:
 Chronic kidney disease (CKD) (UMLS:ICD10CM:N18) (at least 18 years old at event); or
 Chronic kidney disease, unspecified (UMLS:ICD10CM:N18.9) (at least 18 years old at event); or
 Chronic kidney disease, stage 3 (moderate) (UMLS:ICD10CM:N18.3) (at least 18 years old at event); or
 Chronic kidney disease, stage 3 unspecified (UMLS:ICD10CM:N18.30) (at least 18 years old at event); or
 Chronic kidney disease, stage 4 (severe) (UMLS:ICD10CM:N18.4) (at least 18 years old at event); or
 Chronic kidney disease, stage 2 (mild) (UMLS:ICD10CM:N18.2) (at least 18 years old at event); or
 Chronic kidney disease, stage 3a (UMLS:ICD10CM:N18.31) (at least 18 years old at event); or
 Chronic kidney disease, stage 3b (UMLS:ICD10CM:N18.32) (at least 18 years old at event); or
 Chronic kidney disease, stage 5 (UMLS:ICD10CM:N18.5) (at least 18 years old at event); or
 Chronic kidney disease, stage 1 (UMLS:ICD10CM:N18.1) (at least 18 years old at event); or
 End stage renal disease (UMLS:ICD10CM:N18.6) (at least 18 years old at event).
 排除dementia，Alzheimer's disease, Parkinsom disease: Any instance of 排除dementia，Alzheimer's disease, Parkinsom disease occurred at least 1 day before any instance of CKD history
 Patients cannot have:
 any of the following:
 Unspecified dementia (UMLS:ICD10CM:F03); or
 Unspecified dementia, unspecified severity, without behavioral disturbance, psychotic disturbance, mood disturbance, and anxiety (UMLS:ICD10CM:F03.90); or
 Dementia in other diseases classified elsewhere, unspecified severity, without behavioral disturbance, psychotic disturbance, mood disturbance, and anxiety (UMLS:ICD10CM:F02.80); or
 Psychotic disorder with hallucinations due to known physiological condition (UMLS:ICD10CM:F06.0); or
 Alzheimer's disease (UMLS:ICD10CM:G30); or
 Parkinson's disease (UMLS:ICD10CM:G20); or
 Delirium due to known physiological condition (UMLS:ICD10CM:F05); or
 Alzheimer's disease (UMLS:ICD10CM:G30); or
 Dementia in other diseases classified elsewhere, unspecified severity, without behavioral disturbance, psychotic disturbance, mood disturbance, and anxiety (UMLS:ICD10CM:F02.80); or
 Alzheimer's disease, unspecified (UMLS:ICD10CM:G30.9); or
 Other Alzheimer's disease (UMLS:ICD10CM:G30.8); or
 Delirium due to known physiological condition (UMLS:ICD10CM:F05); or
 Psychotic disorder with delusions due to known physiological condition (UMLS:ICD10CM:F06.2); or
 Alzheimer's disease with late onset (UMLS:ICD10CM:G30.1); or
 Alzheimer's disease with early onset (UMLS:ICD10CM:G30.0); or
 Alzheimer's disease with late onset (UMLS:ICD10CM:G30.1); or
 Other Alzheimer's disease (UMLS:ICD10CM:G30.8); or
 Alzheimer's disease, unspecified (UMLS:ICD10CM:G30.9); or
 Dementia in other diseases classified elsewhere, mild (UMLS:ICD10CM:F02.A); or
 Dementia in other diseases classified elsewhere, severe (UMLS:ICD10CM:F02.C); or
 Dementia in other diseases classified elsewhere, mild, without behavioral disturbance, psychotic disturbance, mood disturbance, and anxiety (UMLS:ICD10CM:F02.A0); or
 Dementia in other diseases classified elsewhere, moderate, without behavioral disturbance, psychotic disturbance, mood disturbance, and anxiety (UMLS:ICD10CM:F02.B0); or
 Dementia in other diseases classified elsewhere, unspecified severity, with mood disturbance (UMLS:ICD10CM:F02.83); or
 Dementia in other diseases classified elsewhere, unspecified severity, with agitation (UMLS:ICD10CM:F02.811); or
 Dementia in other diseases classified elsewhere, moderate (UMLS:ICD10CM:F02.B); or
 Dementia in other diseases classified elsewhere, unspecified severity, with other behavioral disturbance (UMLS:ICD10CM:F02.818); or
 Dementia in other diseases classified elsewhere, unspecified severity, with behavioral disturbance (UMLS:ICD10CM:F02.81); or
 Unspecified dementia (UMLS:ICD10CM:F03); or
 Unspecified dementia, unspecified severity, without behavioral disturbance, psychotic disturbance, mood disturbance, and anxiety (UMLS:ICD10CM:F03.90); or
 Dementia in other diseases classified elsewhere, unspecified severity, without behavioral disturbance, psychotic disturbance, mood disturbance, and anxiety (UMLS:ICD10CM:F02.80); or
 Psychotic disorder with hallucinations due to known physiological condition (UMLS:ICD10CM:F06.0); or
 Dementia in other diseases classified elsewhere (UMLS:ICD10CM:F02); or
 Dementia in other diseases classified elsewhere, unspecified severity (UMLS:ICD10CM:F02.8).


 排除renal transplanation: The terms in this group occurred at any time
 Patients cannot have:
 Renal Transplantation Procedures (UMLS:CPT:1008098).


 CKD history: The terms in this group occurred at any time
 Patients must have:
 any of the following:
 Chronic kidney disease (CKD) (UMLS:ICD10CM:N18) (at least 18 years old at event); or
 Chronic kidney disease, unspecified (UMLS:ICD10CM:N18.9) (at least 18 years old at event); or
 Chronic kidney disease, stage 3 (moderate) (UMLS:ICD10CM:N18.3) (at least 18 years old at event); or
 Chronic kidney disease, stage 3 unspecified (UMLS:ICD10CM:N18.30) (at least 18 years old at event); or
 Chronic kidney disease, stage 4 (severe) (UMLS:ICD10CM:N18.4) (at least 18 years old at event); or
 Chronic kidney disease, stage 2 (mild) (UMLS:ICD10CM:N18.2) (at least 18 years old at event); or
 Chronic kidney disease, stage 3a (UMLS:ICD10CM:N18.31) (at least 18 years old at event); or
 Chronic kidney disease, stage 3b (UMLS:ICD10CM:N18.32) (at least 18 years old at event); or
 Chronic kidney disease, stage 5 (UMLS:ICD10CM:N18.5) (at least 18 years old at event); or
 Chronic kidney disease, stage 1 (UMLS:ICD10CM:N18.1) (at least 18 years old at event); or
 End stage renal disease (UMLS:ICD10CM:N18.6) (at least 18 years old at event).
 排除vascular dementia: Any instance of 排除vascular dementia occurred at least 1 day before any instance of CKD history
 Patients cannot have:
 any of the following:
 Vascular dementia (UMLS:ICD10CM:F01); or
 Cerebral atherosclerosis (UMLS:ICD10CM:I67.2); or
 Vascular dementia, unspecified severity, without behavioral disturbance, psychotic disturbance, mood disturbance, and anxiety (UMLS:ICD10CM:F01.50); or
 Vascular dementia, unspecified severity (UMLS:ICD10CM:F01.5); or
 Vascular dementia, mild (UMLS:ICD10CM:F01.A); or
 Vascular dementia, moderate (UMLS:ICD10CM:F01.B); or
 Vascular dementia, mild, without behavioral disturbance, psychotic disturbance, mood disturbance, and anxiety (UMLS:ICD10CM:F01.A0); or
 Vascular dementia, moderate, without behavioral disturbance, psychotic disturbance, mood disturbance, and anxiety (UMLS:ICD10CM:F01.B0); or
 Vascular dementia, severe (UMLS:ICD10CM:F01.C).


 CKD history: The terms in this group occurred at any time
 Patients must have:
 any of the following:
 Chronic kidney disease (CKD) (UMLS:ICD10CM:N18) (at least 18 years old at event); or
 Chronic kidney disease, unspecified (UMLS:ICD10CM:N18.9) (at least 18 years old at event); or
 Chronic kidney disease, stage 3 (moderate) (UMLS:ICD10CM:N18.3) (at least 18 years old at event); or
 Chronic kidney disease, stage 3 unspecified (UMLS:ICD10CM:N18.30) (at least 18 years old at event); or
 Chronic kidney disease, stage 4 (severe) (UMLS:ICD10CM:N18.4) (at least 18 years old at event); or
 Chronic kidney disease, stage 2 (mild) (UMLS:ICD10CM:N18.2) (at least 18 years old at event); or
 Chronic kidney disease, stage 3a (UMLS:ICD10CM:N18.31) (at least 18 years old at event); or
 Chronic kidney disease, stage 3b (UMLS:ICD10CM:N18.32) (at least 18 years old at event); or
 Chronic kidney disease, stage 5 (UMLS:ICD10CM:N18.5) (at least 18 years old at event); or
 Chronic kidney disease, stage 1 (UMLS:ICD10CM:N18.1) (at least 18 years old at event); or
 End stage renal disease (UMLS:ICD10CM:N18.6) (at least 18 years old at event).
 排除Schizophrenia: Any instance of 排除Schizophrenia occurred at least 1 day before any instance of CKD history
 Patients cannot have:
 any of the following:
 Schizophrenia (UMLS:ICD10CM:F20); or
 Schizophrenia, schizotypal, delusional, and other non-mood psychotic disorders (UMLS:ICD10CM:F20-F29); or
 Schizophrenia, unspecified (UMLS:ICD10CM:F20.9); or
 Personal history of other specified conditions (UMLS:ICD10CM:Z87.898); or
 Pervasive developmental disorders (UMLS:ICD10CM:F84); or
 Unspecified psychosis not due to a substance or known physiological condition (UMLS:ICD10CM:F29).


 CKD history: The terms in this group occurred at any time
 Patients must have:
 any of the following:
 Chronic kidney disease (CKD) (UMLS:ICD10CM:N18) (at least 18 years old at event); or
 Chronic kidney disease, unspecified (UMLS:ICD10CM:N18.9) (at least 18 years old at event); or
 Chronic kidney disease, stage 3 (moderate) (UMLS:ICD10CM:N18.3) (at least 18 years old at event); or
 Chronic kidney disease, stage 3 unspecified (UMLS:ICD10CM:N18.30) (at least 18 years old at event); or
 Chronic kidney disease, stage 4 (severe) (UMLS:ICD10CM:N18.4) (at least 18 years old at event); or
 Chronic kidney disease, stage 2 (mild) (UMLS:ICD10CM:N18.2) (at least 18 years old at event); or
 Chronic kidney disease, stage 3a (UMLS:ICD10CM:N18.31) (at least 18 years old at event); or
 Chronic kidney disease, stage 3b (UMLS:ICD10CM:N18.32) (at least 18 years old at event); or
 Chronic kidney disease, stage 5 (UMLS:ICD10CM:N18.5) (at least 18 years old at event); or
 Chronic kidney disease, stage 1 (UMLS:ICD10CM:N18.1) (at least 18 years old at event); or
 End stage renal disease (UMLS:ICD10CM:N18.6) (at least 18 years old at event).
 排除biploar disorders: Any instance of 排除biploar disorders occurred at least 1 day before any instance of CKD history
 Patients cannot have:
 any of the following:
 Bipolar disorder (UMLS:ICD10CM:F31); or
 Personal history of other mental and behavioral disorders (UMLS:ICD10CM:Z86.59); or
 Bipolar II disorder (UMLS:ICD10CM:F31.81); or
 Bipolar disorder, current episode manic severe with psychotic features (UMLS:ICD10CM:F31.2); or
 Bipolar disorder, current episode mixed, unspecified (UMLS:ICD10CM:F31.60).


 CKD history: The terms in this group occurred at any time
 Patients must have:
 any of the following:
 Chronic kidney disease (CKD) (UMLS:ICD10CM:N18) (at least 18 years old at event); or
 Chronic kidney disease, unspecified (UMLS:ICD10CM:N18.9) (at least 18 years old at event); or
 Chronic kidney disease, stage 3 (moderate) (UMLS:ICD10CM:N18.3) (at least 18 years old at event); or
 Chronic kidney disease, stage 3 unspecified (UMLS:ICD10CM:N18.30) (at least 18 years old at event); or
 Chronic kidney disease, stage 4 (severe) (UMLS:ICD10CM:N18.4) (at least 18 years old at event); or
 Chronic kidney disease, stage 2 (mild) (UMLS:ICD10CM:N18.2) (at least 18 years old at event); or
 Chronic kidney disease, stage 3a (UMLS:ICD10CM:N18.31) (at least 18 years old at event); or
 Chronic kidney disease, stage 3b (UMLS:ICD10CM:N18.32) (at least 18 years old at event); or
 Chronic kidney disease, stage 5 (UMLS:ICD10CM:N18.5) (at least 18 years old at event); or
 Chronic kidney disease, stage 1 (UMLS:ICD10CM:N18.1) (at least 18 years old at event); or
 End stage renal disease (UMLS:ICD10CM:N18.6) (at least 18 years old at event).
 排除depressive disorder,: Any instance of 排除depressive disorder, occurred at least 1 day before any instance of CKD history
 Patients cannot have:
 any of the following:
 Depressive episode (UMLS:ICD10CM:F32); or
 Depression, unspecified (UMLS:ICD10CM:F32.A); or
 Other depressive episodes (UMLS:ICD10CM:F32.8); or
 Dysthymic disorder (UMLS:ICD10CM:F34.1); or
 Major depressive disorder, recurrent (UMLS:ICD10CM:F33).


 CKD history: The terms in this group occurred at any time
 Patients must have:
 any of the following:
 Chronic kidney disease (CKD) (UMLS:ICD10CM:N18) (at least 18 years old at event); or
 Chronic kidney disease, unspecified (UMLS:ICD10CM:N18.9) (at least 18 years old at event); or
 Chronic kidney disease, stage 3 (moderate) (UMLS:ICD10CM:N18.3) (at least 18 years old at event); or
 Chronic kidney disease, stage 3 unspecified (UMLS:ICD10CM:N18.30) (at least 18 years old at event); or
 Chronic kidney disease, stage 4 (severe) (UMLS:ICD10CM:N18.4) (at least 18 years old at event); or
 Chronic kidney disease, stage 2 (mild) (UMLS:ICD10CM:N18.2) (at least 18 years old at event); or
 Chronic kidney disease, stage 3a (UMLS:ICD10CM:N18.31) (at least 18 years old at event); or
 Chronic kidney disease, stage 3b (UMLS:ICD10CM:N18.32) (at least 18 years old at event); or
 Chronic kidney disease, stage 5 (UMLS:ICD10CM:N18.5) (at least 18 years old at event); or
 Chronic kidney disease, stage 1 (UMLS:ICD10CM:N18.1) (at least 18 years old at event); or
 End stage renal disease (UMLS:ICD10CM:N18.6) (at least 18 years old at event).
 Substance Use Disorders: Any instance of Substance Use Disorders occurred before or up to 5 years after any instance of CKD history
 Patients cannot have:
 Mental and behavioral disorders due to psychoactive substance use (UMLS:ICD10CM:F10-F19).


 CKD history: The terms in this group occurred at any time
 Patients must have:
 any of the following:
 Chronic kidney disease (CKD) (UMLS:ICD10CM:N18) (at least 18 years old at event); or
 Chronic kidney disease, unspecified (UMLS:ICD10CM:N18.9) (at least 18 years old at event); or
 Chronic kidney disease, stage 3 (moderate) (UMLS:ICD10CM:N18.3) (at least 18 years old at event); or
 Chronic kidney disease, stage 3 unspecified (UMLS:ICD10CM:N18.30) (at least 18 years old at event); or
 Chronic kidney disease, stage 4 (severe) (UMLS:ICD10CM:N18.4) (at least 18 years old at event); or
 Chronic kidney disease, stage 2 (mild) (UMLS:ICD10CM:N18.2) (at least 18 years old at event); or
 Chronic kidney disease, stage 3a (UMLS:ICD10CM:N18.31) (at least 18 years old at event); or
 Chronic kidney disease, stage 3b (UMLS:ICD10CM:N18.32) (at least 18 years old at event); or
 Chronic kidney disease, stage 5 (UMLS:ICD10CM:N18.5) (at least 18 years old at event); or
 Chronic kidney disease, stage 1 (UMLS:ICD10CM:N18.1) (at least 18 years old at event); or
 End stage renal disease (UMLS:ICD10CM:N18.6) (at least 18 years old at event).
 排除stroke or ICH or head injury: Any instance of 排除stroke or ICH or head injury occurred at least 1 day before any instance of CKD history
 Patients cannot have:
 any of the following:
 Cerebral infarction (UMLS:ICD10CM:I63); or
 Cerebral infarction due to unspecified occlusion or stenosis of unspecified cerebral artery (UMLS:ICD10CM:I63.50); or
 Cerebral infarction, unspecified (UMLS:ICD10CM:I63.9); or
 Cerebral infarction due to unspecified occlusion or stenosis of cerebral arteries (UMLS:ICD10CM:I63.5); or
 Other cerebral infarction (UMLS:ICD10CM:I63.8); or
 Cerebral infarction due to embolism of cerebral arteries (UMLS:ICD10CM:I63.4); or
 Cerebral infarction due to unspecified occlusion or stenosis of middle cerebral artery (UMLS:ICD10CM:I63.51); or
 Cerebral infarction due to thrombosis of cerebral arteries (UMLS:ICD10CM:I63.3); or
 Other and unspecified nontraumatic intracranial hemorrhage (UMLS:ICD10CM:I62); or
 Nontraumatic subdural hemorrhage (UMLS:ICD10CM:I62.0); or
 Nontraumatic subdural hemorrhage, unspecified (UMLS:ICD10CM:I62.00); or
 Nontraumatic intracranial hemorrhage, unspecified (UMLS:ICD10CM:I62.9); or
 Nontraumatic acute subdural hemorrhage (UMLS:ICD10CM:I62.01); or
 Nontraumatic chronic subdural hemorrhage (UMLS:ICD10CM:I62.03); or
 Nontraumatic extradural hemorrhage (UMLS:ICD10CM:I62.1); or
 Nontraumatic subacute subdural hemorrhage (UMLS:ICD10CM:I62.02); or
 Other and unspecified injuries of head (UMLS:ICD10CM:S09).


 CKD history: The terms in this group occurred at any time
 Patients must have:
 any of the following:
 Chronic kidney disease (CKD) (UMLS:ICD10CM:N18) (at least 18 years old at event); or
 Chronic kidney disease, unspecified (UMLS:ICD10CM:N18.9) (at least 18 years old at event); or
 Chronic kidney disease, stage 3 (moderate) (UMLS:ICD10CM:N18.3) (at least 18 years old at event); or
 Chronic kidney disease, stage 3 unspecified (UMLS:ICD10CM:N18.30) (at least 18 years old at event); or
 Chronic kidney disease, stage 4 (severe) (UMLS:ICD10CM:N18.4) (at least 18 years old at event); or
 Chronic kidney disease, stage 2 (mild) (UMLS:ICD10CM:N18.2) (at least 18 years old at event); or
 Chronic kidney disease, stage 3a (UMLS:ICD10CM:N18.31) (at least 18 years old at event); or
 Chronic kidney disease, stage 3b (UMLS:ICD10CM:N18.32) (at least 18 years old at event); or
 Chronic kidney disease, stage 5 (UMLS:ICD10CM:N18.5) (at least 18 years old at event); or
 Chronic kidney disease, stage 1 (UMLS:ICD10CM:N18.1) (at least 18 years old at event); or
 End stage renal disease (UMLS:ICD10CM:N18.6) (at least 18 years old at event).
 排除三年內mortality: Any instance of 排除三年內mortality occurred within 3 years on or after any instance of CKD history
 Patients cannot have:
 Deceased (Deceased).

### Query Criteria for Cohort 2 (query name: ###control V2 (>50, depression))

Patients must have:
 Age (Age) (at least 50 years (most recent occurrence)).

All the following must be satisfied:

 Visit>2: The terms in this group occurred between Jan 1, 2010 and Dec 31, 2019 (Greater than or equal to 2 instances)
 Patients must have:
 Visit (TNX:Visit).


 CKD history: The terms in this group occurred at any time
 Patients must have:
 any of the following:
 Chronic kidney disease (CKD) (UMLS:ICD10CM:N18) (at least 50 years old at event); or
 Chronic kidney disease, unspecified (UMLS:ICD10CM:N18.9) (at least 50 years old at event); or
 Chronic kidney disease, stage 3 (moderate) (UMLS:ICD10CM:N18.3) (at least 50 years old at event); or
 Chronic kidney disease, stage 3 unspecified (UMLS:ICD10CM:N18.30) (at least 50 years old at event); or
 Chronic kidney disease, stage 4 (severe) (UMLS:ICD10CM:N18.4) (at least 50 years old at event); or
 Chronic kidney disease, stage 2 (mild) (UMLS:ICD10CM:N18.2) (at least 50 years old at event); or
 Chronic kidney disease, stage 3a (UMLS:ICD10CM:N18.31) (at least 50 years old at event); or
 Chronic kidney disease, stage 3b (UMLS:ICD10CM:N18.32) (at least 50 years old at event); or
 Chronic kidney disease, stage 5 (UMLS:ICD10CM:N18.5) (at least 50 years old at event); or
 Chronic kidney disease, stage 1 (UMLS:ICD10CM:N18.1) (at least 50 years old at event); or
 End stage renal disease (UMLS:ICD10CM:N18.6) (at least 50 years old at event).
 在CKD三個月內vitamin D>30: Any instance of 在CKD三個月內vitamin D>30 occurred within 3 months on or after any instance of CKD history
 Patients must have:
 any of the following:
 Calcidiol [Mass/volume] in Serum or Plasma (TNX:9034) (at least 30.00 ng/mL); or
 Vitamin D+Metabolites [Mass/volume] in Serum or Plasma (UMLS:LNC:35365-6) (at least 30.00 ng/mL); or
 Calcidiol+ercalcidiol [Mass/volume] in Serum, Plasma or Blood (TNX:LG25965-1) (at least 30.00 ng/mL).


 CKD history: The terms in this group occurred at any time
 Patients must have:
 any of the following:
 Chronic kidney disease (CKD) (UMLS:ICD10CM:N18) (at least 18 years old at event); or
 Chronic kidney disease, unspecified (UMLS:ICD10CM:N18.9) (at least 18 years old at event); or
 Chronic kidney disease, stage 3 (moderate) (UMLS:ICD10CM:N18.3) (at least 18 years old at event); or
 Chronic kidney disease, stage 3 unspecified (UMLS:ICD10CM:N18.30) (at least 18 years old at event); or
 Chronic kidney disease, stage 4 (severe) (UMLS:ICD10CM:N18.4) (at least 18 years old at event); or
 Chronic kidney disease, stage 2 (mild) (UMLS:ICD10CM:N18.2) (at least 18 years old at event); or
 Chronic kidney disease, stage 3a (UMLS:ICD10CM:N18.31) (at least 18 years old at event); or
 Chronic kidney disease, stage 3b (UMLS:ICD10CM:N18.32) (at least 18 years old at event); or
 Chronic kidney disease, stage 5 (UMLS:ICD10CM:N18.5) (at least 18 years old at event); or
 Chronic kidney disease, stage 1 (UMLS:ICD10CM:N18.1) (at least 18 years old at event); or
 End stage renal disease (UMLS:ICD10CM:N18.6) (at least 18 years old at event).
 排除在CKD三個月內vitamin D<29: Any instance of 排除在CKD三個月內vitamin D<29 occurred within 3 months on or after any instance of CKD history
 Patients cannot have:
 any of the following:
 Calcidiol+ercalcidiol [Mass/volume] in Serum, Plasma or Blood (TNX:LG25965-1) (at most 29.00 ng/mL); or
 Calcidiol [Mass/volume] in Serum or Plasma (TNX:9034) (at most 29.00 ng/mL); or
 Vitamin D+Metabolites [Mass/volume] in Serum or Plasma (UMLS:LNC:35365-6) (at most 29.00 ng/mL).


 CKD history: The terms in this group occurred at any time
 Patients must have:
 any of the following:
 Chronic kidney disease (CKD) (UMLS:ICD10CM:N18) (at least 18 years old at event); or
 Chronic kidney disease, unspecified (UMLS:ICD10CM:N18.9) (at least 18 years old at event); or
 Chronic kidney disease, stage 3 (moderate) (UMLS:ICD10CM:N18.3) (at least 18 years old at event); or
 Chronic kidney disease, stage 3 unspecified (UMLS:ICD10CM:N18.30) (at least 18 years old at event); or
 Chronic kidney disease, stage 4 (severe) (UMLS:ICD10CM:N18.4) (at least 18 years old at event); or
 Chronic kidney disease, stage 2 (mild) (UMLS:ICD10CM:N18.2) (at least 18 years old at event); or
 Chronic kidney disease, stage 3a (UMLS:ICD10CM:N18.31) (at least 18 years old at event); or
 Chronic kidney disease, stage 3b (UMLS:ICD10CM:N18.32) (at least 18 years old at event); or
 Chronic kidney disease, stage 5 (UMLS:ICD10CM:N18.5) (at least 18 years old at event); or
 Chronic kidney disease, stage 1 (UMLS:ICD10CM:N18.1) (at least 18 years old at event); or
 End stage renal disease (UMLS:ICD10CM:N18.6) (at least 18 years old at event).
 排除cognitive impairment: Any instance of 排除cognitive impairment occurred at least 1 day before any instance of CKD history
 Patients cannot have:
 any of the following:
 Human immunodeficiency virus [HIV] disease (UMLS:ICD10CM:B20); or
 Mild cognitive impairment of uncertain or unknown etiology (UMLS:ICD10CM:G31.84); or
 Other mental disorders due to known physiological condition (UMLS:ICD10CM:F06); or
 Other specified mental disorders due to known physiological condition (UMLS:ICD10CM:F06.8); or
 Other disorders of psychological development (UMLS:ICD10CM:F88); or
 Individual Psychotherapy, Cognitive (UMLS:ICD10PCS:GZ52ZZZ); or
 Mild neurocognitive disorder due to known physiological condition (UMLS:ICD10CM:F06.7); or
 Unspecified mental disorder due to known physiological condition (UMLS:ICD10CM:F09).


 CKD history: The terms in this group occurred at any time
 Patients must have:
 any of the following:
 Chronic kidney disease (CKD) (UMLS:ICD10CM:N18) (at least 18 years old at event); or
 Chronic kidney disease, unspecified (UMLS:ICD10CM:N18.9) (at least 18 years old at event); or
 Chronic kidney disease, stage 3 (moderate) (UMLS:ICD10CM:N18.3) (at least 18 years old at event); or
 Chronic kidney disease, stage 3 unspecified (UMLS:ICD10CM:N18.30) (at least 18 years old at event); or
 Chronic kidney disease, stage 4 (severe) (UMLS:ICD10CM:N18.4) (at least 18 years old at event); or
 Chronic kidney disease, stage 2 (mild) (UMLS:ICD10CM:N18.2) (at least 18 years old at event); or
 Chronic kidney disease, stage 3a (UMLS:ICD10CM:N18.31) (at least 18 years old at event); or
 Chronic kidney disease, stage 3b (UMLS:ICD10CM:N18.32) (at least 18 years old at event); or
 Chronic kidney disease, stage 5 (UMLS:ICD10CM:N18.5) (at least 18 years old at event); or
 Chronic kidney disease, stage 1 (UMLS:ICD10CM:N18.1) (at least 18 years old at event); or
 End stage renal disease (UMLS:ICD10CM:N18.6) (at least 18 years old at event).
 排除dementia，Alzheimer's disease, Parkinsom disease: Any instance of 排除dementia，Alzheimer's disease, Parkinsom disease occurred at least 1 day before any instance of CKD history
 Patients cannot have:
 any of the following:
 Unspecified dementia (UMLS:ICD10CM:F03); or
 Unspecified dementia, unspecified severity, without behavioral disturbance, psychotic disturbance, mood disturbance, and anxiety (UMLS:ICD10CM:F03.90); or
 Dementia in other diseases classified elsewhere, unspecified severity, without behavioral disturbance, psychotic disturbance, mood disturbance, and anxiety (UMLS:ICD10CM:F02.80); or
 Psychotic disorder with hallucinations due to known physiological condition (UMLS:ICD10CM:F06.0); or
 Alzheimer's disease (UMLS:ICD10CM:G30); or
 Parkinson's disease (UMLS:ICD10CM:G20); or
 Delirium due to known physiological condition (UMLS:ICD10CM:F05); or
 Alzheimer's disease (UMLS:ICD10CM:G30); or
 Dementia in other diseases classified elsewhere, unspecified severity, without behavioral disturbance, psychotic disturbance, mood disturbance, and anxiety (UMLS:ICD10CM:F02.80); or
 Alzheimer's disease, unspecified (UMLS:ICD10CM:G30.9); or
 Other Alzheimer's disease (UMLS:ICD10CM:G30.8); or
 Delirium due to known physiological condition (UMLS:ICD10CM:F05); or
 Psychotic disorder with delusions due to known physiological condition (UMLS:ICD10CM:F06.2); or
 Alzheimer's disease with late onset (UMLS:ICD10CM:G30.1); or
 Alzheimer's disease with early onset (UMLS:ICD10CM:G30.0); or
 Alzheimer's disease with late onset (UMLS:ICD10CM:G30.1); or
 Other Alzheimer's disease (UMLS:ICD10CM:G30.8); or
 Alzheimer's disease, unspecified (UMLS:ICD10CM:G30.9); or
 Dementia in other diseases classified elsewhere, mild (UMLS:ICD10CM:F02.A); or
 Dementia in other diseases classified elsewhere, severe (UMLS:ICD10CM:F02.C); or
 Dementia in other diseases classified elsewhere, mild, without behavioral disturbance, psychotic disturbance, mood disturbance, and anxiety (UMLS:ICD10CM:F02.A0); or
 Dementia in other diseases classified elsewhere, moderate, without behavioral disturbance, psychotic disturbance, mood disturbance, and anxiety (UMLS:ICD10CM:F02.B0); or
 Dementia in other diseases classified elsewhere, unspecified severity, with mood disturbance (UMLS:ICD10CM:F02.83); or
 Dementia in other diseases classified elsewhere, unspecified severity, with agitation (UMLS:ICD10CM:F02.811); or
 Dementia in other diseases classified elsewhere, moderate (UMLS:ICD10CM:F02.B); or
 Dementia in other diseases classified elsewhere, unspecified severity, with other behavioral disturbance (UMLS:ICD10CM:F02.818); or
 Dementia in other diseases classified elsewhere, unspecified severity, with behavioral disturbance (UMLS:ICD10CM:F02.81); or
 Unspecified dementia (UMLS:ICD10CM:F03); or
 Unspecified dementia, unspecified severity, without behavioral disturbance, psychotic disturbance, mood disturbance, and anxiety (UMLS:ICD10CM:F03.90); or
 Dementia in other diseases classified elsewhere, unspecified severity, without behavioral disturbance, psychotic disturbance, mood disturbance, and anxiety (UMLS:ICD10CM:F02.80); or
 Psychotic disorder with hallucinations due to known physiological condition (UMLS:ICD10CM:F06.0); or
 Dementia in other diseases classified elsewhere (UMLS:ICD10CM:F02); or
 Dementia in other diseases classified elsewhere, unspecified severity (UMLS:ICD10CM:F02.8).


 排除renal transplanation: The terms in this group occurred at any time
 Patients cannot have:
 Renal Transplantation Procedures (UMLS:CPT:1008098).


 CKD history: The terms in this group occurred at any time
 Patients must have:
 any of the following:
 Chronic kidney disease (CKD) (UMLS:ICD10CM:N18) (at least 18 years old at event); or
 Chronic kidney disease, unspecified (UMLS:ICD10CM:N18.9) (at least 18 years old at event); or
 Chronic kidney disease, stage 3 (moderate) (UMLS:ICD10CM:N18.3) (at least 18 years old at event); or
 Chronic kidney disease, stage 3 unspecified (UMLS:ICD10CM:N18.30) (at least 18 years old at event); or
 Chronic kidney disease, stage 4 (severe) (UMLS:ICD10CM:N18.4) (at least 18 years old at event); or
 Chronic kidney disease, stage 2 (mild) (UMLS:ICD10CM:N18.2) (at least 18 years old at event); or
 Chronic kidney disease, stage 3a (UMLS:ICD10CM:N18.31) (at least 18 years old at event); or
 Chronic kidney disease, stage 3b (UMLS:ICD10CM:N18.32) (at least 18 years old at event); or
 Chronic kidney disease, stage 5 (UMLS:ICD10CM:N18.5) (at least 18 years old at event); or
 Chronic kidney disease, stage 1 (UMLS:ICD10CM:N18.1) (at least 18 years old at event); or
 End stage renal disease (UMLS:ICD10CM:N18.6) (at least 18 years old at event).
 排除vascular dementia: Any instance of 排除vascular dementia occurred at least 1 day before any instance of CKD history
 Patients cannot have:
 any of the following:
 Vascular dementia (UMLS:ICD10CM:F01); or
 Cerebral atherosclerosis (UMLS:ICD10CM:I67.2); or
 Vascular dementia, unspecified severity, without behavioral disturbance, psychotic disturbance, mood disturbance, and anxiety (UMLS:ICD10CM:F01.50); or
 Vascular dementia, unspecified severity (UMLS:ICD10CM:F01.5); or
 Vascular dementia, mild (UMLS:ICD10CM:F01.A); or
 Vascular dementia, moderate (UMLS:ICD10CM:F01.B); or
 Vascular dementia, mild, without behavioral disturbance, psychotic disturbance, mood disturbance, and anxiety (UMLS:ICD10CM:F01.A0); or
 Vascular dementia, moderate, without behavioral disturbance, psychotic disturbance, mood disturbance, and anxiety (UMLS:ICD10CM:F01.B0); or
 Vascular dementia, severe (UMLS:ICD10CM:F01.C).


 CKD history: The terms in this group occurred at any time
 Patients must have:
 any of the following:
 Chronic kidney disease (CKD) (UMLS:ICD10CM:N18) (at least 18 years old at event); or
 Chronic kidney disease, unspecified (UMLS:ICD10CM:N18.9) (at least 18 years old at event); or
 Chronic kidney disease, stage 3 (moderate) (UMLS:ICD10CM:N18.3) (at least 18 years old at event); or
 Chronic kidney disease, stage 3 unspecified (UMLS:ICD10CM:N18.30) (at least 18 years old at event); or
 Chronic kidney disease, stage 4 (severe) (UMLS:ICD10CM:N18.4) (at least 18 years old at event); or
 Chronic kidney disease, stage 2 (mild) (UMLS:ICD10CM:N18.2) (at least 18 years old at event); or
 Chronic kidney disease, stage 3a (UMLS:ICD10CM:N18.31) (at least 18 years old at event); or
 Chronic kidney disease, stage 3b (UMLS:ICD10CM:N18.32) (at least 18 years old at event); or
 Chronic kidney disease, stage 5 (UMLS:ICD10CM:N18.5) (at least 18 years old at event); or
 Chronic kidney disease, stage 1 (UMLS:ICD10CM:N18.1) (at least 18 years old at event); or
 End stage renal disease (UMLS:ICD10CM:N18.6) (at least 18 years old at event).
 排除Schizophrenia: Any instance of 排除Schizophrenia occurred at least 1 day before any instance of CKD history
 Patients cannot have:
 any of the following:
 Schizophrenia (UMLS:ICD10CM:F20); or
 Schizophrenia, schizotypal, delusional, and other non-mood psychotic disorders (UMLS:ICD10CM:F20-F29); or
 Schizophrenia, unspecified (UMLS:ICD10CM:F20.9); or
 Personal history of other specified conditions (UMLS:ICD10CM:Z87.898); or
 Pervasive developmental disorders (UMLS:ICD10CM:F84); or
 Unspecified psychosis not due to a substance or known physiological condition (UMLS:ICD10CM:F29).


 CKD history: The terms in this group occurred at any time
 Patients must have:
 any of the following:
 Chronic kidney disease (CKD) (UMLS:ICD10CM:N18) (at least 18 years old at event); or
 Chronic kidney disease, unspecified (UMLS:ICD10CM:N18.9) (at least 18 years old at event); or
 Chronic kidney disease, stage 3 (moderate) (UMLS:ICD10CM:N18.3) (at least 18 years old at event); or
 Chronic kidney disease, stage 3 unspecified (UMLS:ICD10CM:N18.30) (at least 18 years old at event); or
 Chronic kidney disease, stage 4 (severe) (UMLS:ICD10CM:N18.4) (at least 18 years old at event); or
 Chronic kidney disease, stage 2 (mild) (UMLS:ICD10CM:N18.2) (at least 18 years old at event); or
 Chronic kidney disease, stage 3a (UMLS:ICD10CM:N18.31) (at least 18 years old at event); or
 Chronic kidney disease, stage 3b (UMLS:ICD10CM:N18.32) (at least 18 years old at event); or
 Chronic kidney disease, stage 5 (UMLS:ICD10CM:N18.5) (at least 18 years old at event); or
 Chronic kidney disease, stage 1 (UMLS:ICD10CM:N18.1) (at least 18 years old at event); or
 End stage renal disease (UMLS:ICD10CM:N18.6) (at least 18 years old at event).
 排除biploar disorders: Any instance of 排除biploar disorders occurred at least 1 day before any instance of CKD history
 Patients cannot have:
 any of the following:
 Bipolar disorder (UMLS:ICD10CM:F31); or
 Personal history of other mental and behavioral disorders (UMLS:ICD10CM:Z86.59); or
 Bipolar II disorder (UMLS:ICD10CM:F31.81); or
 Bipolar disorder, current episode manic severe with psychotic features (UMLS:ICD10CM:F31.2); or
 Bipolar disorder, current episode mixed, unspecified (UMLS:ICD10CM:F31.60).


 CKD history: The terms in this group occurred at any time
 Patients must have:
 any of the following:
 Chronic kidney disease (CKD) (UMLS:ICD10CM:N18) (at least 18 years old at event); or
 Chronic kidney disease, unspecified (UMLS:ICD10CM:N18.9) (at least 18 years old at event); or
 Chronic kidney disease, stage 3 (moderate) (UMLS:ICD10CM:N18.3) (at least 18 years old at event); or
 Chronic kidney disease, stage 3 unspecified (UMLS:ICD10CM:N18.30) (at least 18 years old at event); or
 Chronic kidney disease, stage 4 (severe) (UMLS:ICD10CM:N18.4) (at least 18 years old at event); or
 Chronic kidney disease, stage 2 (mild) (UMLS:ICD10CM:N18.2) (at least 18 years old at event); or
 Chronic kidney disease, stage 3a (UMLS:ICD10CM:N18.31) (at least 18 years old at event); or
 Chronic kidney disease, stage 3b (UMLS:ICD10CM:N18.32) (at least 18 years old at event); or
 Chronic kidney disease, stage 5 (UMLS:ICD10CM:N18.5) (at least 18 years old at event); or
 Chronic kidney disease, stage 1 (UMLS:ICD10CM:N18.1) (at least 18 years old at event); or
 End stage renal disease (UMLS:ICD10CM:N18.6) (at least 18 years old at event).
 排除depressive disorder,: Any instance of 排除depressive disorder, occurred at least 1 day before any instance of CKD history
 Patients cannot have:
 any of the following:
 Depressive episode (UMLS:ICD10CM:F32); or
 Depression, unspecified (UMLS:ICD10CM:F32.A); or
 Other depressive episodes (UMLS:ICD10CM:F32.8); or
 Dysthymic disorder (UMLS:ICD10CM:F34.1); or
 Major depressive disorder, recurrent (UMLS:ICD10CM:F33).


 CKD history: The terms in this group occurred at any time
 Patients must have:
 any of the following:
 Chronic kidney disease (CKD) (UMLS:ICD10CM:N18) (at least 18 years old at event); or
 Chronic kidney disease, unspecified (UMLS:ICD10CM:N18.9) (at least 18 years old at event); or
 Chronic kidney disease, stage 3 (moderate) (UMLS:ICD10CM:N18.3) (at least 18 years old at event); or
 Chronic kidney disease, stage 3 unspecified (UMLS:ICD10CM:N18.30) (at least 18 years old at event); or
 Chronic kidney disease, stage 4 (severe) (UMLS:ICD10CM:N18.4) (at least 18 years old at event); or
 Chronic kidney disease, stage 2 (mild) (UMLS:ICD10CM:N18.2) (at least 18 years old at event); or
 Chronic kidney disease, stage 3a (UMLS:ICD10CM:N18.31) (at least 18 years old at event); or
 Chronic kidney disease, stage 3b (UMLS:ICD10CM:N18.32) (at least 18 years old at event); or
 Chronic kidney disease, stage 5 (UMLS:ICD10CM:N18.5) (at least 18 years old at event); or
 Chronic kidney disease, stage 1 (UMLS:ICD10CM:N18.1) (at least 18 years old at event); or
 End stage renal disease (UMLS:ICD10CM:N18.6) (at least 18 years old at event).
 Substance Use Disorders: Any instance of Substance Use Disorders occurred before or up to 5 years after any instance of CKD history
 Patients cannot have:
 Mental and behavioral disorders due to psychoactive substance use (UMLS:ICD10CM:F10-F19).


 CKD history: The terms in this group occurred at any time
 Patients must have:
 any of the following:
 Chronic kidney disease (CKD) (UMLS:ICD10CM:N18) (at least 18 years old at event); or
 Chronic kidney disease, unspecified (UMLS:ICD10CM:N18.9) (at least 18 years old at event); or
 Chronic kidney disease, stage 3 (moderate) (UMLS:ICD10CM:N18.3) (at least 18 years old at event); or
 Chronic kidney disease, stage 3 unspecified (UMLS:ICD10CM:N18.30) (at least 18 years old at event); or
 Chronic kidney disease, stage 4 (severe) (UMLS:ICD10CM:N18.4) (at least 18 years old at event); or
 Chronic kidney disease, stage 2 (mild) (UMLS:ICD10CM:N18.2) (at least 18 years old at event); or
 Chronic kidney disease, stage 3a (UMLS:ICD10CM:N18.31) (at least 18 years old at event); or
 Chronic kidney disease, stage 3b (UMLS:ICD10CM:N18.32) (at least 18 years old at event); or
 Chronic kidney disease, stage 5 (UMLS:ICD10CM:N18.5) (at least 18 years old at event); or
 Chronic kidney disease, stage 1 (UMLS:ICD10CM:N18.1) (at least 18 years old at event); or
 End stage renal disease (UMLS:ICD10CM:N18.6) (at least 18 years old at event).
 排除stroke or ICH or head injury: Any instance of 排除stroke or ICH or head injury occurred at least 1 day before any instance of CKD history
 Patients cannot have:
 any of the following:
 Cerebral infarction (UMLS:ICD10CM:I63); or
 Cerebral infarction due to unspecified occlusion or stenosis of unspecified cerebral artery (UMLS:ICD10CM:I63.50); or
 Cerebral infarction, unspecified (UMLS:ICD10CM:I63.9); or
 Cerebral infarction due to unspecified occlusion or stenosis of cerebral arteries (UMLS:ICD10CM:I63.5); or
 Other cerebral infarction (UMLS:ICD10CM:I63.8); or
 Cerebral infarction due to embolism of cerebral arteries (UMLS:ICD10CM:I63.4); or
 Cerebral infarction due to unspecified occlusion or stenosis of middle cerebral artery (UMLS:ICD10CM:I63.51); or
 Cerebral infarction due to thrombosis of cerebral arteries (UMLS:ICD10CM:I63.3); or
 Other and unspecified nontraumatic intracranial hemorrhage (UMLS:ICD10CM:I62); or
 Nontraumatic subdural hemorrhage (UMLS:ICD10CM:I62.0); or
 Nontraumatic subdural hemorrhage, unspecified (UMLS:ICD10CM:I62.00); or
 Nontraumatic intracranial hemorrhage, unspecified (UMLS:ICD10CM:I62.9); or
 Nontraumatic acute subdural hemorrhage (UMLS:ICD10CM:I62.01); or
 Nontraumatic chronic subdural hemorrhage (UMLS:ICD10CM:I62.03); or
 Nontraumatic extradural hemorrhage (UMLS:ICD10CM:I62.1); or
 Nontraumatic subacute subdural hemorrhage (UMLS:ICD10CM:I62.02); or
 Other and unspecified injuries of head (UMLS:ICD10CM:S09).


 CKD history: The terms in this group occurred at any time
 Patients must have:
 any of the following:
 Chronic kidney disease (CKD) (UMLS:ICD10CM:N18) (at least 18 years old at event); or
 Chronic kidney disease, unspecified (UMLS:ICD10CM:N18.9) (at least 18 years old at event); or
 Chronic kidney disease, stage 3 (moderate) (UMLS:ICD10CM:N18.3) (at least 18 years old at event); or
 Chronic kidney disease, stage 3 unspecified (UMLS:ICD10CM:N18.30) (at least 18 years old at event); or
 Chronic kidney disease, stage 4 (severe) (UMLS:ICD10CM:N18.4) (at least 18 years old at event); or
 Chronic kidney disease, stage 2 (mild) (UMLS:ICD10CM:N18.2) (at least 18 years old at event); or
 Chronic kidney disease, stage 3a (UMLS:ICD10CM:N18.31) (at least 18 years old at event); or
 Chronic kidney disease, stage 3b (UMLS:ICD10CM:N18.32) (at least 18 years old at event); or
 Chronic kidney disease, stage 5 (UMLS:ICD10CM:N18.5) (at least 18 years old at event); or
 Chronic kidney disease, stage 1 (UMLS:ICD10CM:N18.1) (at least 18 years old at event); or
 End stage renal disease (UMLS:ICD10CM:N18.6) (at least 18 years old at event).
 排除三年內mortality: Any instance of 排除三年內mortality occurred within 3 years on or after any instance of CKD history
 Patients cannot have:
 Deceased (Deceased).

# Appendix B – Text Representation of the Analysis Setup

This section contains the Index Event definition for each cohort.

The index event for Cohort 1 (query name: ###VDD v2 (>50, depression) is defined as the following:

All the following must be satisfied:

 CKD history: The terms in this group occurred at any time
 Patients must have:
 any of the following:
 Chronic kidney disease (CKD) (UMLS:ICD10CM:N18) (at least 18 years old at event); or
 Chronic kidney disease, unspecified (UMLS:ICD10CM:N18.9) (at least 18 years old at event); or
 Chronic kidney disease, stage 3 (moderate) (UMLS:ICD10CM:N18.3) (at least 18 years old at event); or
 Chronic kidney disease, stage 3 unspecified (UMLS:ICD10CM:N18.30) (at least 18 years old at event); or
 Chronic kidney disease, stage 4 (severe) (UMLS:ICD10CM:N18.4) (at least 18 years old at event); or
 Chronic kidney disease, stage 2 (mild) (UMLS:ICD10CM:N18.2) (at least 18 years old at event); or
 Chronic kidney disease, stage 3a (UMLS:ICD10CM:N18.31) (at least 18 years old at event); or
 Chronic kidney disease, stage 3b (UMLS:ICD10CM:N18.32) (at least 18 years old at event); or
 Chronic kidney disease, stage 5 (UMLS:ICD10CM:N18.5) (at least 18 years old at event); or
 Chronic kidney disease, stage 1 (UMLS:ICD10CM:N18.1) (at least 18 years old at event); or
 End stage renal disease (UMLS:ICD10CM:N18.6) (at least 18 years old at event).
 CKD三個月內vitamin D<20: Any instance of CKD三個月內vitamin D<20 occurred within 3 months on or after any instance of CKD history
 Patients must have:
 any of the following:
 Vitamin D+Metabolites [Mass/volume] in Serum or Plasma (UMLS:LNC:35365-6) (at most 20.00 ng/mL); or
 Calcidiol+ercalcidiol [Mass/volume] in Serum, Plasma or Blood (TNX:LG25965-1) (at most 20.00 ng/mL); or
 Calcidiol [Mass/volume] in Serum or Plasma (TNX:9034) (at most 20.00 ng/mL).

The index event for Cohort 2 (query name: ###control V2 (>50, depression)) is defined as the following:

All the following must be satisfied:

 CKD history: The terms in this group occurred at any time
 Patients must have:
 any of the following:
 Chronic kidney disease (CKD) (UMLS:ICD10CM:N18) (at least 50 years old at event); or
 Chronic kidney disease, unspecified (UMLS:ICD10CM:N18.9) (at least 50 years old at event); or
 Chronic kidney disease, stage 3 (moderate) (UMLS:ICD10CM:N18.3) (at least 50 years old at event); or
 Chronic kidney disease, stage 3 unspecified (UMLS:ICD10CM:N18.30) (at least 50 years old at event); or
 Chronic kidney disease, stage 4 (severe) (UMLS:ICD10CM:N18.4) (at least 50 years old at event); or
 Chronic kidney disease, stage 2 (mild) (UMLS:ICD10CM:N18.2) (at least 50 years old at event); or
 Chronic kidney disease, stage 3a (UMLS:ICD10CM:N18.31) (at least 50 years old at event); or
 Chronic kidney disease, stage 3b (UMLS:ICD10CM:N18.32) (at least 50 years old at event); or
 Chronic kidney disease, stage 5 (UMLS:ICD10CM:N18.5) (at least 50 years old at event); or
 Chronic kidney disease, stage 1 (UMLS:ICD10CM:N18.1) (at least 50 years old at event); or
 End stage renal disease (UMLS:ICD10CM:N18.6) (at least 50 years old at event).
 在CKD三個月內vitamin D>30: Any instance of 在CKD三個月內vitamin D>30 occurred within 3 months on or after any instance of CKD history
 Patients must have:
 any of the following:
 Calcidiol [Mass/volume] in Serum or Plasma (TNX:9034) (at least 30.00 ng/mL); or
 Vitamin D+Metabolites [Mass/volume] in Serum or Plasma (UMLS:LNC:35365-6) (at least 30.00 ng/mL); or
 Calcidiol+ercalcidiol [Mass/volume] in Serum, Plasma or Blood (TNX:LG25965-1) (at least 30.00 ng/mL).

# Appendix C – Text Representation of the Outcomes Definition

This analysis includes the following outcomes:

cognitive impairment
 Patients must have:
 any of the following:
 Mild cognitive impairment of uncertain or unknown etiology (UMLS:ICD10CM:G31.84); or
 Unspecified mental disorder due to known physiological condition (UMLS:ICD10CM:F09).

depression
 Patients must have:
 Depressive episode (UMLS:ICD10CM:F32).

dementia
 Patients must have:
 Unspecified dementia (UMLS:ICD10CM:F03).

Unnamed Outcome
 Patients must have:
 Cerebral infarction (UMLS:ICD10CM:I63).
